# Applications and implementation considerations for stepped-wedge designs in sub-Saharan Africa: a systematic review

**DOI:** 10.3389/fepid.2026.1529289

**Published:** 2026-03-25

**Authors:** Zaidat Adesola Musa, Folahanmi Tomiwa Akinsolu, Abideen Oluwarotimi Salako, Olunike Rebecca Abodunrin, Oluwabukola Mary Ola, Oliver Chukwujekwu Ezechi

**Affiliations:** 1 Clinical Sciences Department, Nigerian Institute Medical Research, Yaba, Lagos, Nigeria; 2 Center for Reproduction and Population Health Studies, Nigerian Institute Medical Research, Yaba, Lagos, Nigeria; 3 Department of Public Health, Faculty of Basic Medical and Health Sciences, Lead City University, Ibadan, Nigeria; 4 Department of Epidemiology, Conter for Global Health, School of Public Health, Nanjing Medical University, Nanjing, China

**Keywords:** cluster randomization, implementation science, public health research, stepped-wedge study design, sub-Saharan Africa

## Abstract

**Introduction:**

Stepped-wedge design (SWD) has gained prominence as a versatile research methodology, particularly in public health and implementation science, due to its ability to balance ethical concerns with methodological rigor. This systematic review aims to evaluate the implementation and effectiveness of SWD in sub-Saharan African (SSA) research contexts, focusing on the types of interventions, primary outcomes, and the unique geographic and cultural factors influencing the studies.

**Methodology:**

A systematic review protocol was developed following the Preferred Reporting Items for Systematic Reviews and Meta-Analyses (PRISMA) statement. The protocol was registered with the International Prospective Register of Systematic Reviews (PROSPERO) under identification number CRD42024530774. A comprehensive search strategy was employed to identify studies conducted in SSA using SWD from January 2000 to March 2024 across five electronic databases (PubMed, Web of Science, CINAHL, PsycINFO, and Cochrane Library), along with Google Scholar and citation tracking. Studies were included if they utilized SWD in SSA settings and reported relevant public health, clinical, or social interventions. Data were extracted on study characteristics, SWD implementation details, statistical methods, and sample size calculations. A total of 85 studies were included after screening 873 titles and abstracts and conducting full-text reviews of 93 articles.

**Results:**

The 85 studies included in the review spanned a wide range of health domains, including HIV/AIDS, maternal and child health, tuberculosis, and malaria, conducted across diverse SSA settings such as hospitals, communities, and schools. The studies involved a total of 1,895,788 participants, with sample sizes ranging from 17 to 780,000. Most studies (84.7%) were facility-based, while 15.3% were community-based. The number of clusters per study varied, with some studies using as few as four clusters, while others utilized up to 54 clusters. The number of steps ranged from two to twelve, depending on the complexity and scale of the intervention. Sample size calculations were often based on expected changes in primary outcomes, with many studies assuming an intra-cluster correlation coefficient to account for clustering effects. The SWD was primarily chosen to address ethical concerns, logistical challenges, and resource limitations. The review highlights significant variability in study designs, interventions, and outcomes, reflecting the adaptability of SWD to different contexts and challenges.

**Conclusion:**

The SWD has been effectively utilized in SSA research to evaluate a wide range of interventions across diverse settings, demonstrating its flexibility and suitability for addressing complex public health challenges. However, the review also identifies challenges related to study duration, logistical implementation, randomization processes, and statistical analysis, suggesting the need for careful planning and methodological rigor in future studies using SWD. The findings provide valuable insights for researchers and policymakers seeking to optimize the use of SWD in resource-limited settings, ensuring that interventions are both effective and ethically implemented.

**Systematic Reviews Registration:**

https://www.crd.york.ac.uk/PROSPERO/view/CRD42024530774, PROSPERO CRD42024530774.

## Introduction

Stepped-wedge study design (SWD) has emerged as a pivotal innovation in research methodology, particularly within public health and implementation science contexts (1). This design involves the sequential introduction of an intervention across multiple time periods, ensuring all participants eventually receive the intervention by the study's end (2). The SWD enables robust within-subject comparisons and is especially beneficial in scenarios where traditional randomization or withholding an intervention might be ethically or practically challenging (3). The methodology entails rolling out the intervention in stages to either individual participants or clusters of participants, with the sequence of rollout determined randomly (4). Data is collected at each step as new groups receive the intervention, allowing for a thorough analysis of intervention effectiveness by comparing data from control and intervention phases (5).

Recent years have seen a surge in the use of the SWD within sub-Sahran African (SSA) research settings (6–8). The continent offers a unique and diverse environment for public health research, shaped by varied cultural practices, disease burdens, and infrastructural challenges (9–11). These complexities demand flexible and innovative research methods capable of effectively evaluating and implementing public health interventions. The SWD, with its adaptability and ethical considerations, is well-suited to these settings, offering researchers a valuable tool for improving health outcomes across SSA (1, 12, 13).

**Figure 1:**
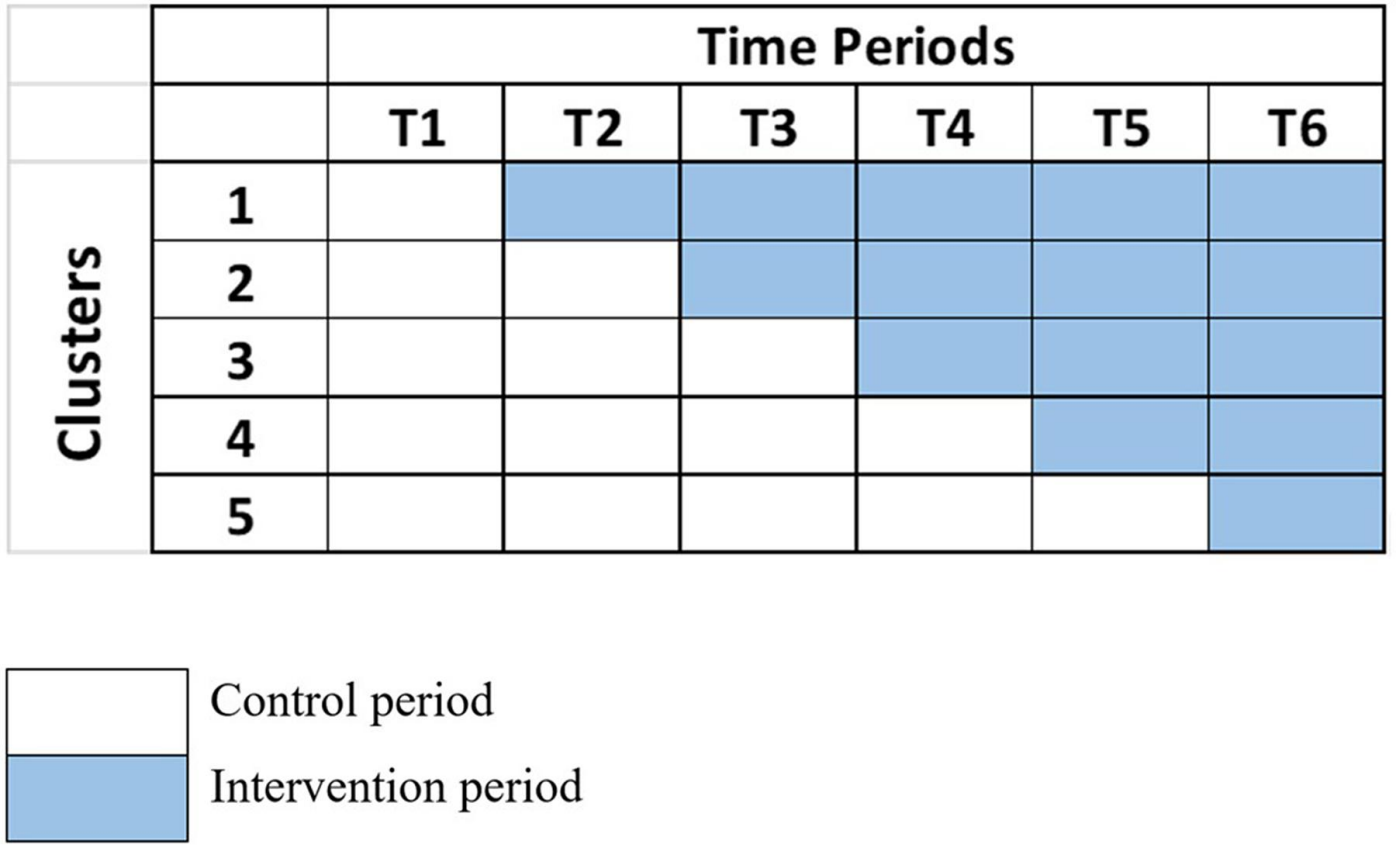
Diagrammatic illustration of a stepped-wedge design. This figure illustrates a typical stepped-wedge trial with five clusters observed over six time periods (T1–T6). All clusters begin in the control condition and transition sequentially to the intervention condition at predefined time points until all clusters receive the intervention. White cells indicate control periods, while shaded cells represent intervention periods. This design allows within-cluster and between-cluster comparisons over time.

There are specific scenarios where the SWD is preferable to traditional parallel designs. Firstly, when there is a belief that an intervention is more likely to benefit than harm, it may be unethical to withhold it from any participants or to remove it, as might occur in a crossover design (14). Secondly, logistical, political, practical, or financial constraints might necessitate a phased intervention rollout. In such cases, randomly determining the order in which participants receive the intervention is often ethically acceptable and beneficial for trial recruitment. For instance, a community-wide vaccination program targeting rural areas for a new influenza vaccine could be well-suited for a SWD. In this scenario, logistical and resource constraints might prevent the simultaneous rollout of the vaccine to all eligible participants across multiple villages. By using an SWD, the program can sequentially introduce the vaccine to different villages at staggered intervals. This approach allows for continuous monitoring of vaccine uptake, coverage, and potential adverse effects across different settings, providing valuable data for optimizing the implementation strategy. Additionally, the SWD ensures that all villages eventually receive the vaccine, addressing ethical concerns associated with withholding a potentially beneficial intervention. An example of the logistics of a SWD is shown in Figure 1, which shows a SWD with four steps. Data analysis to determine the overall effectiveness of the intervention subsequently involves comparison of the data points in the control section of the wedge with those in the intervention section.

Although SWDs are an increasingly powerful approach in public health, they have limitations that must be anticipated during planning. In real-world implementation, particularly in resource-constrained settings, these limitations can translate into operartional challenges, such as delayed crossover schedules, workforce or supply disruptions, or adaptions during roll-out. However, stepped-wedge evaluations do not consistently report whether such challenges led to protocol amendments, deviations from planned implementation, or compromised outcomes, creating uncertainty about when SWD may be suboptimal and how best to mitigate these risks. Stepped-wedge trials can also extend study duration compared with traditional parallel designs, particularly when outcomes are measured shortly after implementation (2, 3, 15). In addition, contamination between participants who have received the intervention and those awaiting roll-out may occur, and analyses can be complex due to time-varying effects and intra-cluster correlation, requiring appropriate analytic approaches (3, 8, 16, 19). Where outcome assessment is feasible, blinding assessors to intervention status may reduce information bias (17).

The primary motivations for employing the SWD in SSA research are to address ethical concerns regarding withholding potentially beneficial interventions and to manage the logistical challenges of simultaneous intervention implementation across diverse, resource-limited settings (3, 13, 18, 20). The design's capacity to accommodate phased implementations makes it particularly appealing in contexts such as infectious disease control, maternal and child health initiatives, and educational programs. The phased approach allows for adjustments based on initial findings, enhancing the intervention's overall effectiveness (3, 12, 20–22).

Despite the advantages of SWD, their effectiveness and applicability in SSA remain insufficiently synthesised. Given the unique environmental and socio-cultural conditions across SSA research settings, it is important to evaluate how SWD performs in these contextss (3, 7, 18, 20, 23). However, the current literature lacks a consolidated regional synthesis that simultaneously describes where SWD has been applied (by country and health domain), how designs have been operationalised, and what implementation challenges occurred during roll-out. In particular, operational challenges and protocol deviations are inconsistently reported, limiting practical guidance on when SWD may be suboptimal and how risks can be mitigated in resource-constrained settings. To address these gaps, this systematic review assesses the implementation, outcomes, and methodological considerations of stepped-wedge studies conducted in SSA, including the intervention types studied, primary outcomes measured, statistical methods used, and contextual factors that may influence implementation and effectiveness.

By synthesizing the existing evidence, this review seeks to provide a comprehensive understanding of the SWD's strengths and limitations in SSA research contexts. It also highlights the practical implications of using the SWD for policy-making and public health interventions in SSA, offering insights into best practices and strategies to maximize the design's potential in these unique settings.

## Methodology

### Protocol registration

A systematic review protocol was performed adhering to the guidelines outlined in the Meta- Analysis of Observational Studies in Epidemiology (MOOSE) for systematic reviews (24) and the Preferred Reporting Items for Systematic Reviews and Meta-Analyses (PRISMA) statement for reporting systematic reviews and meta-analyses (25). The investigators wrote a protocol and registered it with the International Prospective Register of Systematic Reviews (identification number CRD42024530774) in March 2024.

### Search strategy

The search strategy was designed to identify studies reporting stepped-wedge study design in SSA by systematically searching literature databases from January 2000 to March 2024. We searched PubMed, Web of Science, CINAHL, PsycINFO, and the Cochrane Library, alongside Google Scholar and citation searching. Searches were restricted to English-language publications due to feasibility constraints and to ensure consistent screening and extraction. Detailed search strategiesfor each database are reported in Supplementary File S1. The bibliographies of relevant reviews and eligible studies were also examined for additional sources. In addition, grey literature sources were consulted, including repositories of conference proceedings and university theses, and relevant expert sources.

### Studies eligibility criteria

In this systematic review, we focused exclusively on studies that utilized the SWD and were conducted within SSA countries. We included studies set in various environments, such as hospitals, communities, schools, and other public health-related contexts. Our review specifically considered studies that evaluated public health, clinical, or social interventions, with an emphasis on those that reported the effectiveness of these interventions and provided data on the applicability of the SWD in African settings.

To ensure the relevance and quality of the included studies, we only considered publications in English or French, covering a timeframe from the inception of each database up to the date of the final search.

We excluded studies that did not employ the SWD or used modified versions that significantly deviated from the standard SWD framework. Additionally, studies conducted outside of SSA or those focusing on non-SSA populations were not included. We also excluded studies that failed to report relevant outcomes or provided insufficient data for meaningful analysis. Furthermore, publications such as editorials, commentaries, opinion pieces, conference abstracts without full data, and non-peer-reviewed articles were excluded from this review.

### Studies selection

We used a comprehensive strategy to identify studies on SWD in SSA countries. All records were imported into Rayyan (Rayyan Systems Inc.) for deduplication and title/abstract screening. Three reviewers (O.M.O., O.R.A, and A.Z.M) read the titles and abstracts to determine each study's eligibility for full-text review. Studies were included if they described an original research trial that used a SWD approach incorporating all the following elements: clusters, randomization, and comparison with controls. Studies retrospectively analyzed as a stepped wedge when the study was not designed initially as such were excluded. Given the focus on cluster randomized trials, trials where individuals were the unit of randomization or the stepping level were excluded. Duplicate, serial, or follow-on publications for the same study were counted as one, although data extraction may have come from several sources.

Two separate reviewers (O.R.A. and O.M.O.) independently reviewed full-texts for data extraction based on the pre-defined criteria. When further clarification information was required, authors were contacted via email. Discrepancies were resolved by discussion or involvement of a third reviewer (F.T.A.).

### Data extraction

Three authors (A.Z.M, O.R.A., and O.M.O.) independently extracted data from published sources using a data extraction form. The extracted information included study identifiers (lead author and publication year), research area (subject areas, disease/domain, nature of intervention, setting, and country), motivation for using the stepped wedge design, general characteristics of the stepped wedge design (level of stepping, number of steps, number of clusters, total number of participants, and period between steps), methods of data analysis (primary outcome, comparisons, and statistical methods of data analysis), and selected items on quality of reporting. The data extraction form was piloted on ten articles and adjusted accordingly. Authors were contacted for complete reports for studies with only abstracts or protocol summaries. Additional information was obtained from the study authors where required. Differences in data extraction were resolved through discussion or referral to a fourth author (F.T.A).

### Ethical statement

No institutional review board approval was required for this study.

## Results

### Selection of studies

**Figure 2:**
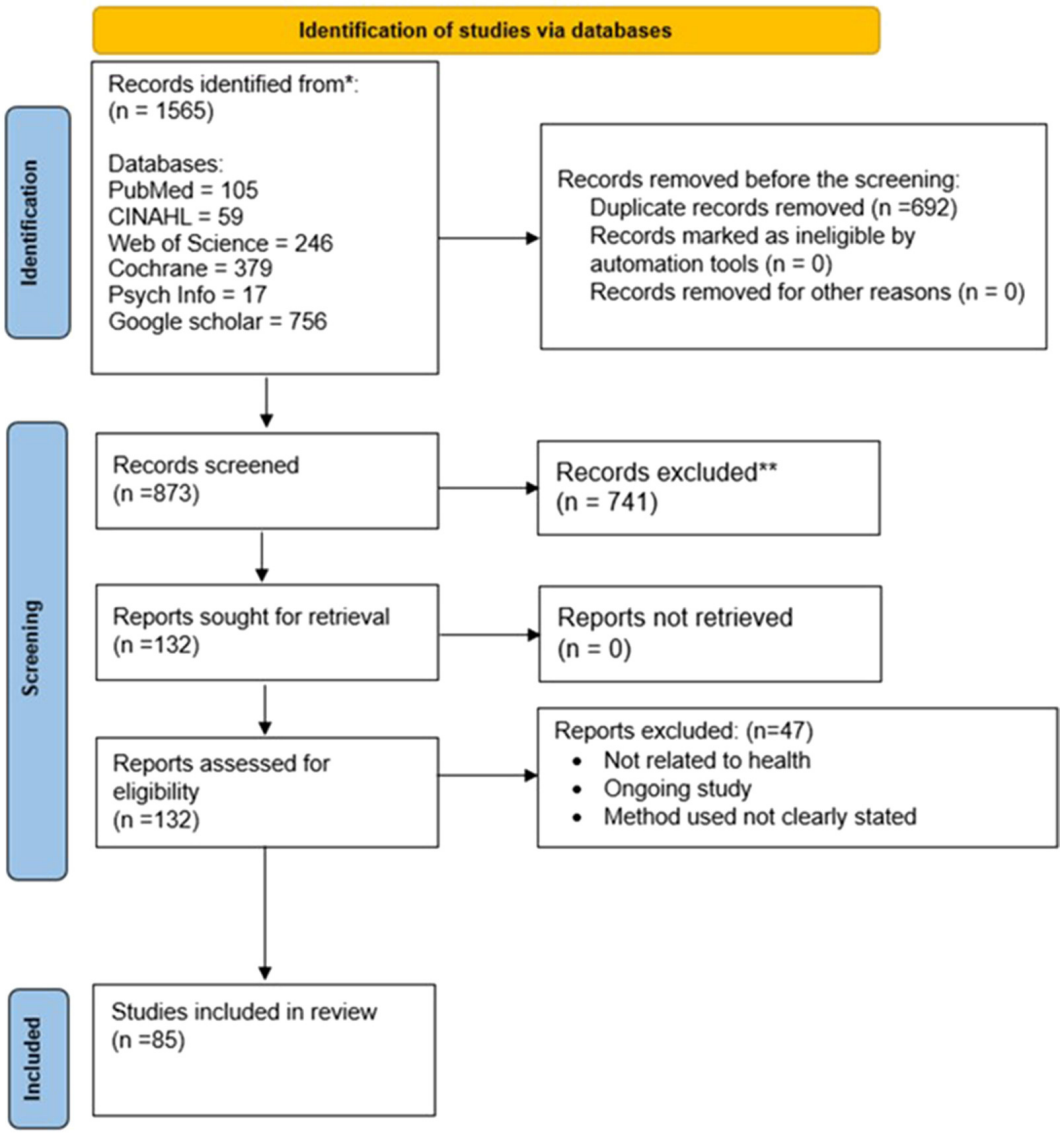
PRISMA flow diagram of study selection. The PRISMA flow diagram summarises the study identification, screening, eligibility assessment, and inclusion process. A total of 1,565 records were identified from databases and supplementary sources. After removal of duplicates, 873 records were screened, 132 full texts were assessed for eligibility, and 85 studies met the inclusion criteria for the systematic review.

2,562 articles were retrieved from five databases (PubMed, Web of Science, CINALH, PsychINFO, and Cochrane), Google Scholar and citation Searching. The search ranges from January 2000 to March, 2024. One thousand six hundred and eighty-nine articles were removed before screening the remaining 873 were screened for titles and abstracts. Of the 873 articles, 780 were excluded based on the study exclusion criteria. Furthermore, 93 articles were retrieved for full-text screening. Finally, after a thorough full-text screening, 85 articles met the inclusion criteria (26–110) (see Figure 2).

### Characteristics of included studies

The extracted data from the 85 studies are presented in Table 1. The studies included a total of 1,895,788 participants. The study's sample sizes range from 17 to 780,000. Of the 85 studies included, 72 (84.7%) were conducted in a facility-based (26, 27, 30–34, 36–47, 49, 50, 52–64, 66–75, 77–81, 83–96, 98–102, 104, 105, 107, 109), and 13 (15.3%) were community-based studies (28, 29, 35, 48, 51, 72, 76, 82, 97, 103, 106, 108, 110).

**Table 1:** Characteristics of included studies.

S/No	First author/year	Title	Country	Study settings	Sample size	Study duration (Months)	Disease/study domains
1.	Cattamanchi et al., 2021 (26)	Digital adherence technology for tuberculosis treatment supervision: A stepped-wedge cluster-randomized trial in Uganda	Uganda	Facility	1,913	8	Tuberculosis
2.	Ridout et al., 2023 (27)	CRADLE-5: a stepped-wedge type 2 hybrid implementation effectiveness cluster randomised controlled trial to evaluate the real-world scale-up of the CRADLE Vital Signs Alert intervention into routine maternity care in Sierra Leone—study protocol	Sierra Leone	Facility	160	12	Maternal and Child Health
3.	Tiono et al., 2024 (28)	Efficacy of Olyset Duo, a bednet containing pyriproxyfen and permethrin, versus a permethrin-only net against clinical malaria in an area with highly pyrethroid-resistant vectors in rural Burkina Faso: a cluster-randomised controlled trial	Burkina Faso	Community	4,137	12	Malaria
4.	Giusto et al., 2017 (29)	Associations Between Fathers’ and Sons’ Sexual Risk in Rural Kenya: The Potential for Intergenerational Transmission	Kenya	Community	79	0	Sexual and Reproductive Health
5.	Betrán et al., 2018 (30)	Provision of medical supply kits to improve quality of antenatal care in Mozambique: a stepped-wedge cluster randomised trial	Mozambique	Facility	218,277	23	Maternal and Child Health
6.	Auld et al., 2020 (31)	Effect of tuberculosis screening and retention interventions on early antiretroviral therapy mortality in Botswana: a stepped-wedge cluster randomized trial	Botswana	Facility	8,980	48	HIV/AIDS
7.	Cornelia van Tetering et al., 2021 (32)	Evaluating the Instructional Design and Effect on Knowledge, Teamwork, and Skills of Technology-Enhanced Simulation-Based Training in Obstetrics in Uganda: Stepped-Wedge Cluster Randomized Trial	Uganda	Facility	6,398	24	Maternal and Child Health
8.	Cockcroft et al., 2022 (33)	Universal home visits improve male knowledge and attitudes about maternal and child health in Bauchi State, Nigeria: Secondary outcome analysis of a stepped wedge cluster randomised controlled trial	Nigeria	Facility	6,931	36	Maternal and Child Health
9.	Cisse et al., 2016 (34)	Effectiveness of Seasonal Malaria Chemoprevention in Children under Ten Years of Age in Senegal: A Stepped-Wedge Cluster-Randomised Trial	Senegal	Facility	54	36	Malaria
10.	Davey et al., 2015 (35)	Re-analysis of health and educational impacts of a school-based deworming programme in western Kenya: a statistical replication of a cluster quasi-randomized stepped-wedge trial	Kenya	Community	75	36	General health
11.	Boeke et al., 2020 (36)	Universal test and treat in relation to HIV disease progression: results from a stepped-wedge trial in Eswatini	Eswatini	Facility	3,176	36	HIV/AIDS
12.	Agutu et al., 2022 (37)	Predictors of testing history and new HIV diagnosis among adult outpatients seeking care for symptoms of acute HIV infection in coastal Kenya: a cross-sectional analysis of intervention participants in a stepped-wedge HIV testing trial	Kenya	Facility	1,500	36	HIV/AIDS
13.	Larsen et al., 2015 (38)	Population-Wide Malaria Testing and Treatment with Rapid Diagnostic Tests and Artemether-Lumefantrine in Southern Zambia: A community Randomized Step-Wedge Control Trial Design	Zambia	Facility	85,000	12	Malaria
14.	Meya et al., 2019 (39)	Reflexive Laboratory-Based Cryptococcal Antigen Screening and Preemptive Fluconazole Therapy for Cryptococcal Antigenemia in HIV-Infected Individuals with CD4,100 Cells/mL: A Stepped-Wedge, Cluster-Randomized Trial	Uganda	Facility	3,388	30	HIV/AIDS
15.	Thomas et al., 2022 (40)	A pragmatic approach to identifying implementation barriers and facilitators for a novel pre-exposure prophylaxis (PrEP) delivery model at public facilities in urban Uganda	Uganda	Facility	388	12	HIV/AIDS
16.	Sanders et al., 2021 (41)	Effect of an opt-out point-of-care HIV-1 nucleic acid testing intervention to detect acute and prevalent HIV infection in symptomatic adult outpatients and reduce HIV transmission in Kenya: a randomized controlled trial	Kenya	Facility	19,464	36	HIV/AIDS
17.	Abrams et al., 2019 (42)	Impact of universal antiretroviral therapy for pregnant and postpartum women on antiretroviral therapy uptake and retention	Eswatini	Facility	2,347	24	HIV/AIDS
18.	Irungu et al., 2021 (43)	Integration of pre-exposure prophylaxis services into public HIV care clinics in Kenya: a pragmatic stepped-wedge randomised trial	Kenya	Facility	4,898	8	HIV/AIDS
19.	Wroe et al., 2021 (44)	A household-based community health worker programme for non-communicable disease, malnutrition, tuberculosis, HIV and maternal health: a stepped-wedge cluster randomized controlled trial in Neno District, Malawi	Malawi	Facility	250	60	General health
20.	Sacks et al., 2020 (45)	Impact of Routine Point-of-Care Versus Laboratory Testing for Early Infant Diagnosis of HIV: Results From a Multicountry Stepped-Wedge Cluster-Randomized Controlled Trial	Kenya and Zimbabwe	Facility	9,539	24	HIV/AIDS
21.	Wanyenze et al., 2023 (46)	Efficacy of midwife-led role orientation of birth companions on maternal satisfaction and birth outcomes: a randomized control trial in Uganda	Uganda	Facility	580	15	Maternal and Child Health
22.	Wanyenze et al., 2023 (47)	Effect of Midwife-Provided Orientation of Birth Companions on Maternal Anxiety and Coping during Labor: A Stepped Wedge Cluster Randomized Control Trial in Eastern Uganda	Uganda	Facility	475	12	Maternal and Child Health
23.	Puffer et al., 2016 (48)	A church-based intervention for families to promote mental health and prevent HIV among adolescents in rural Kenya: Results of a randomized trial	Kenya	Community	327	12	HIV/AIDS
24.	Mosha et al., 2005 (49)	Evaluation of the effectiveness of a clean delivery kit intervention in preventing cord infection and puerperal sepsis among neonates and their mothers in rural Mwanza Region, Tanzania	Tanzania	Facility	3,262	0	Maternal and Child Health
25.	Rudolf et al., 2021 (50)	Increasing smear positive tuberculosis detection using a clinical score—A stepped wedge multicenter trial from Africa	Bissau, Guinea-Bissau, and Gondar, Ethiopia.	Facility	3,571	12	Tuberculosis
26.	Jobson et al., 2021 (52)	Indicator-focused technical assistance in South Africa's HIV programme: A stepped-wedge evaluation	South Africa	Facility	17	10	HIV/TB
27.	Musinguzi et al., 2020 (51)	Cardiovascular risk factor mapping and distribution among adults in Mukono and Buikwe districts in Uganda: small area analysis	Uganda	Community	4,372	2	Cardiovascular Diseases
28.	Amanyire et al., 2016 (53)	Effects of a multicomponent intervention to streamline initiation of antiretroviral therapy in Africa: a stepped-wedge cluster-randomised trial	Uganda	Facility	12,024	28	HIV/AIDS
29.	Chirambo et al., 2021 (54)	Effectiveness of Smartphone-Based Community Case Management on the Urgent Referral, Reconsultation, and Hospitalization of Children Aged Under 5 Years in Malawi: Cluster-Randomized, Stepped-Wedge Trial	Malawi	Facility	6,965	3	General health
30.	Yapa et al., 2020 (55)	Infant feeding knowledge and practice vary by maternal HIV status: a nested cohort study in rural South Africa	South Africa	Facility	1,693	18	HIV/AIDS
31.	Yapa et al., 2020 (56)	The impact of continuous quality improvement on coverage of antenatal HIV care tests in rural South Africa: Results of a stepped-wedge cluster-randomised controlled implementation trial	South Africa	Facility	13,212	19	HIV/AIDS
32.	Graham et al., 2019 (57)	Oxygen systems to improve clinical care and outcomes for children and neonates: A stepped-wedge cluster-randomised trial in Nigeria	Nigeria	Facility	24,117	24	Neonatal health
33.	Graham et al., 2021 (58)	Oxygen systems and quality of care for children with pneumonia, malaria and diarrhoea: Analysis of a stepped-wedge trial in Nigeria	Nigeria	Facility	7,141	46	Pneumonia, Malaria and Diarrhoea
34.	Okinyi et al., 2022 (59)	“I Have Actually not Lost any Adolescent Since I Started Engaging Them one on one:” Training Satisfaction and Subsequent Practice among Health Providers Participating in a Standardized Patient Actor Training to Improve Adolescent Engagement in HIV Care	Kenya	Facility	95	60	HIV/AIDS
35.	Anger et al., (60)	The effectiveness and safety of introducing condom-catheter uterine balloon tamponade for postpartum hemorrhage at secondary level hospitals in Uganda, Egypt and Senegal: a stepped wedge, cluster-randomized trial	Senegal, Egypt and Uganda	Facility	2,394	18	Maternal and Child Health
36.	Ndiaye et al., 2016 (61)	Correction: Safety of Seasonal Malaria Chemoprevention (SMC) with Sulfadoxine- Pyrimethamine plus Amodiaquine when Delivered to Children under 10 Years of Age by District Health Services in Senegal: Results from a Stepped-Wedge Cluster Randomized Trial	Senegal	Facility	780,000	36	Malaria
37.	Sturt et al., 2023 (62)	Safety and upscaling of remote consulting for long-term conditions in primary health care in Nigeria and Tanzania (REaCH trials): stepped-wedge trials of training, mobile data allowance, and implementation	Nigeria and Tanzania	Facility	12,022	12	General health
38.	Ndirangu et al., 2022 (63)	We have goals but [it is difficult]’. Barriers to antiretroviral therapy adherence among women using alcohol and other drugs living with HIV in South Africa	South Africa	Facility	480	6	HIV/AIDS
39.	Pfeiffer et al., 2021 (64)	Stepped-Wedge Cluster Randomized Controlled Trial to Promote Option B+ Retention in Central Mozambique	Mozambique	Facility	761	12	HIV/AIDS
40.	Steinert et al., 2021 (65)	The Impact of Immediate Initiation of Antiretroviral Therapy on Patients’ Healthcare Expenditures: A Stepped-Wedge Randomized Trial in Eswatini	Eswatini	Facility	2,261	36	HIV/AIDS
41.	Steinert et al., 2020 (66)	A stepped-wedge randomised trial on the impact of early ART initiation on HIV-patients’ economic outcomes in Eswatini	Eswatini	Facility	3,019	36	HIV/AIDS
42.	Rewley et al., 2020 (67)	Evaluating spillover of HIV knowledge from study participants to their network members in a stepped-wedge behavioural intervention in Tanzania	Tanzania	Facility	1,372	48	HIV/AIDS
43.	Åhsberg et al., 2023 (68)	Point-of-Care Urine Lipoarabinomannan Testing to Guide Tuberculosis Treatment Among Severely Ill Inpatients with Human Immunodeficiency Virus in Real-World Practice: A Multicenter Stepped Wedge Cluster- Randomized Trial from Ghana	Ghana	Facility	422	36	Tuberculosis
44.	Uwamariya et al., 2021 (69)	Safety and effectiveness of a non-electric infant warmer for hypothermia in Rwanda: A cluster-randomized stepped-wedge trial	Rwanda	Facility	3,179	8	Neonatal health
45.	Sabourin et al., 2022 (70)	Evaluation of a training intervention to improve cancer care in Zimbabwe: Strategies to Improve Kaposi Sarcoma Outcomes (SIKO), a prospective community-based stepped-wedge cluster randomized trial	Zimbabwe	Facility	1,102	35	HIV/AIDS
46.	Mugwanya et al., 2023 (71)	Patterns of PrEP continuation and coverage in the first year of use: a latent class analysis of a programmatic PrEP trial in Kenya	Kenya	Facility	4,898	48	HIV/AIDS
47.	Omer et al., 2021 (72)	Impact of universal home visits on child health in Bauchi State, Nigeria: a stepped wedge cluster randomised controlled trial	Nigeria	Community	6,905	0	Maternal and Child Health
48.	Farrant et al., 2022 (73)	Impact of COVID-19 primary healthcare service restrictions on patients with chronic obstructive pulmonary disease in Cape Town, South Africa	South Africa	Facility	49	11	COPD
49.	Ketema et al., 2020 (74)	Evaluating the integration of tuberculosis screening and contact investigation in tuberculosis clinics in Ethiopia: A mixed method study	Ethiopia	Facility	181,455	16	Tuberculosis
50.	Powell et al., 2023 (75)	HIV matters when diagnosing TB in young children: an ancillary analysis in children enrolled in the INPUT stepped wedge cluster randomized study	Cameroon, Kenya	Facility	157	22	Tuberculosis
51.	Multerer et al., 2021 (76)	Estimating intervention effectiveness in trials of malaria interventions with contamination	Kenya	Community	24,879	24	Malaria
52.	Senderowicz et al., 2022 (77)	The effect of a postpartum intrauterine device programme on choice of contraceptive method in Tanzania: a secondary analysis of a cluster-randomized trial	Tanzania	Facility	10,078	12	Sexual and Reproductive Health
53.	Kumbani et al., (78)	A peer group intervention implemented by community volunteers increased HIV prevention knowledge	Malawi	Facility	1,008	30	HIV/AIDS
54.	Yan et al., 2017 (79)	Hypertension management in rural primary care facilities in Zambia: a mixed-methods study	Zambia	Facility	26,363	60	General health
55.	Gichane et al., 2020 (80)	Implementation science outcomes of a gender-focused HIV and alcohol risk reduction intervention in usual-care settings in South Africa	South Africa	Facility	480	48	HIV/AIDS
56.	Fawzi et al., 2019 (81)	Agents of change among people living with HIV and their social networks: stepped-wedge randomised controlled trial of the NAMWEZA intervention in Dar es Salaam, Tanzania	Tanzania	Facility	652	48	HIV/AIDS
57.	Maheu-Giroux et al., 2013 (82)	Do malaria vector control measures impact disease-related behaviour and knowledge? Evidence from a large-scale larviciding intervention in Tanzania	Tanzania	Community	64,537	48	Malaria
58.	Patel et al., 2005 (83)	Supplemental Feeding with Ready-to-Use Therapeutic Food in Malawian Children at Risk of Malnutrition	Malawi	Facility	372	8	Malnutrition
59.	Wyatt et al., 2024 (84)	How PrEP delivery was integrated into public ART clinics in central Uganda: A qualitative analysis of implementation processes	Uganda	Facility	1,381	31	HIV/AIDS
60.	Kiwanuka et al., 2023 (85)	Implementation, feasibility, and acceptability of 99DOTS-based supervision of treatment for drug-susceptible TB in Uganda	Uganda	Facility	462	0	Tuberculosis
61.	van den Broek et al., 2019 (86)	Effects of emergency obstetric care training on maternal and perinatal outcomes: a stepped wedge cluster randomised trial in South Africa	South Africa	Facility	127	25	Maternal and Child Health
62.	Ogbuoji et al., 2019 (87)	Impact of immediate initiation of antiretroviral therapy on HIV patient satisfaction: a stepped wedge cluster-randomized controlled trial	Eswatini	Facility	2,629	36	HIV/AIDS
63.	Kohler et al., (88)	Simulated patient training to improve youth engagement in HIV care in Kenya: A stepped wedge cluster randomized controlled trial	Kenya	Facility	4,595	28	HIV/AIDS
64.	Geldsetzer et al., 2020 (89)	A stepped-wedge randomized trial and qualitative survey of HIV pre-exposure prophylaxis uptake in the Eswatini population	Eswatini	Facility	2,168	36	HIV/AIDS
65.	Naidoo et al., 2017 (90)	Has universal screening with Xpert® MTB/RIF increased the proportion of multidrug-resistant tuberculosis cases diagnosed in a routine operational setting?	South Africa	Community	10,284	19	Tuberculosis
66.	Naidoo et al., 2016 (91)	Comparing Tuberculosis Diagnostic Yield in Smear/Culture and Xpert1 MTB/RIF-Based Algorithms Using a Non-Randomised Stepped-Wedge Design	South Africa	Facility	54,393	36	Tuberculosis
67.	Shete et al., 2023 (92)	Evaluating the impact of cash transfers on tuberculosis (ExaCT TB): a stepped wedge cluster randomised controlled trial	Uganda	Facility	4,288	6	Tuberculosis
68.	Heffron et al., 2022 (93)	PrEP uptake and HIV viral suppression when PrEP is integrated into Ugandan ART clinics for HIV-negative members of HIV-serodifferent couples: A stepped wedge cluster randomized trial	Uganda	Facility	1,381	42	HIV/AIDS
69.	Thompson et al., 2022 (94)	Cost and Cost-Effectiveness of a Digital Adherence Technology for Tuberculosis Treatment Support in Uganda	Uganda	Facility	18	8	Tuberculosis
70.	Ononge et al., 2014 (95)	Haemoglobin status and predictors of anaemia among pregnant women in Mpigi, Uganda	Uganda	Facility	2,436	10	Maternal and Child Health
71.	Ononge et al., 2015 (96)	Effectiveness and safety of misoprostol distributed to antenatal women to prevent postpartum haemorrhage after child-births: a stepped-wedge cluster-randomized trial	Uganda	Facility	2,466	14	Maternal and Child Health
72.	Winani et al., 2007 (97)	Use of A Clean Delivery Kit and Factors Associated with Cord Infection and Puerperal Sepsis in Mwanza, Tanzania	Tanzania	Community	3,262	24	Maternal and Child Health
73.	Dryden-Peterson et al., 2015 (98)	An Augmented SMS Intervention to Improve Access to Antenatal CD4 Testing and ART Initiation in HIV-Infected Pregnant Women: A Cluster Randomized Trial	Botswana	Facility	366	15	Maternal and Child Health
74.	Khan et al., 2020 (99)	Early access to antiretroviral therapy versus standard of care among HIV-positive participants in Eswatini in the public health sector: the MaxART stepped-wedge randomized controlled trial	Eswatini	Facility	3,405	37	HIV/AIDS
75.	Rokicki et al., 2021 (100)	Impact of Solar Light and Electricity on the Quality and Timeliness of Maternity Care: A Stepped-Wedge Cluster-Randomized Trial in Uganda	Uganda	Facility	1,118	12	Maternal and Child Health
76.	Sarrassat et al., 2021 (101)	An Integrated eDiagnosis Approach (IeDA) versus standard IMCI for assessing and managing childhood illness in Burkina Faso: a stepped-wedge cluster randomised trial	Burkina Faso	Facility	2,724	36	Maternal and Child Health
77.	Paddick et al., 2017 (102)	Cognitive stimulation therapy as a sustainable intervention for dementia in sub-Saharan Africa: feasibility and clinical efficacy using a stepped-wedge design	Tanzania	Facility	34	20	General health
78.	Psaki et al., 2022 (103)	What are we learning about HIV testing in informal settlements in KwaZulu-Natal, South Africa? Results from a randomized controlled trial	South Africa	Community	1,528	60	HIV/AIDS
79.	Agizew et al., 2019 (104)	Tuberculosis treatment outcomes among people living with HIV diagnosed using Xpert MTB/RIF versus sputum-smear microscopy in Botswana: a stepped-wedge cluster randomised trial	Botswana	Facility	6,041	24	Tuberculosis
80.	Odeny et al., 2019 (105)	Text messaging for maternal and infant retention in prevention of mother-to-child HIV transmission services: A pragmatic stepped wedge cluster-randomized trial in Kenya	Kenya	Facility	2,515	23	HIV/AIDS
81.	Homan et al., 2016 (106)	The effect of mass mosquito trapping on malaria transmission and disease burden (SolarMal): a stepped-wedge cluster-randomised trial	Kenya	Community	34,041	36	Malaria
82.	Wechsberg et al., 2021 (107)	Outcomes of Implementing in the Real World the Women's Health CoOp Intervention in Cape Town, South Africa	South Africa	Facility	480	36	HIV/AIDS
83.	Mutale et al., 2023 (108)	Protocol-driven primary care and community linkage to reduce all-cause mortality in rural Zambia: a stepped-wedge cluster randomized trial	Zambia	Facility/Community	138,430	48	General health
84.	Killam et al., 2019 (109)	Antiretroviral therapy in antenatal care to increase treatment initiation in HIV-infected pregnant women: a stepped-wedge evaluation	Zambia	Facility	31,536	12	HIV/AIDS
85.	Wichaidit et al., 2019 (110)	Effect of an equipment-behavior change intervention on handwashing behavior among primary school children in Kenya: the Povu Poa school pilot study	Kenya	Community	30	36	Maternal and Child Health

Sixteen (18.8%) were conducted in Uganda (26, 32, 39, 40, 46, 47, 53, 84, 85, 92–96, 100), 14 (15.3%) in Kenya (29, 35, 37, 41, 43, 48, 59, 71, 76, 88, 105, 106, 110), and 11 (12.9%) in South Africa (52, 55, 56, 63, 73, 80, 86, 90, 91, 103, 107). Other countries where SWD has been used includes 7 (8.2%) in Eswatini (36, 42, 65, 66, 87, 99) and Tanzania (49, 67, 77, 81, 82, 97, 102), 4 (4.70%) in Nigeria (33, 57, 58, 72), and Malawi (44, 54, 78, 83), 3 (3.5%) in Botswana (31, 98, 104), 2 (2.4%) in Mozambique (30, 64), Burkina Faso (28, 101), Senegal (34, 61), and Zambia (38, 79), and 1 study each in Ghana (68), Ethiopia (74), Rwanda (69), Sierra Leone (27) and Zimbabwe (70). Five (5.9%) studies were conducted in multi-countries, one study each was conducted across Bissau, Guinea-Bissau, and Gondar, Ethiopia (50); Cameroon and Kenya (75); Kenya and Zimbabwe (45); Nigeria and Tanzania (62); and one study from Senegal, Egypt, and Uganda (60).

### Study duration

The duration of the included studies varied widely, ranging from two months to sixty months. Notably, 4 studies (4.71%) did not report the study duration (29, 49, 72, 85). The most common study length was thirty-six months, observed in 17 (20%) studies (33–37, 41, 61, 65, 66, 68, 87, 89, 91, 101, 106, 107, 110). Additionally, 12 (14.1%) studies were conducted over twelve months (27, 28, 38, 40, 47, 48, 50, 62, 64, 77, 100, 109).

Seven (29.2%) studies each were conducted over twenty-four months (32, 42, 45, 57, 76, 97, 104) and forty-eight months (31, 67, 71, 80–82, 108) durations. Furthermore, 5 (5.9%) studies were conducted over eight months (26, 43, 69, 83, 94), and 4 (4.7%) studies spanned 60 months (44, 59, 79, 103). 2 studies each had the following durations: six months (63, 92), ten months (52, 95), fifteen months (46, 98), eighteen months (55, 60), nineteen months (56, 90), twenty-three months (30, 105), twenty-eight months (53, 88), and thirty months (39, 78). Finally, 1 study was conducted over each of the following periods: two months (51), three months (54), 11 months (73), fourteen months (96), sixteen months (74), twenty months (102), twenty-two months (75), twenty-five months (86), thirty one months (84), thirty-five months (70), thirty-seven months (99), forty-two months (93), and forty-six months (58).

### Disease domains

**Figure 3:**
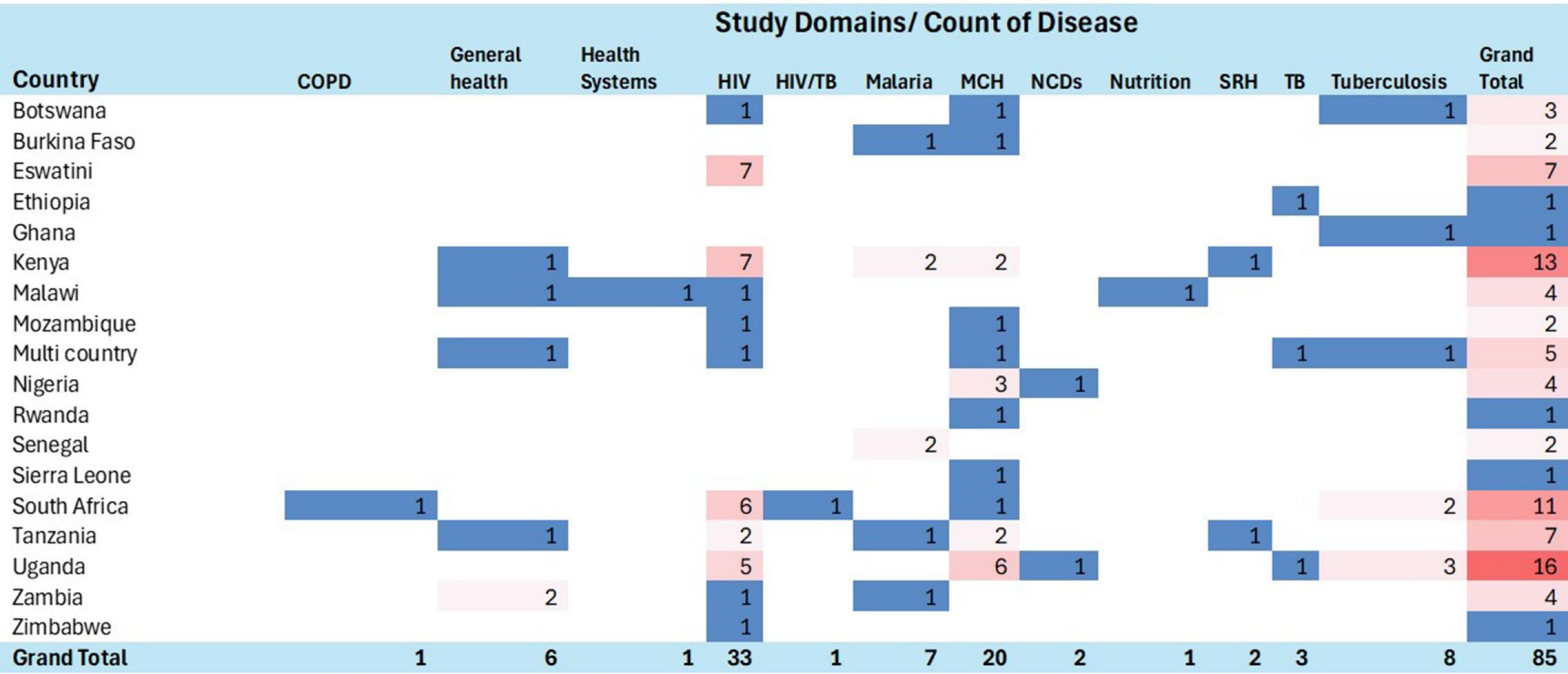
Heatmap showing distribution of stepped-wedge studies by country and health domain. This heatmap presents the distribution of included studies across sub-Saharan African countries and major health domains (e.g., HIV, TB, maternal and child health, malaria, NCDs). Darker shading indicates a higher number of studies within each country–domain combination. The figure highlights the concentration of stepped-wedge studies in Uganda, Kenya, and South Africa, with HIV and maternal and child health being the most frequently studied domains.

Figure 3 shows the distribution of SWDs by country and health domains in SSA. A significant proportion of the studies focused on HIV/AIDS, with thirty-four (40%) studies falling within this domain (31, 36, 37, 39–43, 45, 48, 53, 55, 57, 59, 63–67, 70, 71, 78, 80, 81, 84, 87–89, 93, 99, 103, 105, 107, 109). Seventeen (20%) studies addressed maternal and child health (27, 30, 32, 33, 46, 47, 49, 60, 72, 86, 95–98, 100, 101, 110), while eleven (12.9%) studies focused on tuberculosis (26, 50, 68, 74, 75, 85, 90–92, 94, 104). Malaria was the subject of seven (8.2%) studies (28, 34, 37, 61, 76, 82, 106), and another seven studies (8.2%) were conducted in the general health domain (35, 44, 54, 62, 79, 102, 108).

Two (2.4%) studies each were carried out in the domains of neonatal health (57, 69), and sexual and reproductive health (29, 77). Additionally, one (1.2%) study was conducted in the following domains: cardiovascular health (51), respiratory health (58), and malnutrition (83). Moreover, two (2.4%) studies covered multiple domains, specifically HIV/TB (52) and pneumonia/malaria/diarrhea (58).

Table 2 provides a comprehensive overview of the characteristics of the studies included in the analysis. It details key elements such as the first author and publication year, the definitions of the clusters used, the nature of the interventions implemented, and the randomization process. The table also includes information on the rationale for choosing a stepped-wedge design, the number of steps and clusters involved, the time between steps, sample size calculations, and the primary outcomes of each study. This structured presentation allows for a clear comparison of study designs and outcomes across different interventions and settings.

**Table 2:** Characteristics of included studies.

S/No	First author/year	Cluster definitions	Nature of interventions	Randomization	Reasons for SWD	Number of steps	Number of Clusters	Time between steps	Sample size calculation	Primary outcome
1.	Cattamanchi et al., 2021 (26)	18 health facilities that include 5 regional referral hospitals, 10 general hospitals, and 3 district health centers.	The intervention is a 99DOTS-based TB treatment supervision strategy. 99DOTS involves patients calling toll-free phone numbers hidden underneath pills in blister packs to self-report medication dosing.	Health facilities were first assigned randomly to 1 of 6 blocks of 3 health facilities. The order in which each block switched from routine care to the intervention was assigned randomly using a simple, unrestricted 2-stage process.	The stepped-wedge design increased acceptability among the participating health facilities and feasibility of training sites on the intervention.	6	18	6 months	With 18 clusters and an anticipated harmonic mean of 15 eligible patients per month at each health facility, the trial had 89% power to detect a 10% increase in the treatment success proportion. The calculation assumed a pre-implementation treatment success rate of 51% and an intraclass correlation coefficient of 0.001.	The primary outcome was the proportion of patients with treatment success, defined as having a treatment outcome of cured or treatment completed recorded in the TB treatment register.
2.	Ridout et al., 2023 (27)	Eight rural districts in Sierra Leone	The CRADLE Vital Signs Alert intervention involves using a hand-held, semi-automated device that measures blood pressure and pulse with an inbuilt traffic-light early warning system, accompanied by a focused training package for healthcare providers.	This is a stepped-wedge cluster-randomized controlled trial. Districts were randomized to receive the intervention at different time points.	The stepped wedge design was chosen to allow all districts to eventually receive the intervention while evaluating its effectiveness over time. It also helps in understanding how the intervention can be embedded sustainably into routine clinical pathways.	2	8	6 weeks	Sample size estimation was based on an expected 1000 deliveries per district per six weeks, across eight districts, observed for 12 months. The calculations accounted for within-period intra-cluster correlations and cluster autocorrelation coefficients. The study aimed for a power close to 100%, even with an extremely high intra-cluster correlation of 0.5.	The primary outcome was the composite rate of maternal mortality, major maternal morbidity, and fetal losses. This included maternal death, eclampsia, emergency hysterectomy, and stillbirth.
3.	Tiono et al., 2024 (28)	Groups of one to four neighboring villages in rural Burkina Faso.	The intervention involved replacing standard long-lasting insecticidal nets (LLINs) with LLINs treated with permethrin plus pyriproxyfen (PPF). PPF is an insect growth regulator combined with permethrin, a pyrethroid insecticide, aimed at providing greater protection against clinical malaria.	At the start of the malaria transmission season in June 2014, five clusters were randomly selected to receive PPF-treated LLINs, while the remaining 35 clusters received standard LLINs. Subsequently, five clusters were randomly selected each month from July to September 2014 to replace their standard LLINs with PPF-treated LLINs, ensuring that by the end of 2014, both study groups had an equal number of clusters. This random selection was done using Stata version 10.	The stepped-wedge design was chosen to facilitate a phased implementation of the intervention, mimicking the type of deployment used by net distribution programs. This approach allowed all clusters to eventually receive the intervention while assessing its effectiveness over time.	4	40	One month	The study was designed to detect a protective efficacy of 25% with PPF-treated LLINs compared to standard LLINs, with 90% power and a 5% significance level. The power calculation assumed an incidence of clinical episodes of malaria of 1.5 episodes per child each year and a coefficient of variation of 0.5. The study was also 80% powered to detect a 33% reduction in the entomological inoculation rate (EIR) at a 5% significance level.	The primary endpoints were the incidence of clinical episodes of malaria among cohort children presenting at health facilities and the entomological inoculation rate (EIR).
4.	Giusto et al., 2017 (29)	Churches, which include diverse denominations such as Protestant, indigenous, and Catholic	The intervention is family- and church-based, aimed at strengthening family relationships and preventing HIV risk behavior among adolescents.	A total of 56 churches were identified, and four were randomly selected to participate through lotteries conducted by community leaders. This included three Protestant churches and one indigenous church.	The study does not explicitly state the reasons for using the stepped wedge design (SWD), but it implies a phased implementation of the intervention to allow for systematic assessment and control of intervention timing across different clusters.	–	4	–	The sample size included 79 dyads of male caregivers (*n*=61) and male youths (*n*=79) from a larger sample of 211 female and male caregivers and 237 youths from 124 households.	The primary outcome focuses on the associations between fathers’ and sons’ sex-related beliefs and sexual behaviors, moderated by parenting characteristics and the quality of father-son relationships.
5.	Betrán et al., 2018 (30)	Clusters were antenatal care clinics in health facilities selected purposely by the Ministry of Health according to its programmatic activities and priorities and with geographical representation of the three regions of Mozambique (north, centre, and south).	The intervention was multifaceted with four components: – Antenatal care kits (boxes containing supplies necessary – to carry out a specific number of antenatal care visits) – A cupboard to organise and store the supplies locked in the – antenatal care room – A tracking sheet to monitor the kits’ – stock levels; and – - A training session for the health-care providers at the beginning of the intervention.	Clinics were assigned to one of ten start dates by the study statistician via a computer-generated list of random numbers. Concealment of the intervention start date was not possible for logistic reasons, because of the involvement of the Ministry of Health in the preparatory activities required to launch the intervention at each clinic.		11	10	2 months	We assumed, conservatively, a baseline frequency of 30% for each selected health practice, and an increase to 60% with implementation of the intervention. For a 0·05 alpha level, 80% power, and an intra-cluster correlation coefficient of 0·05, six clusters were needed.	
6.	Auld et al., 2020 (31)	HIV care and treatment clinic	The ICF and active tracing interventions: – Additional human – resources (study nurses) to support implementation, – Additional training for clinic and laboratory personnel, – Use of checklists and job aids to standardize implementation, and – Regular supervisory visits to track adherence – to ICF and tracing checklists	The selected 22 clusters received TB diagnostic services from 13 laboratories	A stepped wedge rather than parallel group design was chosen because the Xpert, ICF, and retention package was expected to be beneficial for patients and the trial was part of a national rollout.	3	21	1 month	We used the approach of Moulton et al., suitable for stepped-wedge trial designs, to estimate required sample sizes to meet the primary study objective comparing 6-month ART mortality rates between SOC and EC+X phases. A between-cluster coefficient of variation of 0.2 was used based on review of the literature of similar stepped-wedge trials. To provide > 80% power to detect a ≥ 40% reduction in all-cause 6-month ART mortality between the two groups, assuming SOC mortality was ≥ 10/100 person-years, a 24-month SOC phase enrollment period (*N* = 12,144) and an 18-month EC+X phase enrollment period (*N* = 6,348) was chosen.	The primary objective reported here is the non-randomized comparison of all-cause 6-month ART mortality among adult ART enrollees (≥12 years old) between the SOC and EC+X phases
7.	Cornelia van Tetering et al., 2021 (32)	Senior House Officers (SHOs) working in the medium-to-high–risk maternity ward of Mulago Hospital, Kampala, Uganda. Seven clusters of first-year, second-year, and third-year SHOs were created.	The intervention involved a one-day (8-hour) simulation-based acute obstetric training focusing on medical technical skills and teamwork/crew resource management. Training was cascaded from master trainers to local facilitators and then to SHOs. Scenarios included postpartum hemorrhage, eclampsia, ventouse delivery followed by resuscitation of the newborn, and breech delivery, among others.	Seven clusters of SHOs were randomly created by a scheduler. All clusters started in a control condition and received the training at consecutive time points scheduled seven weeks apart. The order of the switch per cluster was randomized by a computer.	The stepped-wedge design was chosen to allow for phased implementation of the training while evaluating its impact over time.	7	7	7 weeks	A sample size calculation was performed based on the primary outcome of the study (the combined mortality proportion including maternal and neonatal mortality ratios). To show a reduction in combined mortality proportion of 20% with an *α* of.05 and a power of 80%, a total of 6,398 deliveries were needed for a standard randomized clinical trial design. The design effect was calculated assuming an intracluster correlation of 0.05, 7 clusters, and a cluster size of 3,343 deliveries per year, resulting in 2,367 deliveries per cluster period. This resulted in a minimum duration of 5 weeks for each cluster period. The duration of each step was set at 7 weeks.	Primary outcomes included knowledge improvement, teamwork (measured by the Clinical Teamwork Scale), and medical technical skills.
8.	Cockcroft et al., 2022 (33)	8 Wards	The intervention consisted of universal home visits by trained female and male home visitors to all pregnant women and their spouses. The visits aimed to discuss local risk factors for maternal and child health, including danger signs during pregnancy and childbirth, heavy work during pregnancy, domestic violence, and spousal discussion about pregnancy and childbirth. The intervention also covered early childhood health topics like prevention and management of diarrhoea and routine immunisation.	The eight wards in Toro LGA were randomly allocated into four waves, with two wards per wave. The implementation of the intervention began at yearly intervals, with each wave starting the home visits at different times.	The stepped wedge design was chosen to ensure all wards could eventually receive the intervention, to control for time effects due to evolving community programs, and to facilitate the logistics of implementing the intervention by a single study team.	4	8	1 year	We calculated the trial sample size based on maternal outcomes. Since male outcomes (knowledge and attitudes) were more common than the measured maternal outcomes, the trial had more power to detect significant differences in these outcomes.	The primary outcomes among male spouses included knowledge and attitudes related to maternal and child health, such as: – Knowledge of danger signs during pregnancy and childbirth. – Beliefs about heavy work during pregnancy. – Discussion with their spouse about pregnancy and childbirth. – Knowledge about the causes and management of childhood diarrhoea. – Views about childhood immunization.
9.	Cisse et al., 2016 (34)	54 health posts serving three health districts	The intervention was Seasonal Malaria Chemoprevention (SMC) with sulfadoxine-pyrimethamine (SP) plus amodiaquine (AQ) administered monthly during the malaria transmission season to children under ten years of age.	The 54 health posts were randomized to receive the intervention in a stepped-wedge manner over three years. In the first year, 9 health posts started SMC, 18 in the second year, and another 18 in the third year, with 9 health posts remaining as controls.	The stepped-wedge design was chosen to gradually introduce the intervention for logistic reasons, ensure all clusters eventually received the intervention, and adjust for time effects.	10	54	1 year	Sample size calculations were based on the assumption of a mortality rate of 20 per 1,000 in children aged 3–59 months and SMC effectiveness against malaria of 70%. The study had varying power to detect reductions in mortality depending on the scenario and assumed coefficients of variation.	The primary outcomes were all-cause mortality, the incidence of RDT-confirmed malaria, and known drug-related adverse events.
10.	Davey et al., 2015 (35)	75 primary schools	The intervention included regular public lectures, wall charts, and training for one teacher per school by staff from the Kenya Ministry of Health Division of Vector-Borne Diseases (DVBD) to deliver health messages. Additionally, bi-annual albendazole treatment was provided in schools with a high prevalence of geohelminth infection, and annual mass treatment with praziquantel was given in schools with a high prevalence of schistosomiasis.	The schools were systematically allocated or 'quasi-randomized' into three groups using an Excel spreadsheet by the study authors. Group 1 received the intervention in both years, Group 2 in the second year only, and Group 3 did not receive the intervention during the study period.	The reasons for using a stepped wedge design included ensuring that all schools could benefit from the intervention, adjusting for time effects given evolving programs, and enabling effective implementation by a single study team.	2	3	-	-	The primary outcomes were school attendance and educational attainment (examination performance). Secondary outcomes included worm infection rates and anthropometric measures (weight-for-age and height-for-age).
11.	Boeke et al., 2020 (36)	14 government-managed health facilities in Eswatini's Hhohho Region	Universal test and treatment (UTT), where all people living with HIV (PLHIV) are eligible for antiretroviral therapy (ART) regardless of disease severity.	Health facilities were grouped and transitioned two at a time from the control (standard of care) to the intervention stage in each 4-month step interval.	To systematically roll out the UTT intervention across all facilities over time, allowing each facility to serve as its own control while controlling for time-related confounders and logistical implementation of the intervention.	The study involved multiple steps, transitioning pairs of facilities every 4 months. The exact number of steps isn't explicitly mentioned.	14	4 months	NA	Time to HIV disease progression, defined as the time from study enrolment to the first incident of any one of the following events: CD4 count falling below 200 cells/µL or the value at study enrolment, weight loss of > 10%, a drop of BMI to < 18.5, development of tuberculosis (TB), or HIV-related death.
12.	Agutu et al., 2022 (37)	Six primary care facilities in Mombasa and Kilifi counties	Targeted HIV-1 nucleic acid (NA) testing intervention compared with standard provider-initiated testing using rapid antibody tests. Participants underwent a questionnaire and NA testing, followed by rapid tests if NA-positive.	The study utilized a modified stepped-wedge design, recruiting patients presenting at the six facilities over the intervention period. Participants were enrolled consecutively, with no explicit mention of randomization procedures within clusters.	To systematically roll out the intervention to all clusters over time, ensuring all facilities eventually receive the intervention while controlling for time-related confounders and improving the logistical feasibility of the intervention implementation.	NA	6	NA	NA	New HIV diagnosis among adult outpatients presenting with symptoms of acute HIV infection.
13.	Larsen et al., 2015 (38)	Level of health facility catchment areas (HFCAs) - involved 18 contiguous randomization groups, each containing 2–3 HFCAs, in Southern Province, Zambia	Mass test and treat (MTAT) for malaria using rapid diagnostic tests (RDTs) and artemether-lumefantrine (AL) to clear malaria infections.	46 HFCAs were organized into 18 contiguous randomization groups. Eight groups were randomly selected to begin the MTAT intervention in 2012, while the remaining 10 groups served as a contemporaneous control group. The randomization was done using satellite imagery from Google Earth to create the randomization groupings, which were then confirmed by District Health Teams​	This design allowed for a phased implementation while providing a rigorous evaluation of the intervention's effectiveness​	3	18	Two months	The sample size was calculated to measure a 50% reduction in malaria parasite prevalence between intervention and control groups. The study aimed to include 3,000 children under 5 years of age in 2,100 households over two survey rounds. This sample size was based on an assumed 10% malaria parasite prevalence at baseline with a design effect of two	The primary outcome was malaria parasite prevalence among children aged 1–59 months. Secondary outcomes included confirmed and total outpatient malaria case incidence as reported in the health management information system (HMIS)​
14.	Meya et al., 2019 (39)	Outpatient HIV clinic level—involved 17 HIV clinics in Uganda, including 11 urban and 6 rural sites	reflexive laboratory-based cryptococcal antigen (CrAg) screening and preemptive fluconazole therapy for asymptomatic CrAg-positive individuals. The intervention aimed to reduce the incidence of cryptococcal meningitis and improve survival among HIV-infected individuals with CD4 counts <100 cells/mL.	The clinics were randomized to receive the intervention in a stepped-wedge manner. The Infectious Diseases Institute Clinic in Kampala initiated the intervention, followed by other clinics at two-month intervals. Each cluster had an initial observational phase followed by an interventional phase with CrAg screening and preemptive fluconazole therapy​	This design allowed for staggered training of clinical staff, laboratory personnel, and pharmacists while enabling a rigorous evaluation of the intervention's impact	Multiple steps—with each clinic transitioning from the observational to the interventional phase every two months	17	Two months	The trial aimed to include 2,190 participants with a CD4 count <100 cells/mL. The study was powered to detect a hazard ratio of ≥1.15 for 6-month survival with 80% power and a one-sided alpha level of 0.05	The primary outcome was 6-month survival among participants with CD4 counts <100 cells/mL. Secondary outcomes included the incidence of symptomatic central nervous system disease, all-cause early fluconazole discontinuation, and serious adverse events.
15.	Thomas et al., 2022 (40)	12 public ART clinics in Kampala and Wakiso, Uganda.	The intervention involved integrating PrEP delivery into existing ART programming for HIV sero-different couples. PrEP was offered to HIV-negative members of sero-different couples alongside ART for HIV-positive partners. Providers received a 2-day training on PrEP delivery, and the program included routine technical assistance visits to support implementation.	Facilities were randomized (in groups of four) to one of three steps for beginning intervention delivery. The first group started delivery, followed by the second group six months later, and the third group 12 months after the first group. Randomization was done to ensure phased implementation.	The stepped-wedge design was chosen to accommodate logistical constraints and allow for phased implementation of the intervention. This approach also facilitated the collection of data over time to assess the impact of the intervention on HIV viral suppression among partners living with HIV.	3	12	6 months	NA	The primary outcome was HIV viral suppression among partners living with HIV. Secondary outcomes included PrEP initiation, PrEP refills, ART initiation, and HIV viral load data abstracted from clinic records.
16.	Sanders et al., 2021 (41)	Four public and two private health facilities	The intervention was an opt-out point-of-care HIV-1 nucleic acid testing (NAAT) to detect acute and prevalent HIV infection in symptomatic adult outpatients.	The study used a modified stepped-wedge trial design. Health facilities transitioned sequentially to the intervention phase after an initial observation period.	The stepped wedge design was chosen because the intervention was expected to do more good than harm, and it was not practical to deliver the intervention simultaneously to all participants due to resource constraints.	Multiple steps—Each health facility transitioning from the control to the intervention phase in phases that lasted six months per site, except the first site, which had only three months of observation.	6	6 months, except for the first site, which had only three months of observation.	NA	The primary outcome was the number of new HIV diagnoses in each study period, combining chronic HIV and acute HIV diagnoses.
17.	Abrams et al., 2019 (42)	12 health facilities in eSwatini, selected from two geographically contiguous regions, Manzini and Lubombo	The intervention involved transitioning from CD4+ guided ART eligibility (Option A) to universal lifelong ART for all HIV-positive pregnant and breastfeeding women (Option B+). Option A involved ART eligibility based on immunologic and clinical status, while Option B+ provided ART to all pregnant and breastfeeding women regardless of CD4+ cell count or WHO stage.	A staggered rollout was implemented, with one facility transitioning from Option A to Option B+ approximately every four weeks. Two facilities continued Option A services as control sites.	-	3	12	Approximately four weeks between the transition of each facility from Option A to Option B+.	NA	Maternal retention in care, defined as the proportion of women attending clinic within 56 days of delivery (antenatal retention) and clinic attendance within 84 days of 6-months postpartum (postnatal retention).
18.	Irungu et al., 2021 (43)	Level of public HIV care clinics in Kenya. Each clinic represents a cluster in the study. The study involved 25 high-volume public HIV care clinics	Integration of pre-exposure prophylaxis (PrEP) services into public HIV care clinics. This included PrEP training for health-care workers and provision of ongoing PrEP technical assistance to HIV clinics. PrEP was provided by Ministry of Health staff without additional financial support​.	Clinics were stratified by region and then randomly assigned to the order in which they would start receiving the intervention. The randomization was done at stakeholder events using numbered opaque balls picked from a bag by a representative from each health facility. There was no masking in this study	The stepped wedge design was chosen because the intervention could not be implemented simultaneously in all clinics. This design allowed for a staggered approach to implementation, facilitating logistical management and enabling all clinics to eventually receive the intervention while also providing a rigorous evaluation of the intervention's impact	The intervention rollout involved multiple steps, with two to six HIV care clinics crossing over from control to intervention every month until all clinics were implementing the intervention	25	Monthly	The sample size was calculated to have more than 90% power to detect a minimum 10% absolute difference in the number of at-risk HIV-uninfected individuals initiating PrEP after implementing the intervention. The study aimed to have 4,800 individuals in total, with 24 clinics, a baseline period, and two to four clinics implementing at each step​	The primary outcome was the number of people initiating PrEP per clinic per month, comparing the intervention to control periods. Secondary outcomes included PrEP continuation, adherence, incident HIV infections, and fidelity to core components of PrEP delivery.
19.	Wroe et al., 2021 (44)	11 healthcare facilities	The intervention involved expanding an existing HIV and TB-specific community health worker (CHW) program into a polyvalent, household-based model that included non-communicable diseases (NCDs), malnutrition, TB screening, family planning, and antenatal care (ANC)​.	The clusters were randomized using a random number generator. A new cluster received the intervention every three months until all clusters had received it.	The stepped-wedge design was chosen to allow all clusters to eventually receive the intervention while maintaining randomization and to address ethical concerns of withholding an effective intervention.	6	6	3 months	NA	The primary outcomes included HIV default rate, NCD default rate, paediatric malnutrition case finding, TB case finding, family planning uptake, and first trimester ANC enrolment.
20.	Sacks et al., 2020 (45)	18 health facilities in each country, 36 health facilities (Kenya and Zimbabwe)	The intervention compared routine point-of-care (POC) early infant diagnosis (EID) of HIV with standard laboratory-based EID. The POC platforms were implemented at health facilities to provide on-site HIV testing for infants, aiming to improve the timeliness of diagnosis and treatment.	The study was a prospective stepped-wedge cluster-randomized controlled trial. Health facilities were randomly assigned to different steps, indicating when they would receive the POC platform and begin POC EID testing. Each step lasted for four months, with a one-month transition period in between.	The stepped wedge design allowed all participating facilities to eventually receive the intervention while enabling the evaluation of its impact over time. This design was chosen to maximize access to POC EID while containing costs and maintaining operational efficiency.	4	18 health facilities (clusters) in each country, totaling 36 clusters.	One month	The sample size was calculated to detect at least a 50% increase in the proportion of caregivers receiving results by 12 weeks of infant age after the introduction of the POC intervention. The study assumed a design effect of 2 and included 18 clusters in each country.	The primary outcome was the proportion of caregivers receiving EID results by the time the infant was 12 weeks of age. Secondary outcomes included the turnaround time (TAT) from sample collection to result return, the percentage of HIV-infected infants started on antiretroviral therapy (ART) within 60 days of sample collection, and the TAT to ART initiation.
21.	Wanyenze et al., 2023 (46)	Health facility	The intervention involved midwife-provided orientation sessions for birth companions on supportive labor techniques. The content of these sessions included providing emotional and physical support, such as saying calming verbal expressions, using humor, encouraging the woman, supporting her to change positions, giving her drinks and food, massaging, and helping her find a comfortable position for pushing	Randomization was done by generating a random sequence of the four hospitals using a simple random technique. The sequence “2, 4, 1, and 3” guided the order in which each facility crossed over to the intervention period	The stepped-wedge design was chosen due to the anticipated difficulty in simultaneously introducing the intervention to all clusters and for ethical reasons to ensure that no clusters were denied the beneficial intervention. It also helped buffer the effects of heterogeneity between health facilities​	4	4	1 month	The sample size for this trial was calculated based on the primary outcome (incidence of having a spontaneous vaginal delivery). A sample size of 290 participants per period was calculated, with a total of 580 eligible participants recruited for the study​	The primary outcome was the incidence of having a spontaneous vaginal delivery​
22.	Wanyenze et al., 2023 (47)	Facility was labeled as a cluster. The clusters included one district hospital (Bududa), one regional referral hospital in Mbale district, and two Health Centre IVs	The intervention was "midwife-provided orientation of birth companions". The admitting midwife provided an orientation session for the birth companion on supportive labor techniques.	Randomization was performed using a simple random technique. The principal investigator generated a random sequence of the four hospitals using a random sequence generator. The sequence determined which facility crossed over first to the intervention period.	The stepped wedge design was chosen because of the anticipated difficulty in simultaneously introducing the intervention to the different clusters and for ethical purposes to not withhold a beneficial intervention from some clusters.	-	4	-	The sample size was calculated based on the primary outcome (incidence of having a spontaneous vaginal delivery). The baseline rate for spontaneous delivery was 87%. A sample size of 290 participants per period was calculated. Approximately 12,500 women delivered during the study period, with 580 eligible participants recruited for the study.	The primary outcome was the chance of having a spontaneous vaginal delivery. The study also assessed secondary outcomes like the incidence of spontaneous vaginal delivery, length of labor, Apgar score, coping, anxiety, and maternal satisfaction.
23.	Puffer et al., 2016 (48)	124 families (237 adolescents ages 10 to 16; 203 caregivers) from four churches in rural Kenya	A family- and church-based program aimed at improving family relationships, reducing HIV risk, and promoting mental health. The intervention included modules addressing economic, relationship, and HIV-related topics using evidence-based behavioral strategies alongside culturally-grounded content.	A stepped wedge cluster randomized trial was conducted, with four churches randomly assigned to receive the intervention first, second, third, or last.	The stepped wedge design allowed all participants to eventually receive the intervention while enabling the evaluation of its effects over time.	5	4	1 month	-	The primary outcomes measured were family communication, HIV risk knowledge, self-efficacy, and beliefs. Secondary outcomes included parenting, social support, mental health, and adolescent sexual behavior.
24.	Mosha et al., 2005 (49)	Level of dispensaries or health centers providing antenatal care. ​	Providing pregnant mothers with a clean delivery kit during their first antenatal visit, coupled with health education on the principles of the "six cleans" (clean hands, clean perineum, clean delivery surface, clean cord cutting and tying instruments, clean cutting surface).	The cluster implementation schedule was randomized. Clusters were sectioned into ten, with one new cluster starting the intervention every six weeks according to the randomised stepped-wedge design schedule	The stepped wedge design was chosen to allow phased implementation of the intervention, ensuring all clusters eventually received the intervention while enabling a rigorous evaluation of its effectiveness. This design also facilitated logistical management and the staggered rollout of the intervention	10	10	Six weeks	NA	The primary outcomes were the incidence of cord infection and puerperal sepsis among neonates and their mothers. The study evaluated the effectiveness of clean delivery kits in preventing these infections​
25.	Rudolf et al., 2021 (50)	Six health centers	The intervention involved using the Bandim TBscore, a clinical score designed to predict treatment outcomes in tuberculosis (TB) patients and to aid in identifying which healthcare-seeking adults to refer for sputum smear microscopy. The intervention aimed to increase smear-positive TB detection by implementing the TBscore in routine clinical practice.	This study was a stepped wedge cluster-randomized trial, where all health centers started in the control phase and then sequentially transitioned to the intervention phase at random intervals.	The stepped wedge design was chosen to ensure that all participating health centers would eventually implement the intervention. This design allows for the evaluation of the intervention's effectiveness while accounting for temporal trends and resource constraints.	4	6	NA	Sample size was determined using the "steppedwedge" function in Stata. It was estimated that an increase in detection rate from 6% to 10% could be detected with a power of 80% and a significance level of 0.05. With three clusters in each country, 152 patients per time interval (of 19 weeks) and cluster were needed, resulting in a target of 3,648 total inclusions.	The primary outcome was the diagnostic yield for smear-positive TB. Secondary outcomes included successful treatment and the effect on overall 12-month mortality.
26.	Jobson et al., 2021 (52)	Parish level—20 parishes in Mukono and Buikwe districts in Uganda	To improve cardiovascular disease (CVD) risk profiles through health education, screening, and management of modifiable risk factors including hypertension, diabetes, obesity, sedentary behavior, smoking, poor diet, and psychosocial stress​	The parishes were randomly assigned to the order in which they would receive the intervention. The randomization ensured that all parishes eventually received the intervention while allowing for a staggered rollout	This design also accommodated logistical and practical considerations for rolling out the intervention in a resource-constrained setting​	Multiple steps, with each parish transitioning to the intervention phase at different times​	20	NA	A sample of 4,372 adults aged 25–70 years was drawn from 3,689 randomly selected households across 80 villages in the 20 parishes. The sample size was designed to provide adequate power to detect changes in CVD risk factors through the intervention	The primary outcome was the change in the prevalence of CVD risk factors, including hypertension, diabetes, obesity, physical inactivity, smoking, unhealthy diet, and alcohol consumption. The study also aimed to map the distribution of these risk factors to inform targeted interventions
27.	Musinguzi et al., 2020 (51)	Level of primary healthcare and community healthcare centres. The study involved 17 primary and community healthcare centres in the Mopani district of the Limpopo province, South Africa​	Targeted package of technical assistance (TA) for HIV and tuberculosis (TB) treatment and care. The TA package focused on clinical, managerial, and pharmacy services provided by three roving teams over six months​	Facilities were randomized to receive the intervention at different times using random number lists, resulting in a stepped-wedge sequence of support implementation	The stepped wedge design was chosen because it enabled the use of a randomized controlled approach alongside the implementation process. It was also ethical and logistically feasible, ensuring all facilities eventually received the intervention​.	The intervention rollout involved three phases: intensive support, maintenance support, and standard support over several months until all facilities had received the intervention	17	Each facility received two months of intensive support followed by four months of lower-intensity maintenance support	Sample size calculations focused on the proportion of patients screened for TB at ART initiation, patients retained in care at 3- and 6-months post-ART initiation, and TB treatment success rates. The data analysis used routine programme data from health management information systems​	The primary outcomes included the proportion of HIV-infected adults screened for TB at ART initiation, the proportion of adults on ART retained in care at 3 and 6 months, and TB treatment success rates. These outcomes were measured pre- and post-intervention implementation​
28.	Amanyire et al., 2016 (53)	Health facilities providing HIV treatment	Opinion-leader-led training about the evidence regarding clinical and behavioural consequences of waiting to off er ART to recalibrate the balance of risks and benefits of offering ART made by doctors, nurses, clinical officers, and counsellors.	Health facilities (clinics) were the unit of randomisation. Four groups of five clinics were randomly assigned to receive the intervention in one of four steps at intervals of about 6 months.	All components of the intervention were existing (but not fully implemented) elements, such as the use of point-of-care diagnostics in rural settings, the use of data for feedback to clinic sites, and dissemination of clinical and epidemiological information to improve care.	4	5	14 days	On the basis of assumptions that 25% of patients in the control group and 50% of patients in the intervention group would achieve the primary outcome, and with five clinics randomly assigned to the intervention in each of four steps, a harmonic mean of 50 individuals in each clinic during each period, and a coefficient of variation of 0·2, we calculated that the study had 99% power. HIV RNA levels were assessed 1 year after ART eligibility in a random sample of patients from clinics randomised in step 1 (intervention) and step 4 (control) in whom a year of observation in the same treatment condition was possible. With the assumptions of a coefficient of variation of 0·10, 80% of patients achieving HIV RNA suppression in the control group, 92% of patients achieving HIV suppression in the intervention group (a 12% absolute risk difference), and a harmonic mean of 50 patients per clinic, we calculated a power of 77%.	The primary endpoint was ART initiation within 14 days after the first date of clinical eligibility for ART during the study period.
29.	Chirambo et al., 2021 (54)	102 village clinics	The intervention was the use of a smartphone-based e-CCM (electronic community case management) app designed to aid health surveillance assistants (HSAs) in assessing and managing acutely unwell children. The app aimed to improve adherence to clinical guidelines and enhance decision-making processes compared to the paper-based CCM tool traditionally used.	The study used a stepped-wedge, cluster-randomized design. Clusters of village clinics were randomized to determine the sequence of crossover from the control phase (using paper CCM alone) to the intervention phase (using paper CCM and the e-CCM app). The duration of exposure in each phase varied between 2 and 7 weeks.	The stepped-wedge design was selected to ensure all clinics eventually received the intervention, to control for time effects due to evolving conditions, and to facilitate logistical implementation by a single study team.	6	6	2–7 weeks	A total of 6,965 children were recruited for the study, with 3,421 in the control phase and 3,544 in the intervention phase. Specific details on the sample size calculation are not provided in the extracted content.	The primary outcomes were urgent referrals to higher-level health facilities, consultations to village clinics, and hospitalizations within seven days of study enrollment.
30.	Yapa et al., 2020 (55)	7 public-sector primary care clinics in rural South Africa.	The intervention was continuous quality improvement (CQI), which was delivered by trained CQI mentors using standard CQI tools (process maps, fishbone diagrams, run charts, Plan-Do-Study-Act cycles, and action learning sessions). The mentors worked with health workers, including nurses and HIV lay counsellors, to improve antenatal HIV care.	Clusters were randomly switched from control to intervention on pre-specified dates in a random sequence. Investigators and clusters were blinded to randomization until 2 weeks prior to each step​.	The stepped-wedge design was selected for both pragmatic and ethical reasons to ensure that all clinics eventually received the intervention, which aimed to improve the quality of antenatal HIV care in primary care clinics.	8	6	2 months	The power calculation assumed that 40% of pregnant women living with HIV would receive a test for viral load (VL) monitoring and 65% of pregnant women not living with HIV would receive a repeat HIV test without the CQI intervention. They estimated 80% power to detect at least a 15-percentage-point increase in the primary endpoints at the 5% significance level, assuming an intracluster correlation coefficient (ICC) of 0.10 and missing data from 15% of enrolled women.	The two primary endpoints were viral load (VL) monitoring among pregnant women living with HIV and repeat HIV testing among pregnant women not living with HIV.
31.	Yapa et al., 2020 (56)	Primary care clinics	Continuous quality improvement on coverage of antenatal HIV care tests	All clusters receive intervention, in randomised sequence. Masking was only until randomisation status identified at the start of each intervention step.	To establish the effects of CQI on quality of antenatal HIV care in primary care clinics in rural South Africa.	4	7	2 months	-	– Proportion of HIV-infected pregnant women who are on ART and have received an HIV VL test – Proportion of women who are HIV-uninfected at first ANC HIV test with a repeat antenatal HIV test
32.	Graham et al., 2019 (57)	The clusters are defined as hospitals in the study. The study design involves evaluating the effectiveness of an oxygen system intervention in different hospitals over time.	The intervention is an improved oxygen system implemented in hospitals, which includes pulse oximetry and the full oxygen system	Randomization in the stepped wedge design involves sequentially rolling out the intervention to different clusters (hospitals) at regular intervals until all clusters receive the intervention	The stepped wedge design was chosen to ensure that all clusters eventually receive potentially beneficial intervention while allowing for a rigorous evaluation of its effectiveness. This design helps to balance the ethical consideration of providing the intervention to all participants with the need for a control period for comparison	The study involves 12 steps in the intervention rollout, with the full oxygen system being introduced in steps 7–12	The document does not provide the exact number of clusters directly. However, it mentions the use of multiple hospitals as clusters in the analysis	The time between steps is 4 months	The document includes power calculations and intracluster correlation coefficients (ICCs) for different outcomes to determine the required sample size and power for detecting intervention effects	The primary outcome is mortality, specifically child mortality and mortality from acute lower respiratory infections (ALRI) among children under 15 years of age and neonates
33.	Graham et al., 2021 (58)	Clusters are defined at the hospital level, including 12 hospitals that were selected to be representative of secondary-level health facilities providing inpatient care to children in southwest Nigeria	The intervention consists of two phases. Phase 1 introduced pulse oximetry for all admitted children. Phase 2 introduced a full oxygen system that includes standardised oxygen equipment, clinical education and support, technical training and support, and infrastructure and systems support​	Hospitals were randomly allocated to receive the full oxygen system at pre-specified dates using a computer-based random sequence generator​	The stepped wedge design was chosen as it allows all clusters to eventually receive potentially beneficial intervention while enabling a rigorous evaluation of its effectiveness. This design is efficient and pragmatic, enabling the logistics of implementation to be managed effectively	The intervention rollout involved 12 steps, with the full oxygen system being introduced in steps 7–12	12 clusters, each representing a hospital	4 months	The sample size was calculated with sufficient power to detect changes in mortality and clinical practices. The study aimed for 99% power to detect a 10-percentage-point change in the proportion of hypoxaemic children receiving oxygen therapy and various other powers for detecting reductions in mortality rates for different patient groups​	The primary outcome is mortality, specifically child mortality and mortality from acute lower respiratory infections (ALRI) among children under 15 years of age and neonates
34.	Okinyi et al., 2022 (59)	Health care workers (HCWs) from HIV care facilities who received training in waves	A two-day standardized patient actor (SP) training. The training included didactic sessions, exercises in adolescent national guidelines for HIV care, communication skills, values clarification, and motivational interviewing. HCWs rotated through seven video-recorded SP encounters reflecting adolescent care issues followed by a group debriefing session	NA	NA	4	24	9 months	NA	The primary outcome was the self-rated competence of HCWs in providing adolescent-friendly services, specifically targeting their skills in patient-centered communication, clinical assessment, and non-judgmental attitudes towards AYA.
35.	Anger et al., (60)	Secondary level public hospitals	Training and introduction of UBT into routine practice for refractory PPH	Strata contained 2–4 facilities each (not all countries had all three categories of delivery volume). Facilities within strata were randomly assigned to intervention phase using a 1:1 allocation ratio.	This cluster-randomized design was chosen because our research question addressed whether facility-wide introduction of UBT led to reductions in PPH-related morbidity and mortality. Because UBT was hypothesized to be beneficial, we chose a stepped wedge design so that all sites eventually received the intervention	3	18	1 week	We estimated that 43,200 vaginal deliveries would occur over the 15-month study period. This sample allowed detection of a 65% reduction in the primary outcome (80% power, alpha= 0.05, one-tailed test), assuming a rate of 0.4% before UBT introduction (based on previous studies of PPH mortality in Senegal and of UBT introduction). The formula described by Woertman et al. was used to calculate a 4.26 correction factor for the study design effect (assuming an intracluster correlation coefficient of 0.05.)20 Since a before-after study of UBT introduction showed a 74% reduction in invasive procedures6, a detectable difference of 65% was considered reasonable.	The primary composite outcome was PPH-related maternal death and/or invasive procedures.
36.	Ndiaye et al., 2016 (61)	Health post level—54 health posts in rural and semi-urban populations	Delivery of Seasonal Malaria Chemoprevention (SMC) with sulfadoxine-pyrimethamine plus amodiaquine (SPAQ) to children under 10 years of age during the malaria transmission season	Fifty-four health posts were randomized to implement SMC in either 2008, 2009, or 2010. Nine health posts remained without the intervention each year for comparison purposes. The randomization process and the sample size details are described in the study design section	The stepped wedge design was chosen to allow phased implementation and rigorous evaluation of the intervention's safety and effectiveness while ensuring all clusters eventually received the intervention. This design was logistically feasible and allowed for the evaluation of SMC's impact on a large scale	3	54	3 years	The study aimed to cover a population of about 600,000, including approximately 108,000 children aged 0–59 months and 98,000 children aged 60–120 months. The detailed sample size calculation was based on expected malaria incidence and mortality rates, aiming to detect significant differences between intervention and control periods​	The primary outcomes included all-cause mortality, the incidence of malaria cases at outpatient clinics, and the incidence of adverse events related to the SMC drugs. The results for mortality and malaria incidence were described elsewhere in the study
37.	Sturt et al., 2023 (62)	Primary health-care facilities—In Nigeria, there are 35 urban and peri-urban primary health-care facilities in Ibadan, Oyo State. In Tanzania, there are 21 rural and remote primary health-care facilities in five districts within Morogoro Region.	The intervention involved training health workers in remote consulting and providing mobile data allowances. The training was delivered via an online learning smartphone app (Moodle) with online group facilitation. The intervention aimed to support the delivery of remotely delivered primary care.	Health facilities were randomly assigned to sequences for intervention rollout. In Nigeria, there were ten sequences, while in Tanzania, there were seven sequences. Clusters were computer-randomized to receive the intervention at different time points.	The stepped-wedge design was chosen due to capacity constraints and the need for phased implementation. The design allowed all clusters to receive the intervention while accounting for logistical and practical constraints during the COVID-19 pandemic.	10—Nigeria	20—Nigeria	3 months	The sample size for each trial was based on the expected number of eligible patients per cluster (100 eligible patients per cluster in each trial). The trial aimed for sufficient precision to detect clinically relevant changes in outcomes. Power calculations assumed 80% power, a type I error rate of 5%, and an intraclass correlation of 0.05. The estimated sample size was 20 clusters in ten sequences for Nigeria and seven sequences for Tanzania.	Primary outcomes included consultation rates (both remote and face-to-face), prescription rates, and trustworthiness of consultations. These outcomes were measured monthly from open cohorts of patients with diabetes, hypertension, and cardiovascular or pulmonary disease.
						7—Tanzania	20—Tanzania			
38.	Ndirangu et al., 2022 (63)	Level of public health clinics and substance use treatment programs	Women's Health CoOp (WHC), a brief, woman-focused, behavioral, evidence-based intervention.	Eligible participants from each of the eight study clinics were randomly selected within each cycle for the follow-up focus group discussions. The intervention was implemented in a modified stepped-wedge design over four cycles, each lasting six months	To assess the barriers to ART adherence from a socio-ecological framework and to facilitate the phased implementation and rigorous evaluation of the WHC intervention	4	8	Six months	The sample size was based on the eligibility criteria of the 480 women who participated in the WHC implementation science trial	The primary outcomes were the barriers to ART adherence, categorized under intrapersonal-level factors (such as substance use, financial constraints, food insecurity), community-level factors (such as anticipated and enacted stigma, community violence), and institutional-level factors (such as patient-provider relationships, health facility barriers, and environmental stigma)
39.	Pfeiffer et al., 2021 (64)	6 health facilities/clinics	Workflow modifications of existing standard care (SOC). Enhanced adherence and retention package including monthly clinical chart reviews, adherence committees, enhanced counseling, supportive supervision, and systematic patient tracking using SMS texting and phone call reminders	Clinics were randomly allocated within a stepped-wedge design to intervention and control periods. Before randomization, the six sites were stratified by province (three in each), and one site from each province was randomly selected to initiate the intervention at each of three stepped time points.	The stepped-wedge design provided a rigorous and robust test of the intervention. The design allowed for the collection of baseline data and sequential rollout of the intervention to all clusters, enabling comparison of outcomes before and after intervention implementation at each site.	3	6	3 months	Not explicitly detailed in the provided excerpts. However, a total of 761 pregnant women tested HIV positive, initiated ART, and were followed to track retention and adherence outcomes across the six clinics from May 2014 to August 2015.	The primary outcome was retention-in-care, defined as the percentage of HIV-positive pregnant women who returned for their 30-day, 60-day, and 90-day ART medication refills within 25–35 days of their previous refill.
40.	Steinert et al., 2021 (65)	14 government-managed health facilities	The intervention, "Early Access to ART for All" (EAAA), involved immediate initiation of antiretroviral therapy (ART) for all patients living with HIV, regardless of CD4 count thresholds. This includes same-day counselling and ART initiation with continued monthly counselling after initiation.	Health facilities were grouped into seven pairs according to geographic proximity and catchment size. Six pairs of health facilities were cluster-randomized into one of seven sequences using a random number generator in Excel. One pair was excluded from randomization and transitioned first due to the research timeline. The evaluation consisted of eight time periods, with no facility exposed to the intervention in the first period and all facilities exposed in the last period. Randomization was performed by trial statisticians.	The stepped-wedge design allowed for a phased implementation of the intervention, accommodating logistical and practical constraints over a three-year study period.	7	14	The study was conducted over three years, with specific transition periods detailed for each healthcare facility. The final step spanned eleven months.	The sample size for patient exit interviews was determined by funding and feasibility constraints. Simulation-based power calculations were conducted using the “SWSamp” package in R, suggesting that with a two-sided test, α = 0.05, eight periods, a mean of 21 participants per healthcare facility per period, and an intra-cluster correlation of *ρ* = 0.01, the study would have 80% power to detect a 54% difference in healthcare expenditures, 60% power to detect a 40% difference, and 40% power to detect a 34% difference.	The primary outcome was the total private healthcare expenditures in the 12 months preceding the interview, including costs for hospitalizations, travel, food, communication, childcare, and any clinic visit or hospital stay.
41.	Steinert et al., 2020 (66)	14 government-managed health facilities located in the Hhohho region of Eswatini.	The intervention, "Early Access to ART for All" (EAAA), involved immediate initiation of antiretroviral therapy (ART) for all patients living with HIV, regardless of CD4 count thresholds. This includes same-day counselling and ART initiation with continued monthly counselling after initiation.	Health facilities were allocated non-randomly into seven pairs based on geographic proximity and facility catchment size to avoid possible contamination and ensure roughly equal group sizes. The seven pairs were then randomly assigned to one of seven sequences, determining the point in time each facility shifted from the standard of care to the EAAA intervention. Randomization was performed by trial statisticians.	The stepped-wedge design was chosen to accommodate logistical and practical constraints, allowing for a phased implementation of the intervention over three years.	7	14	NA	NA	The primary outcomes were patients’ time use, employment status, household expenditures, and household living standards.
42.	Rewley et al., 2020 (67)	Change agents (CAs) were recruited from waiting rooms of HIV treatment facilities in Dar es Salaam, Tanzania, and their network members (NMs) were recruited directly by CAs.	A behavioral intervention where CAs participated in 10 weekly structured sessions aimed at empowering them to become HIV prevention change agents in their communities. The sessions focused on increasing psychosocial and communication skills through an Appreciative Inquiry approach.	The study was a stepped-wedge randomized controlled trial. All CAs eventually received the intervention but were randomized to one of three waves to determine when they received it.	Spillover effects of HIV knowledge from CAs to their social network members (NMs), examining how the intervention's effects might extend to NMs.	3	3	12 weeks	NA	The primary outcome of this study was the change in NMs’ HIV knowledge. Secondary outcomes included whether the NM was lost to follow-up.
43.	Åhsberg et al., 2023 (68)	Hospital level—involved 3 hospitals in the Greater Accra Region of Ghana, covering a predominantly urban population. Each hospital functioned as a cluster​	Implementation of the Determine TB LAM (lipoarabinomannan) urine test to guide tuberculosis (TB) treatment among severely ill inpatients with human immunodeficiency virus (HIV). The intervention included staff training and performance feedback	Cluster randomization was undertaken by an impartial biostatistician. Each hospital started with a control period and then randomly and sequentially switched over to the intervention period. The randomization order was open-labeled	This design allows for the gradual rollout of the intervention, facilitating logistical management and ensuring that all clusters eventually receive the intervention, while also enabling a rigorous evaluation of its effectiveness	3	3	Each hospital had a control period for a minimum of 22 weeks before transitioning to the intervention period for a minimum of 20 weeks. Enrollment was ceased for 28 weeks due to COVID-19 restriction	The sample size calculation was based on the assumption that 20% of enrolled patients would have TB and that the median time from enrollment to TB treatment would be 10 days. The study aimed to have 95% confidence and more than 90% power to detect a minimum of 2 days reduction in time to TB treatment. The planned total sample was 690 patients enrolled over 82 weeks, but this was affected by the COVID-19 pandemic	The primary outcome was the time in days from enrollment to TB treatment initiation. Secondary outcomes included the proportion of patients diagnosed with TB, initiating TB treatment, and all-cause mortality during 8 weeks of follow-up. Process outcomes related to the Determine LAM test uptake were also assessed​
44.	Uwamariya et al., 2021 (69)	10 district hospitals in Rwanda.	The intervention involved the use of the Dream Warmer, a low-cost, reusable, non-electric infant warmer designed to prevent and treat hypothermia in neonates. It is a heating pad incorporating a phase change material that remains at skin temperature for approximately six hours after being melted by boiled water.	Hospitals were selected based on high neonatal admission rates and varied climates. The warmer was introduced to the hospitals in a random order generated by an Excel computer program. The order of introduction was not concealed but communicated to the investigators to facilitate planning and deployment of staff.	The stepped-wedge design was chosen to provide control data in an ethical and feasible manner while accommodating for multiple uncontrollable confounders. The value of preventing or treating euthermia is beyond the point of equipoise, and a pre-post comparison rather than a randomized trial was considered the most ethical way to obtain control data.	10	10	Two weeks	The sample size calculation was based on the estimate of five infants per hospital per two-week study period with a control rate of 60% successful warming, varying by 10% among hospitals (intraclass correlation 0.04). An average of 2.7 encounters per infant provided 90% power with 5% type I error to detect an improvement of 9%–16% in the rate of successful warming.	The primary outcome was the attainment or maintenance of euthermia, defined as the infant's temperature staying between 36.5 °C and 37.5 °C throughout the encounter or having risen at least 0.5 °C per hour since the initial hypothermic measurement.
45.	Sabourin et al., 2022 (70)	Primary care sites (four rural and four urban)	A package of interventions was developed to improve Kaposi sarcoma care by providing training in clinical recognition and evaluation of Kaposi sarcoma, palliative care and symptom control	The intervention order for urban and rural sites was block randomized. Within blocks, sites were then randomized to obtain the order for implementation of the Intervention Package. This design ensured that urban and rural sites had a similar number of weeks in the pre-intervention and intervention periods.	Sites were randomized to begin the Intervention Package at different times such that the intervention was eventually implemented at all sites during the study.	6	8	7–8 weeks	No Information provided	We hypothesized that the training interventions would lead to more active KS surveillance by primary care providers resulting in increased overall and early-stage KS diagnoses
46.	Mugwanya et al., 2023 (71)	Level of high-volume public HIV care clinics—involved 25 high-volume public health facilities in Central and Western Kenya	Integration of pre-exposure prophylaxis (PrEP) delivery into existing HIV care clinics.	The implementation was conducted in a staged fashion after the training of healthcare providers, with each clinic starting to deliver PrEP at different times according to the randomized schedule	Phased implementation, ensuring all clinics eventually received the intervention while enabling a rigorous evaluation of its effectiveness	Multiple steps, with clinics transitioning to the intervention phase in a staggered manner throughout the study period	25	-	The study involved 4,898 individuals who initiated PrEP, with 2,640 (54%) being female. The sample size calculations aimed to assess patterns of PrEP continuation and medication possession ratios during the first year of use. Various statistical models were used to identify distinct patterns of PrEP coverage​	The primary outcomes were PrEP continuation and PrEP medication possession ratio (MPR) as measures of coverage. Continuation was defined as documented visit attendance or PrEP dispensing from pharmacy records, while MPR was computed based on the number of pills dispensed relative to the days in each computed period​
47.	Omer et al., 2021 (72)	Wards (administrative units) in Toro Local Government Authority (LGA), Bauchi State, Nigeria. Eight wards participated in the trial​	Universal home visits to pregnant women and their spouses. Female and male home visitors provided information about household prevention and management of diarrhoea and immunisation through evidence-based discussions​	An epidemiologist not involved in the fieldwork randomly allocated the eight wards into four waves with one year between each wave. Randomization was done using a computer-generated sequence​	-	4	8	One year	The trial included 1,796 children in the intervention group and 5,109 in the control group. Sample size calculation details were based on maternal health outcomes with the prevalence of diarrhoea and the ability to detect a reduction of 12% with 80% power at the 5% significance level​	The primary outcomes were the prevalence and management of childhood diarrhoea and immunisation status. Intermediate outcomes included household knowledge and actions related to child health.
48.	Farrant et al., 2022 (73)	Three sites in Cape Town, including one 8-hour clinic, one 24-hour clinic, and one district hospital.	The intervention is integrated person-centred palliative care in primary care for patients with COPD. This includes measures to address quality-of-life concerns through holistic and interdisciplinary care approaches.	The study is described as a feasibility stepped-wedge hybrid type II design randomized controlled trial.	-	-	3	3	-	The primary outcomes assessed were the experiences and impact of the lockdown on the primary care received for COPD, focusing on themes such as access to care, medication access, continuity of care, and the impact on wellbeing, behavior, and support​.
49.	Ketema et al., 2020 (74)	Level of healthcare facilities—30 randomly selected health care facilities in Addis Ababa, Ethiopia​	Integrating tuberculosis screening into Integrated Maternal, Neonatal, and Child Illnesses clinics, and enhancing childhood TB case finding in TB DOT unit clinics through contact investigation. This was implemented through a three-day childhood TB training for health providers, providing job aids, updated registers, and monthly performance monitoring​	Health care facilities were randomized into three groups of ten during August 2016-November 2017. Each group of health centers transitioned from the control to the intervention phase in a phased manner	This design allows for all clusters to eventually receive the intervention while providing a rigorous evaluation of its effectiveness​.	4	30	Four months	The study aimed to screen all children attending IMNCI clinics and to trace and screen all under-five contacts of TB cases. The sample size calculations focused on detecting differences in TB screening rates, presumptive TB cases, and IPT initiation rates​	The primary outcomes were the proportion of children screened for TB, the proportion of children with presumptive TB identified, and the proportion of under-five contacts of TB cases traced and screened. Secondary outcomes included IPT initiation rates and the number of TB cases identified​
50.	Powell et al., 2023 (75)	Six district-level hospitals and their attached primary health care centers in Cameroon and Kenya, totaling 32 facilities	The INPUT study evaluates the major components of the Catalyzing Pediatric TB (CaP-TB) project, integrating pediatric TB screening and diagnostics into non-TB child healthcare services at the hospital and primary health care level.	A multi-national, cluster-randomized, stepped-wedge trial.	The integration of pediatric TB services into child healthcare services and compare TB diagnosis and treatment outcomes among children with and without HIV.	-	6	-	NA	The primary outcomes included the prevalence of HIV-TB co-infection, clinical presentations, diagnostic pathways, and treatment outcomes of TB among children with and without HIV.
51.	Multerer et al., 2021 (76)	40 clusters based on administrative units	Adding pyriproxyfen to long-lasting insecticidal nets (LLINs)	LLINs treated with permethrin and pyriproxyfen were incrementally replaced over two years in a SWCRT with 40 clusters		Five clusters switched from control to intervention arm each month from June to September in 2014 and 2015	40	One month	NA	Clinical malaria, measured as fever plus a positive RDT result for Plasmodium falciparum.
52.	Senderowicz et al., 2022 (77)	Six public referral hospitals across different regions of Tanzania	The intervention focused on postpartum intrauterine device (PPIUD) insertion and counselling. It involved training healthcare providers on PPIUD insertion and integrating PPIUD counselling into routine antenatal family planning counselling.	A cluster-randomized stepped-wedge trial approach was used. Hospitals were randomized into two groups, with Group 1 hospitals receiving the intervention earlier and Group 2 hospitals receiving it later. Block randomization was employed to match clusters based on annual obstetric caseloads.	The stepped wedge design allowed the intervention to eventually reach all study hospitals while avoiding contamination and ensuring that the program could be feasibly implemented given the resource constraints.	2	6	Group 1 hospitals received the intervention after 3 months of baseline data collection, while Group 2 hospitals were scheduled to receive the intervention after 9 months. However, there were delays, with Group 2 hospitals actually receiving the intervention approximately 2 months later than planned.	The sample size calculations were performed for the primary study endpoints but were not detailed for the secondary outcomes in this analysis. A total of 22,691 women were screened for eligibility, and 21,033 met the inclusion criteria. The full analytic sample included 19,631 women, with 10,078 in the counselled analytic sample.	The primary outcome was the percent uptake of PPIUD, defined as the proportion of all women who received a PPIUD divided by the number of women who delivered in one of the study hospitals over the course of the study period. Secondary outcomes included the percentage of women who received PPIUD counselling and the percentage of PPIUD acceptors who had PPIUD expulsions.
53.	Kumbani et al., (78)	Community level—three communities in Phalombe, a rural district in southern Malawi	Mzake ndi Mzake (Friend-to-Friend) 6-session peer group intervention, delivered by trained community volunteers. The sessions included HIV transmission and prevention facts, basic reproduction and sexuality information, prevention and treatment of other sexually transmitted infections, testing and treatment as prevention, partner communications, correct condom use, and engaging in community-wide HIV prevention.	Three communities were randomly assigned to implement the Mzake program sequentially. The implementation order was determined by drawing community names from a basket	The stepped wedge design was chosen to efficiently use repeated measures, allowing participants to serve as controls until their community implemented the program. This design also facilitated phased implementation, ensuring all clusters received the intervention while enabling a rigorous evaluation of its effectiveness​	3	3	NA	Sample size calculations for the longitudinal step-wedge design study were carried out using simulations for two knowledge outcomes. Considering an attrition rate of 25%, a total sample size of 432 individuals per age group was proposed to ensure 80% statistical power for the detection of desired effect sizes. The total sample included 1,008 individuals, with 460 adults and 548 youth providing baseline data	The primary outcome was HIV prevention knowledge, assessed through two indicators: UNAIDS comprehensive knowledge (correctly answering five HIV prevention questions) and a 9-item HIV/PMTCT Knowledge Index. The effectiveness of the intervention was evaluated by comparing the knowledge levels between the control and intervention groups at different time points
54.	Yan et al., 2017 (79)	46 rural government clinics in Chongwe, Kafue, and Luangwa districts of Zambia.	The intervention aimed to improve primary healthcare through the use of standardized protocols for common visits, onsite electronic medical records (EMR), and ongoing mentoring to improve key indicators​.	The study was designed as a randomized stepped-wedge trial.	This design helps in evaluating the impact of the intervention while ensuring all clinics eventually receive it.	-	46	-	-	The primary goal was to evaluate the impact of the intervention on hypertension management, focusing on process and outcome indicators like blood pressure measurement, hypertension diagnosis, and treatment​.
55.	Gichane et al., 2020 (80)	Four health clinics paired with four substances use rehabilitation programs.	The intervention is the Women's Health CoOp (WHC), a gender-focused HIV and alcohol risk-reduction intervention. It includes interactive group workshops combining risk-reduction information about alcohol and other drugs (AODs), HIV prevention, treatment initiation, the importance of treatment adherence, other sexually transmitted infections, and gender-based violence prevention with behavioral skills training, sexual empowerment, negotiation, and communication skills.	Four health clinics were paired with four substances use rehabilitation programs and randomized into four cycles (steps).	The study aims to evaluate implementation outcomes in real-world settings using a phased approach, allowing for systematic assessment and control of intervention timing across different clusters.	4	8	6 months	A total of 480 patients were enrolled in the study, with 120 patients per cycle. Eligibility criteria included being between 18 and 45 years old, self-reporting weekly AOD use during the previous 3 months, reporting unprotected sex with a male partner in the past 6 months, having a positive verifiable HIV test result, intending to remain in the area for at least the next 6 months, providing contact information, and being willing to participate in AOD screening.	The primary outcomes assessed were adoption, acceptability, appropriateness, cost, and fidelity of the intervention.
56.	Fawzi et al., 2019 (81)	Groups of people living with HIV (PLHIV) and their social network members	The NAMWEZA intervention involves 10 weekly group sessions aimed at improving self-esteem, self-efficacy, and promoting safer sexual behaviors. The sessions include topics such as love, relationships, assertiveness skills, and disclosure of HIV status.	Randomization was done within four age/sex-specific strata (women <35 years, women ≥35 years, men <40 years, men ≥40 years). Participants were assigned random numbers, and a random number generator was used to allocate them to one of three steps at a 1:2:2 ratio.	The stepped-wedge design was chosen because it is efficient and more feasible logistically, allowing the intervention to be phased in over time to include all study participants.	3	3	3 months	The sample size calculations assumed an alpha level of 0.05, a baseline prevalence of 10%, and an OR of at least 1.5 for the uptake of HIV services to reach a power of 80%. For unprotected sex and IPV, the study had greater than 80% power if the OR estimates were 0.70 or lower. These calculations accounted for 85% and 80% retention rates for Change Agents and Network Members, respectively.	Primary outcomes included uptake of HIV services, self-efficacy, self-esteem, HIV risk behavior, and intimate partner violence (IPV).
57.	Maheu-Giroux et al., 2013 (82)	Health center level—involved 30 health centers in Addis Ababa, Ethiopia	Integrating tuberculosis (TB) screening into Integrated Management of Neonatal and Childhood Illness (IMNCI) clinics and enhancing childhood TB case finding in TB DOTS (Directly Observed Treatment, Short-course) clinics through contact investigation.	30 health centers were randomly assigned to three groups, each with 10 health centers, which started the intervention phase by phase. Allocation was not concealed, but clinicians, TB, and IMNCI officers were blinded to the order of entry into the intervention until each group of health centers was enrolled	This design allowed for the phased implementation of the intervention and provided a rigorous evaluation of its effectiveness while ensuring that all clusters eventually received the intervention	3	30	Four months	NA	The proportion of children screened for TB, the proportion of children with presumptive TB identified, and the proportion of under-five contacts of TB cases traced and screened.
58.	Patel et al., 2005 (83)	Health facility catchment area	Supplemental feeding with Ready-to-Use Therapeutic Food (RUTF) for children at risk of malnutrition	A prospective systematic allocation using a stepped-wedge design was employed because randomized assignment to supplementary feeding with RUTF or corn/soy-blend was not possible due to resource constraints and cultural beliefs	The stepped-wedge design was chosen because it was not possible to randomly assign participants due to resource constraints and cultural beliefs. Additionally, the design controlled for bias introduced by seasonal variations in childhood malnutrition	4	7	3 weeks	A sample size of 250 children was calculated to provide 95% confidence and 80% power to detect a minimum of 25% absolute increase in recovery rate, assuming a 1:4 allocation of participants into corn/soy-blend and RUTF groups with 50% recovery in the corn/soy-blend group	The primary outcomes were recovery (defined as weight-for-height >90%) and the rate of weight gain
59.	Wyatt et al., 2024 (84)	12 public health facilities in Kampala and Wakiso districts, in central Uganda	The intervention consisted of three components: Clinic-wide trainings on PrEP delivery for ART providers. Provision of lamivudine/tenofovir disoproxil fumarate (3TC/TDF) as PrEP. Ongoing technical assistance (TA) to facility-based staff from the Partners PrEP Program study team	The study was designed as a stepped-wedge cluster randomized trial.	The stepped-wedge design was chosen to allow for the staggered implementation of the intervention across the different facilities	-	12	-	NA	The primary goal was to evaluate the impact of PrEP use on ART initiation and persistence.
60.	Kiwanuka et al., 2023 (85)	Health facility level—18 health facilities across 15 districts in Uganda	Implementation of the 99DOTS digital adherence technology for tuberculosis (TB) treatment supervision. This technology included self-reporting of TB medication adherence via toll-free phone calls, automated text message reminders, and support actions by health workers monitoring adherence data.	Health facilities were randomly assigned to receive the intervention at different times using a stepped-wedge cluster randomized trial design. The sequence of implementation was randomly determined.	Phased introduction of the 99DOTS intervention, ensuring that all clusters eventually received the intervention while enabling a rigorous evaluation of its impact on TB treatment adherence.	The study involved multiple steps, with clusters transitioning from control to intervention at different times throughout the study period.​	18	NA	The sample size calculation assumed that 20% of enrolled patients would have TB, and the time from enrollment to TB treatment initiation would be a median of 10 days. The study aimed to have 95% confidence and more than 90% power to detect a minimum of 2 days reduction in time to TB treatment. The planned total sample was 690 patients enrolled over 82 weeks.	The primary outcome was the time in days from enrollment to TB treatment initiation. Other key outcomes included the proportion of patients routinely investigated for TB, diagnosed with TB, initiated on TB treatment, and all-cause mortality during 8 weeks of follow-up​.
61.	van den Broek et al., 2019 (86)	Healthcare facilities (HCFs) across 11 districts in South Africa with the highest maternal mortality. A total of 127 healthcare facilities were included in the study.	The intervention involved "skills and drills" training workshops in emergency obstetric and newborn care (EmOC&NC) for maternity staff. The training covered essential knowledge and skills required to recognize and manage major causes of maternal and newborn death, including maternal and newborn resuscitation, management of severe pre-eclampsia and eclampsia, obstetric hemorrhage, sepsis, obstructed labor, and assisted vaginal delivery.	The sequence in which all districts received the EmOC&NC training was randomized. One district was randomly selected to be a pilot site and was excluded from the main study. The sequence of implementing the intervention was determined using simple random sampling by an independent person drawing district names from a hat.	The stepped-wedge design was chosen to accommodate logistical constraints, allowing for phased implementation of the intervention while ensuring that each district received the training.	10	11 districts—127 healthcare facilities	2 months	The sample size was determined by the number of clusters available for inclusion and the number of observations within each cluster, dictated by the number of births over the 26-month study period. No *post hoc* power calculation was performed.	Primary outcome measures at the healthcare facility level included stillbirth rate (SBR), early neonatal death (ENND) rate, institutional maternal mortality ratio (iMMR), and direct obstetric case fatality rate (CFR).
62.	Ogbuoji et al., 2019 (87)	Level of public-sector healthcare facilities	Implementation of the Early Access to Antiretroviral Therapy for All (EAAA) strategy, providing immediate access to HIV treatment for all patients living with HIV attending public-sector clinics	14 healthcare facilities were randomized into pairs and then randomized to transition from the standard of care to the EAAA strategy at different time points, with two facilities transitioning every four months	To allow phased implementation of the EAAA strategy, ensuring all facilities eventually received the intervention while enabling rigorous evaluation of its impact on patient satisfaction​	7	14	Four months	A total of 2,629 patient interviews were conducted over the study period. The study was powered to detect significant differences in patient satisfaction with a sample size of approximately 187 patients per step (14 clusters × 13.36 patients per cluster)​	The primary outcome was patient satisfaction, measured as overall patient satisfaction and satisfaction with four specific domains: wait time, consultation time, involvement in treatment decisions, and respectful treatment​
63.	Kohler et al., (88)	Health facility level—conducted at 24 health care facilities (clusters) in Kenya	Standardized patient (SP) training for health care workers (HCWs) to improve communication and interpersonal skills in dealing with adolescents and young adults living with HIV. The training included adolescent care, values clarification, communication skills, and motivational interviewing, with 7 SP encounters followed by facilitated feedback of videotaped interactions.	Facilities were randomized to the timing of the intervention in waves, ensuring each facility eventually received the training intervention. The randomization was done using a stratified randomization method based on region and facility size (high volume vs. medium volume)​.	Allows for phased implementation, which was logistically feasible given the constraints of the training program. It also enabled the evaluation of the intervention while ensuring that all clusters eventually received the potentially beneficial training​	4	24	Each training wave was conducted 9 months apart, with training sessions for each group of facilities taking 2–4 weeks to complete​	The study was designed with 80% power to detect a 15% difference between control and intervention periods, assuming a coefficient of variation of 0.25. It was estimated that a minimum of 5 adolescents per clinic per time point were needed. The primary analysis included medical records for 4,595 youth living with HIV (YLHIV) across the 24 clusters	The primary outcome was youth retention in HIV care, specifically during early engagement. Engagement was defined as returning for a follow-up visit within 3 months of enrollment or re-engagement in care after a lapse of at least 3 months
64.	Geldsetzer et al., 2020 (89)	Health care facilities	Training of health care providers for the provision of PrEP	The randomization was performed by S.K. using Stata version 15.1. No stratification was used in the randomization.	We chose a stepped-wedge instead of a parallel-arm randomized design for the evaluation of the PPP because it is statistically more efficient when the outcome is observed at the cluster rather than the individual level and because the design has the advantage that all participating clusters receive the intervention during the study.	3	3	121 months	Before the start of the trial, we estimated the power for the primary endpoint of the trial using simulation. With 1,000 simulations, an intracluster correlation coefficient of 0.05, no time effect, and a within-facility SD of 2, we had 84% power at the α < 0.05 level to detect a RR of 1.4 comparing the intervention to the control phase.	The level of PrEP uptake and retention when PrEP is offered to anyone aged 16 years and older who is at risk of acquiring HIV or who requests PrEP, test the impact of a health care facility–based PPP on PrEP uptake, and characterize stakeholders’ views on how PrEP uptake could be further increased.
65.	Naidoo et al., 2017 (90)	Health facility level—involved primary health care (PHC) clinics in Cape Town, South Africa	Introduction of the Xpert® MTB/RI*F* test for tuberculosis (TB) diagnosis and drug susceptibility testing. This test was phased in to replace the traditional smear/culture-based diagnostic algorithm	The clinics were randomized to the order in which they would receive the intervention. The randomization ensured that all clinics eventually received the intervention while allowing for a staggered rollout	This design enabled the researchers to evaluate the intervention's impact over time while ensuring all clusters eventually received the intervention	7	142	NA	NA	The primary outcome was the proportion of TB cases with drug susceptibility tests (DST) undertaken and the proportion of multidrug-resistant tuberculosis (MDR-TB) cases diagnosed pre-treatment and during the course of 1st-line TB treatment
66.	Naidoo et al., 2016 (91)	PHC site consisted of municipal and provincial health facilities linked to their satellite and mobile facilities.	Xpert-based algorithm to replace the smear/culture-based diagnostic algorithm for all presumptive TB cases​	NA	Practical, financial or logistic constraints prevented simultaneous introduction of Xpert-based algorithm to all facilities	5	142	1 month	NA	The primary outcome was the proportion of TB cases with drug susceptibility tests (DST) undertaken and the diagnosis of MDR-TB pre-treatment and during the course of first-line TB treatment.
67.	Shete et al., 2023 (92)	10 community health centers across eight districts in Uganda	An unconditional cash transfer intervention of UGX 20,000 (∼USD 5.39) was provided to all eligible people undergoing sputum-based testing for pulmonary TB during the intervention period at participating health centers via mobile money	Eligible health centers were randomized using a simple, unrestricted two-stage process. First, they were matched into clusters based on pre-randomization data of patient volume. Second, clusters were randomly assigned into the sequence order in which they would switch into the intervention period during a stakeholder-led randomization ceremony whereby health center representatives chose a numbered ball from an opaque bag, indicating their sequence order	The study aimed to assess the feasibility and potential effectiveness of a cash transfer intervention on completion of TB diagnostic evaluation and treatment initiation in a programmatic setting. The stepped wedge design was likely chosen to ensure that all clusters eventually receive the intervention while also allowing for a comparison between pre-intervention and intervention periods	6	10	1 month	The sample size calculation assumed a type I error of 5% and 80% power. Data from 2018 to 2019 suggested an intracluster correlation coefficient of 0.11, a geometric mean number of people initiating treatment within 14 days of 2.22 (95% CI 1.80–2.74), and a standard deviation of 2.09. A sample size of 240 people diagnosed with microbiologically confirmed TB was needed to detect a minimum odds ratio between pre-intervention and intervention periods of 1.09	The primary outcome was the number of people initiating treatment for microbiologically confirmed TB within 2 weeks of presenting to the health center for TB evaluation​
68.	Heffron et al., 2022 (93)	ART clinic level	Integration of pre-exposure prophylaxis (PrEP) delivery for HIV-negative members of serodifferent couples into existing ART programs for people living with HIV. This included PrEP training for ART providers, ongoing technical assistance, and a provisional supply chain mechanism for PrEP medication.	ART clinics were randomly assigned in a 1:1:1 ratio to one of three “clinic groups” and each group was then assigned to launch the integrated PrEP program during one of three steps​	The stepped wedge design was chosen to evaluate the impact of integrating PrEP into existing ART clinics, allowing all clusters to eventually receive the intervention while enabling a rigorous evaluation of its effectiveness. This design permits the structured rollout of the intervention, facilitating management and assessment of its impact	3	12	Specific duration between steps is not explicitly mentioned in the snippets, but the baseline period lasted for 265 days, step 1 lasted 196 days, step 2 lasted 176 days, and step 3 lasted 371 days	Sample size was based on the ART initiation outcome. Power calculations indicated that with 12 clinics, 3 steps, and 104 couples per clinic group, the study would have 80% power to detect an increase in ART initiation from 50% to 65% within 3 months. Adjustments were made based on observed data during the study	The primary outcomes included PrEP initiation, 3- and 6-month PrEP persistence, ART initiation, and 6-month viral suppression. Viral suppression was defined as HIV RNA <1,000 copies/mL 6 months from initiating ART
69.	Thompson et al., 2022 (94)	18 health facilities, including 5 referral hospitals, 10 general hospitals, and 3 district health centers in Western, Central, and Eastern Uganda with National TB and Leprosy Program–affiliated TB treatment units.	The intervention involved implementing 99DOTS, a digital adherence technology for tuberculosis treatment support. This technology uses a specially designed medication blister pack that reveals a unique toll-free phone number when patients remove pills. Patients call the number to confirm their dose, and an automated system records their dose confirmation in real-time. Health workers use an online dashboard to monitor adherence and provide additional support through targeted SMS messaging, phone calls, and home visits.	Using a stepped-wedge design, three clinics were randomly chosen to introduce the 99DOTS intervention each month. The first month post-transition served as a “buffer” period. This process continued until all 18 clinics had implemented the intervention.	The stepped-wedge design was chosen to facilitate phased implementation and to assess the impact of the intervention over time, ensuring that all clinics eventually received the intervention while allowing for logistical and operational adjustments.	The intervention was rolled out in steps, with three clinics transitioning to the intervention each month.	18	One month	NA	The primary outcome was the cost and cost-effectiveness of 99DOTS in supporting TB treatment adherence. The effectiveness was measured as the cost per patient successfully completing treatment and the incremental cost-effectiveness per additional treatment success.
70.	Ononge et al., 2014 (95)	Pregnant women attending antenatal care at six health facilities in Mpigi district, Uganda	Antenatal distribution of misoprostol for self-administration after home birth or when oxytocin is not available during facility delivery	Stepped-wedge cluster-randomized trial design	-	-	6	-	-	Haemoglobin status and prevalence of anaemia among pregnant women (Hb < 11.0 g/dL)​
71.	Ononge et al., 2015 (96)	Health facility catchment area	Giving women 600 micrograms of oral misoprostol to self-administer after childbirth if delivery happened outside a health facility or when there was no oxytocin at the health facility	The random sequence for starting the intervention was determined using a computer-generated number sequence. The principal investigator implemented the randomization	The stepped-wedge design was chosen because current evidence on misoprostol use and postpartum hemorrhage would render a placebo-controlled trial unethical, and all facilities ultimately get the intervention.	4	6	2 months	The sample size was calculated considering a between-cluster correlation coefficient *km* = 0.2, a minimum of 200 pregnant women per health facility in each phase, and a proportion experiencing postpartum hemorrhage of 12.0%.	The primary outcome was postpartum hemorrhage (PPH), defined as a drop in maternal hemoglobin by 2 g/dL or more, lower than the prenatal hemoglobin.
72.	Winani et al., 2007 (97)	10 surveillance sites across two rural districts of Mwanza Region, Tanzania​.	The intervention involved the use of a clean delivery kit combined with education about the “six cleans” to prevent cord infection and puerperal sepsis. The delivery kit included a plastic sheet, soap, razor blade, string to tie the umbilical cord, and pictorial instructions.	The clusters (surveillance sites) received the intervention in a randomized order. The stepped-wedge design ensured that all clusters eventually received the intervention while maintaining randomization.	The stepped-wedge design was chosen to overcome ethical issues related to withholding an effective intervention and logistical issues related to simultaneous implementation in multiple clusters.	-	10	-	-	The primary outcomes were the incidence of cord infection in newborns and puerperal sepsis in mothers. Newborns whose mothers used the delivery kit were 13.1 times less likely to develop cord infection, and mothers who used the kit were 3.2 times less likely to develop puerperal sepsis​.
73.	Dryden-Peterson et al., 2015 (98)	20 antenatal clinics	An automated SMS-based CD4 result platform that wirelessly distributed CD4 results to portable SMS-enabled thermal printers located in each of the study antenatal clinics	The study utilized a stepped-wedge cluster randomized design. Two clinics were randomly selected every 4 weeks to receive the intervention.	This design was selected to ensure that all clinics could benefit from the intervention, permit adjustment for time effects given evolving programs, and enable effective implementation by a single study team.	4	20	4 weeks	-	The primary study endpoints were the proportion of HIV-infected, ART-naïve pregnant women completing phlebotomy for CD4 enumeration by 26 weeks gestation and initiating ART by 30 weeks gestation.
74.	Khan et al., 2020 (99)	Healthcare facility level—involved 14 public sector health facilities in Eswatini	Access to antiretroviral therapy for all HIV-positive individuals, regardless of CD4 count or disease stage, compared to the standard of care. The intervention included community sensitization, health talks, posters about early access to ART for all (EAAA), and clinical mentors supporting the nursing staff with the introduction of EAAA.	14 health facilities were paired and randomly assigned to stepwise transition from standard of care to EAAA. Randomization was done in pairs, matched by catchment size and geographic proximity	This design allowed for a phased implementation, ensuring all clusters eventually received the intervention while enabling a rigorous evaluation of its effects	4	14	4 months	-	The primary outcomes were retention in HIV care and viral suppression six months or more after ART initiation among those retained. Combined retention and viral suppression were also assessed as a secondary endpoint
75.	Rokicki et al., 2021 (100)	30 rural maternity facilities in Uganda with unreliable lighting	The intervention was the We Care Solar Suitcase, a complete solar electric system providing medical lighting and electrical power for charging small medical devices and mobile phones.	Facilities were randomized into one of two sequences, with the first sequence receiving the intervention between the first and second periods of data collection, and the second sequence receiving the intervention between the second and third periods of data collection.	The stepped wedge design was necessary due to limited resources, allowing a staggered rollout of the intervention.	Two sequences, effectively creating three steps (baseline, first intervention, and second intervention)	30	3 months	Sample size calculations were conducted using the stepped-wedge function in Stata v15. The estimates assumed 22 births per facility (average cluster size) for two steps, 13 clusters randomized at each step, 80% power, and α=0.05. The minimum detectable effect size for adequate light was 13 percentage points, and for quality, it was 11 percentage points.	The primary outcomes were the quality of intrapartum care measured by a 20-item and a 36-item index, a 6-item index of delays in care provision, and the percentage of deliveries with bright light, satisfactory light, and adequate light.
76.	Sarrassat et al., 2021 (101)	Primary health facilities in two regions of Burkina Faso: Boucle du Mouhoun and Nord	The Integrated eDiagnosis Approach (IeDA) intervention centered on an electronic Clinical Decision Support System (eCDSS) developed in line with national Integrated Management of Childhood Illness (IMCI) guidelines. The intervention included a six-day training course for healthcare workers (HCWs) on IMCI guidelines and the use of the eCDSS, a quality assurance coaching system, a supervision system with monthly visits, and a health information system based on data collected through the eCDSS.	The study used a stepped-wedge cluster-randomized design, with health districts (clusters) receiving the intervention at different time points in a randomized order. The randomization ensured that intervention and control clusters were balanced with respect to region and the performance-based financing (PBF) intervention.	The stepped wedge design was chosen because some components of the intervention could only be delivered at the district level, and rolling out the intervention in a phased manner was more practical for the implementing agencies.	Nine steps, one every four months, were initially planned. Due to funding and logistic issues, only four steps were implemented, with the first step serving as the baseline.	8	Four months	The sample size was determined using the method described by Hussey and Hughes, assuming a design effect of 2 due to clustering within facilities and a between-cluster coefficient of variation of 0.3. With a harmonic mean of ten children seen at each of the ten selected health facilities per district and 800 children per step, the trial would provide 90% power to detect an increase in any of the primary outcomes from 25% to 33%.	The primary outcomes were: Overall adherence to clinical assessment tasks. Overall correct classification ignoring the severity of the classifications. Overall correct prescription according to HCWs’ classifications.
77.	Paddick et al., 2017 (102)	Health facility catchment area in rural Tanzania	Cognitive Stimulation Therapy (CST), a group-based psychosocial intervention for dementia	Randomization was done by CST group membership. Two groups were randomly allocated to “immediate start” and two to “delayed start” sessions using a random number generator by the study statistician	The stepped-wedge design was chosen because running all four CST groups concurrently was not possible due to resource limitations, and the delayed start group could act as controls for the immediate start group during the first round of intervention and assessments	1	4	The CST sessions ran from September to December 2014, with immediate start group sessions from week 3 to 9 and delayed start group sessions from week 12 to 18	A sample size of 30 was deemed sufficient for the study of feasibility. For the primary clinical outcome (change in WHOQOL-Bref score from baseline to follow-up), a sample size of 33 was necessary to detect a mean change of 1.5, assuming a standard deviation of 2.5, at 90% power and 5% significance level​	The primary outcome was change in quality of life, assessed using the Brief WHO Quality of Life (WHOQOL-Bref) scale​
78.	Psaki et al., 2022 (103)	18 randomly selected communities in KwaZulu-Natal, South Africa.	The Asibonisane Community Responses Program, which includes the Stepping Stones participatory HIV prevention program focused on strengthening relationships and communication.	Communities were randomized to one of three intervention rollout arms in a stepped wedge design.	To allow all communities to eventually receive the intervention while evaluating its effectiveness over time.	3	18	8 months	Based on a study design with three implementation steps and four rounds of data collection, a power criterion of 0.80, alpha coefficient of 0.05, intra-cluster correlation of 0.05, and a conservative assumption of baseline prevalence (45%) of key outcomes, the minimal sample size required at the end of the study was approximately 1,260 individuals (630 men, 630 women), distributed across 18 clusters with 35 men and women in each cluster. Assuming 15% loss to follow up, an estimated baseline sample size of 1,500 individuals (750 men, 750 women) was specified. A minimum 10% percentage point effect size (change due to intervention) was specified for the primary outcome indicators.	The primary outcomes included increased use of HIV prevention and treatment services, reduction in sexual risk behaviors, and reduction in the experience and perpetration of sexual and gender-based violence.
79.	Agizew et al., 2019 (104)	A cluster was defined as an HIV care and treatment clinic. Five district hospitals and 17 primary healthcare facilities	Training of clinicians and laboratorians on the four-symptom tuberculosis screening algorithm and Xpert testing.		A stepped-wedge rather than parallel group design was chosen because the Xpert package was expected to be beneficial for patients and the trial was part of a national rollout.	13	22	2 weeks	To answer the first XPRES primary study question with > 80% power and alpha at 0.05, assuming that smear and Xpert TB diagnostic algorithm sensitivities are about 62.5% and 82.4%, respectively, about 9,614 new HIV clinic enrollees needed to be enrolled (3,266 smear arm and 6,348 Xpert arm, after GeneXpert instrument roll-out)	TB treatment outcomes were available for 203/256 (79.3%) patients (smear arm, 46. Xpert arm, 157). The other 53 TB patients were transferred out or not evaluated. Although not statistically significant, patients with TB diagnosed via smear were more likely to have unfavorable treatment outcomes than those with TB diagnosed via Xpert MTB/RIF (adjusted hazard ratio: 1.40; 95% CI: 0.75–2.26; *p* = 0.268;
80.	Odeny et al., 2019 (105)	Public health facilities supported by the Family AIDS Care and Educational Services (FACES) program to provide PMTCT (Prevention of Mother-To-Child Transmission) services in Kenya	Texting Improves Testing (TextIT) intervention, a theory-based text messaging system designed to improve infant HIV testing rates and maternal retention in PMTCT programs	20 clusters were randomly allocated to either begin implementing the intervention immediately or to start after a six-month delay. The randomization was stratified by clinic volume and experience level, with the sequence generated by an independent biostatistician	This design allowed for phased implementation and ensured that all clusters eventually received the intervention, while providing rigorous evaluation over time.	2	20	Six months	The sample size determination was based on the primary outcome of infant HIV virologic testing. Assuming a coefficient of variation between clusters of 0.25 and 40% of infants in the control group being tested, the study aimed for a sample size of 2,508 women at 20 clinics to achieve 90% power to detect an increase to 53% tested in the intervention group	The primary outcomes were infant HIV virologic testing within 8 weeks after birth and maternal retention in care during the first 8 weeks after delivery. These were assessed by reviewing medical records and clinic data
81.	Homan et al., 2016 (106)	Household levels	Installation of solar-powered odour-baited mosquito trapping systems (SMoTS) to households. The traps aimed to reduce malaria transmission by attracting and killing host-seeking Anopheles mosquitoes both indoors and outdoors	Households were assigned to clusters using the travelling salesman algorithm. Clusters were randomized to receive the intervention (SMoTS installation) in a specific order until all clusters had the system installed. A total of 27 randomizations were generated, with one randomization sequence selected via a public lottery on the island	The stepped wedge design was chosen to allow for gradual implementation of the intervention across all clusters, facilitating logistical management and ensuring that all participants eventually received the potentially beneficial intervention. This design also enabled a rigorous evaluation of the intervention's effectiveness while addressing ethical considerations of providing the intervention to all participants	27	81 clusters, each consisting of 50–51 households	The installation of SMoTS was done weekly, with each cluster receiving the intervention at intervals that allowed for installation completion before moving to the next cluster	The sample size was calculated to have 80% power to detect a 23% reduction in clinical malaria over six surveys. The intra-cluster correlation coefficients were estimated from the baseline period data to guide the sample size calculation and power estimation	The primary outcome was clinical malaria, diagnosed during active health and demographic surveillance of all individuals. Clinical malaria was identified through household visits, temperature measurements, and rapid diagnostic tests for Plasmodium species. The primary analysis compared clinical malaria incidence between intervened and non-intervened clusters during the rollout period
82.	Wechsberg et al., 2021 (107)	4 HIV/antenatal clinics and 4 substances use treatment rehab clinics	The intervention involved the Women's Health CoOp (WHC) intervention, a 2-session group workshop focused on reducing various HIV-related risks for populations of women who use alcohol and other drugs. It included facts about HIV, STIs, TB, alcohol, and other drugs; skills to negotiate safer sex; and strategies to reduce alcohol and drug risk	Clinics were randomly assigned to one of four cycles. Randomization was conducted using a SAS computer program to assign clinic pairs matched by geography—one HIV clinic and one substance use treatment clinic—to 1 of 4 cycles. The implementation strategies and the WHC intervention were conducted one cycle at a time	The stepped-wedge design was used to overcome ethical issues of traditional cluster-randomized trials where control clusters would not receive an efficacious intervention and logistical issues of implementing an intervention simultaneously in a large number of clusters. This design was suitable for assessing the implementation of interventions with established efficacy.	4	8	6 months	NA	The primary outcomes included ART adherence and reduction in alcohol use disorder risk. ART adherence was measured using a visual analogue scale (VAS), and alcohol use disorder risk was assessed by self-report and breathalyzer tests​.
83.	Mutale et al., 2023 (108)	Primary health care facility level—42 predominantly rural health centers and their surrounding communities in Lusaka Province, Zambia	Better Health through Mentoring and Assessment (BHOMA) project, which comprised intensive clinical training and quality improvement measures, support for commodities procurement, improved community outreach, and district-level management support. The intervention aimed to improve clinical care at the primary care level and increase community demand through improved confidence in the health system	The 42 clusters were randomly assigned to start the intervention in seven steps, each step having six clusters, 3 months apart. Randomization of the clusters into the seven steps was stratified by district. The randomization was done by a statistician who generated a random implementation schedule using Stata version 15. The order of rollout of the intervention could not be blinded	Phased implementation, ensuring all clusters eventually received the intervention while enabling a rigorous evaluation of its impact	7	42	Three months	The sample size was based on standard formulas for parallel cluster randomized trials, adjusted for the design effect of a stepped wedge design. The plan was to conduct three surveys of the 42 clusters, each recruiting 150 households per cluster. After observing lower-than-anticipated mortality rates in the baseline survey, the sample size was doubled to 300 households per cluster in survey rounds 2 and 3. This adjustment aimed to ensure 90% power to detect a 35% reduction in mortality	The primary outcome was age-standardized mortality among individuals aged > 28 days and < 60 years.
84.	Killam et al., 2019 (109)	Antenatal care clinic level—involved eight public sector ANC clinics in the Lusaka district, Zambia	Integration of antiretroviral therapy (ART) services into ANC clinics to increase the proportion of HIV-infected, ART-eligible pregnant women initiating ART during pregnancy. This included providing ART within the ANC clinics, which involved ART training for ANC staff and ongoing technical assistance	The eight ANC clinics were matched into four pairs based on the number of HIV-infected pregnant women expected at each site. The intervention was rolled out in a phased manner, one site at a time, in a stepped-wedge design. This approach allowed each clinic to act as its own control while transitioning to the intervention period	This design allowed for the phased implementation of the intervention and rigorous evaluation while ensuring all clinics eventually received the intervention	The intervention rollout involved multiple steps, with one new site upgrading its services to provide ART in the ANC clinic each month	8	One month	NA	The primary outcomes were the proportion of ART-eligible women enrolling into HIV care within 60 days of HIV diagnosis and initiating ART during pregnancy. These outcomes were measured and compared between the control cohort (referral to ART) and the intervention cohort (integrated ART in ANC)
85.	Wichaidit et al., 2019 (110)	30 public primary schools in Kisumu County, Kenya.	The intervention involved providing Povu Poa handwashing stations with foaming soap dispensers and a behavior change program based on disgust and social norms.	Schools were randomly allocated into three groups of ten schools each. The order in which each group of schools received the intervention was randomly assigned.	The stepped-wedge design was chosen because the intervention posed no harm and provided benefits by creating places to wash hands with soap, making it unethical to withhold the intervention. Additionally, the sequential roll-out minimized logistical constraints.	3	30	Group 1: 3–5 weeks Group 2: 6–8 weeks Group 3: 19–24 weeks	NA	The primary outcomes included the availability of handwashing materials at handwashing places and observed handwashing behavior after toilet use among schoolchildren.

### Cluster definitions

**Figure 4:**
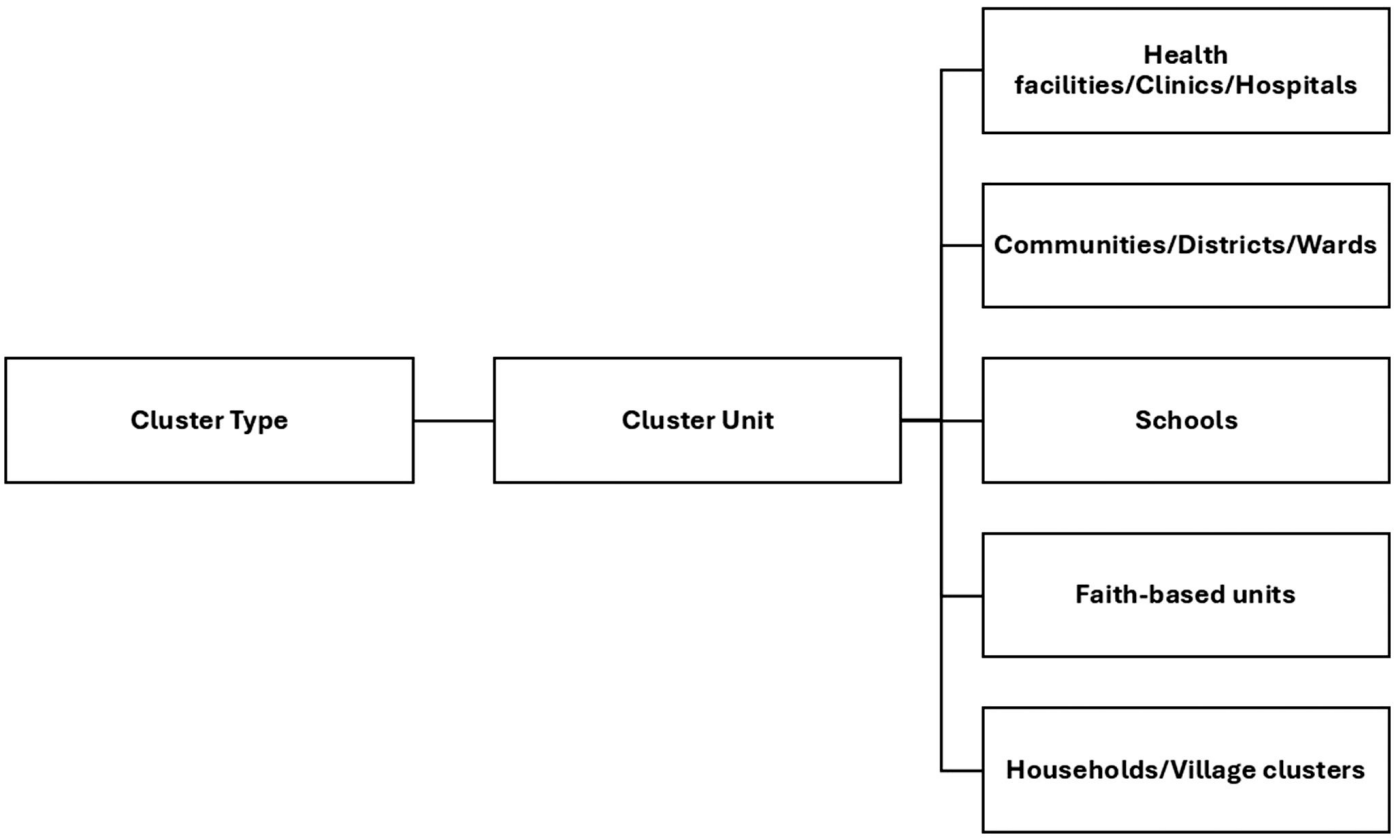
Types of cluster units used in stepped-wedge studies. This schematic summarises the types of clusters used in included studies. Cluster units commonly included health facilities/clinics, communities or districts, schools, faith-based units, and household/village clusters, reflecting the flexibility of stepped-wedge designs across diverse implementation settings.

Figure 4 shows the definition of clusters in SWD in SSA. Health facilities, strategically chosen as clusters in numerous studies, underscore the emphasis on healthcare delivery and interventions within controlled environments. The everyday use of hospitals and clinics as clusters is rooted in their pivotal role in healthcare delivery, enabling the implementation and evaluation of interventions to enhance patient outcomes (26, 27, 30–34, 36–47, 49, 50, 52–71, 73–75, 77–81, 83–89, 91–96, 98–102, 104, 105, 107, 109).

Some studies focused on specific units within hospitals or clinics to target interventions at a more granular level. Primary care facilities were often chosen as clusters in low-resource settings (37, 52, 55, 56, 62, 70, 91, 92, 101, 108).

Clusters defined by geographical or administrative boundaries were used to capture broader population-based interventions, particularly in rural or resource-constrained areas. Administrative units such as districts and wards allowed for evaluating public health interventions across larger populations (26–28, 30, 33, 34, 36, 38, 42, 47, 51, 52, 62, 69, 71–73, 75–79, 84–86, 91, 92, 94–97, 99, 101, 104, 108, 109).

In more localized studies, clusters were often defined at the village or community level to capture the effects of interventions on specific populations (28, 29, 39, 48, 54, 72, 78, 81, 103, 106, 108).

Some studies utilized educational institutions as clusters to implement and evaluate public health interventions for children and adolescents. Schools served as effective clusters for interventions focused on health education, disease prevention, and other child-focused outcomes (35, 110).

Religious institutions were used as clusters in studies where community engagement and trust were crucial for the success of the intervention. Churches were selected as clusters in studies that leveraged community trust and leadership to deliver health interventions (29, 48).

In some studies, social groups or communities defined clusters to target interventions at a more personal and community-driven level. Clusters based on social networks allowed for the examination of interventions within the natural groupings of individuals (28, 29, 35, 48, 51, 72, 76, 78, 81, 82, 90, 97, 103, 106, 108, 110). Community-level clusters enabled the study of interventions that required collective participation or were community-driven. Some studies employed clusters that spanned multiple settings, such as combining health facilities with community or household interventions, reflecting the complexity of specific public health interventions. Studies that integrated health facility-based interventions with community outreach often used mixed-setting clusters (33–36, 38, 48, 54, 61, 67, 72, 76, 78, 80, 92, 99, 103, 106–108).

### Nature of intervention

**Figure 5:**
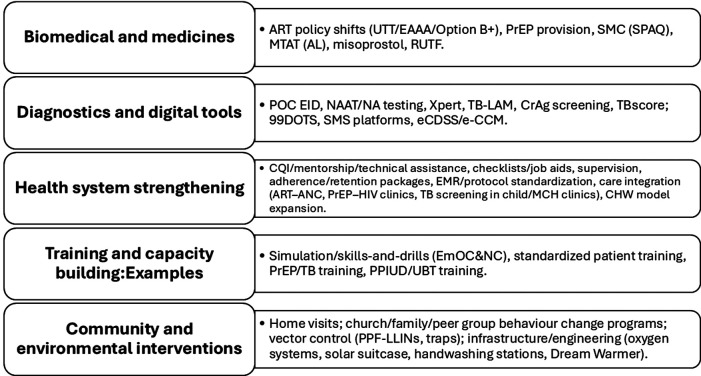
Categories of interventions evaluated using stepped-wedge designs. This figure categorises interventions evaluated in the included studies into five broad groups: biomedical and medicines; diagnostics and digital tools; health system strengthening; training and capacity building; and community and environmental interventions. The wide range of intervention types demonstrates the adaptability of stepped-wedge designs across clinical, health system, and community-based interventions.

Figure 5 shows the nature of interventions used for SWD identified in the included studies. Several studies focused on implementing health technologies and digital solutions to improve healthcare delivery and patient outcomes (26, 27, 32, 54, 62, 79, 85, 90, 91, 94, 98, 101, 104, 105). These interventions often involved using digital tools or devices designed to enhance the accuracy, efficiency, and accessibility of healthcare services. For example, a study utilized a 99DOTS-based TB treatment supervision strategy that utilizes a digital adherence tool where patients call toll-free numbers hidden in blister packs to confirm medication adherence (26). The intervention was supported by automated messaging systems and health worker monitoring to improve treatment adherence. The integration of point-of-care diagnostic tools was another common theme (41, 45, 68, 101). Emma Sacks (2020) implemented routine POC early infant diagnosis (EID) of HIV to provide timely on-site testing for infants, improving diagnosis and treatment initiation (45). Similarly, Eduard J. Sanders (2021) used point-of-care HIV-1 nucleic acid testing (NAAT) to detect acute HIV infections in symptomatic adults (41). Scott Dryden-Peterson (2015) introduced an automated SMS-based CD4 result platform that wirelessly distributed CD4 results to clinics, streamlining the process of HIV patient management in antenatal clinics (98).

Many of the interventions involved distributing and administering pharmaceutical treatments to prevent or manage diseases (28, 34, 36, 38–40, 42, 61, 90, 91, 93, 96, 98). Badara Cisse (2016) and J. L. Ndiaye (2016) implemented SMC with sulfadoxine-pyrimethamine (SP) plus amodiaquine (AQ) for children under ten years during the malaria transmission season, reducing the incidence of clinical malaria (34, 61). Dorothy Thomas (2022) and Elizabeth M. Irungu (2021) integrated PrEP delivery into existing HIV care services, offering it to HIV-negative partners of serodifferent couples, alongside ongoing technical assistance and training for healthcare providers (40, 43). The integration of ART services was a crucial focus in studies such as Elaine J. Abrams (2019), which transitioned from CD4+ guided ART eligibility to universal ART for all HIV-positive pregnant and breastfeeding women (42), and Osondu Ogbuoji (2019), which implemented the Early Access to Antiretroviral Therapy for All (EAAA) strategy in public-sector clinics (87).

Many studies involved interventions centered around training healthcare workers or providing health education to patients and communities (29, 32, 33, 48, 49, 62, 70, 78, 81, 86, 88, 89). Anne Antonia Cornelia van Tetering (2021) focused on a one-day simulation-based acute obstetric training for healthcare providers, emphasizing medical technical skills and teamwork in managing obstetric emergencies (32). Pascal Geldsetzer (2020) provided training for healthcare providers on PrEP delivery as part of HIV prevention efforts (89). Ali M. Giusto (2017) implemented a family- and church-based intervention aimed at preventing HIV risk behaviors among adolescents by strengthening family relationships through community-based education (29). Anne Cockcroft (2022) provided universal home visits by trained health visitors to educate pregnant women and their spouses on local maternal and child health risk factors (33).

Behavioral and psychosocial interventions aimed at altering behavior and improving mental health outcomes were a crucial focus in several studies (33, 48, 49, 78, 80, 81, 88, 93, 102, 105, 107, 110). Margaret W. Gichane (2020) introduced the Women's Health CoOp (WHC), a gender-focused intervention combining HIV and alcohol risk reduction with behavioral skills training, focusing on sexual empowerment and communication skills (80). Wendee M. Wechsberg (2021) also utilized the WHC intervention in a different context, aiming to reduce HIV-related risks among women who use alcohol and drugs (107). Stella-Maria Paddick (2017) implemented Cognitive Stimulation Therapy (CST), a psychosocial intervention to improve cognitive function in individuals with dementia (102). Eva Wodeya Wanyenze (2022) focused on providing orientation sessions for birth companions to support labor through emotional and physical support techniques (47).

Some interventions aimed to strengthen healthcare infrastructure and systems to improve service delivery and patient outcomes (27, 49, 52, 54, 56–58, 60, 62, 65, 66, 68, 70, 72, 74, 77, 79, 84–88, 90, 91, 93–96). Hamish R.Graham (2019, 2021) focused on implementing improved oxygen systems in hospitals, including the introduction of pulse oximetry and full oxygen systems for children in Nigeria, aiming to reduce mortality from hypoxemia (57, 58). Wilbroad Mutale (2023) introduced the Better Health through Mentoring and Assessment (BHOMA) project, which involved intensive clinical training, quality improvement measures, and improved community outreach to enhance healthcare service delivery at the primary care level (108).

Integration care models were also implemented in some studies combining multiple health services or interventions into a cohesive approach (44, 77, 83, 97). Emily B. Wroe (2021) expanded an existing community health worker (CHW) program for HIV and TB into a broader household-based model that included non-communicable diseases (NCDs), malnutrition screening, and family planning services (44). Leigh Senderowicz (2022) focused on integrating postpartum intrauterine device (PPIUD) insertion into routine antenatal family planning counselling (77). Monica P. Patel (2005) provided supplemental feeding with Ready-to-Use Therapeutic Food (RUTF) for children at risk of malnutrition, integrating nutritional support into routine maternal and child health services (83). Samson Winani (2007) combined the use of clean delivery kits with health education on the "six cleans" to prevent maternal and newborn infections (97).

### Randomization

Simple randomization, often using computer-generated sequences, was a standard method employed to determine the order in which clusters received the intervention (26, 30, 32, 35, 38, 47, 58, 65). This approach ensures that each cluster has an equal chance of being assigned to any given sequence, reducing selection bias. Adithya Cattamanchi (2021) used a simple, unrestricted two-stage process where health facilities were randomly assigned to blocks and the order of intervention was determined through a random process (26). Similarly, Ryan R. Thompson (2022) used a random process to determine which clinics would receive the 99DOTS intervention each month (94). In the study by Hamish R.Graham (2021), hospitals were allocated to receive an oxygen system based on a computer-generated random sequence, ensuring a phased introduction of the intervention (58). This method was similarly employed by Eva Wodeya Wanyenze (2023), where a simple random technique determined the order of hospital crossover to the intervention period (47).

**Figure 6:**
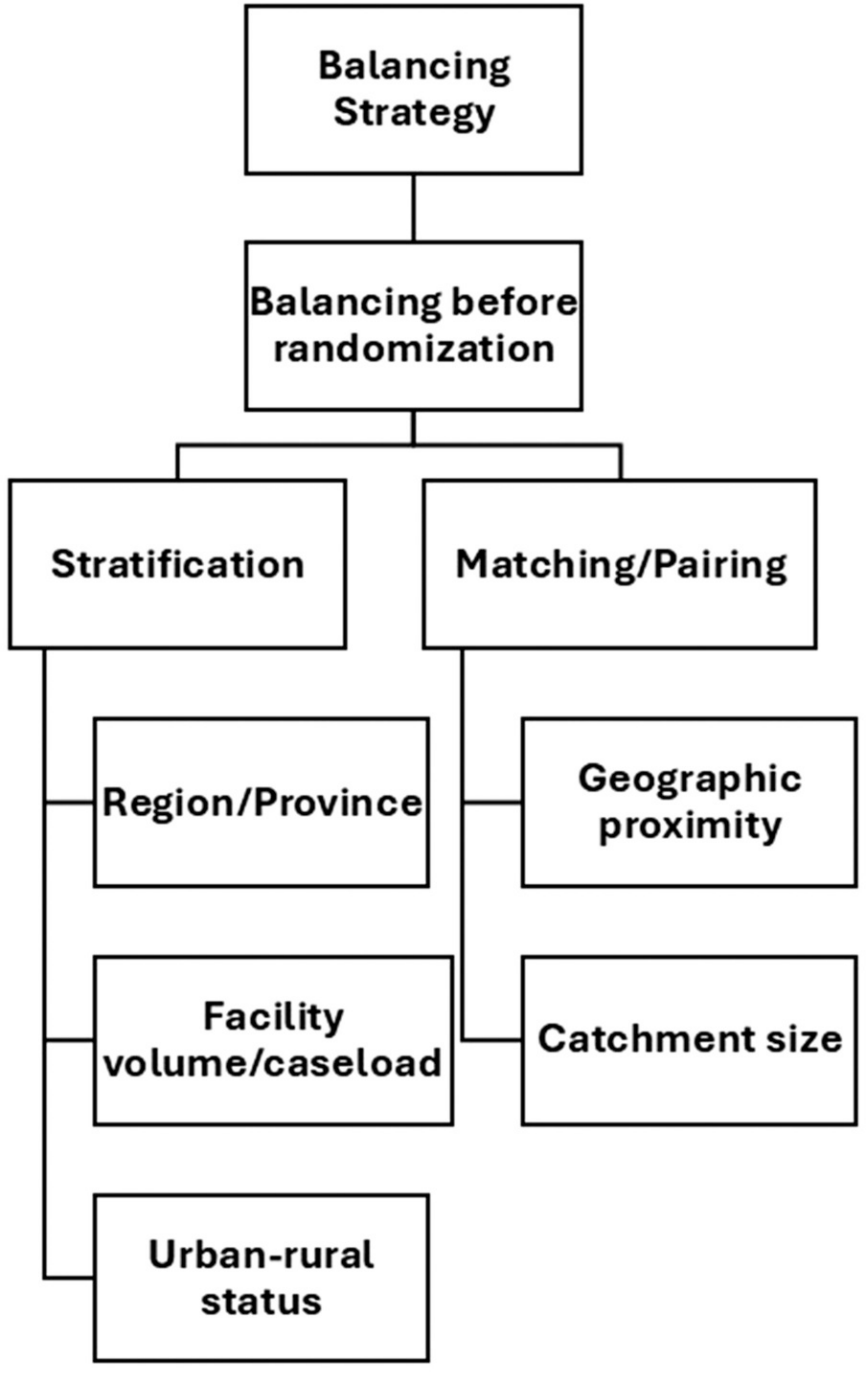
Strategies used to balance clusters prior to randomisation. This diagram shows balancing strategies applied before randomisation, including stratification (by region/province, facility volume/caseload, urban–rural status) and matching/pairing (by geographic proximity and catchment size). These approaches were used to improve baseline comparability and reduce imbalance across clusters.

Stratified randomization was used in several studies to ensure balance across key variables such as geographic location, facility size, or patient volume before random assignment (40, 43, 60, 64–66, 70, 71, 88, 99, 105). This technique helps control potential confounding factors by ensuring that these variables are equally distributed across the intervention groups. Janina Isabel Steinert (2021) grouped health facilities into pairs based on geographic proximity and catchment size before randomizing them into different sequences for intervention rollout (65). This approach ensured that facilities with similar characteristics were evenly distributed across the study arms. Elizabeth M. Irungu (2021) utilized stratification by region before randomly assigning clinics to the order in which they would start receiving the intervention (43). This method was also used by Pamela K. Kohler, who stratified randomization based on region and facility size to ensure a balanced distribution of intervention timing across different facility types (88). Block randomization was employed to ensure that equal numbers of clusters received the intervention at each stage, particularly in studies with phased rollouts. This method is useful in maintaining balance throughout the intervention period. Dorothy Thomas (2022) randomized facilities into groups of four, with each group beginning the intervention six months apart (40). This ensured that the implementation was evenly distributed over time while controlling for potential confounders related to the timing of intervention delivery. Katherine R. Sabourin (2022) used block randomization to balance the number of urban and rural sites, ensuring that the timing of intervention rollout was similar across different settings (70). This method ensured that both urban and rural sites had comparable exposure to the intervention phases (see Figure 6).

In some studies, randomization was performed using a lottery or public draw method, which added an element of transparency and community involvement in the randomization process (29, 43, 78, 106). This method was particularly used in community-based interventions. Ali M. Giusto (2017) employed a lottery system conducted by community leaders to randomly select churches for participation (29). This approach was similarly used by Lily C. Kumbani, where communities were randomly assigned to intervention sequences by drawing names from a basket (78). In the study by Elizabeth M. Irungu (2021), randomization was conducted at stakeholder events where facility representatives picked numbered opaque balls from a bag, determining the sequence in which their facilities would receive the intervention. This method increased transparency and stakeholder engagement in the research process (43).

**Figure 7:**
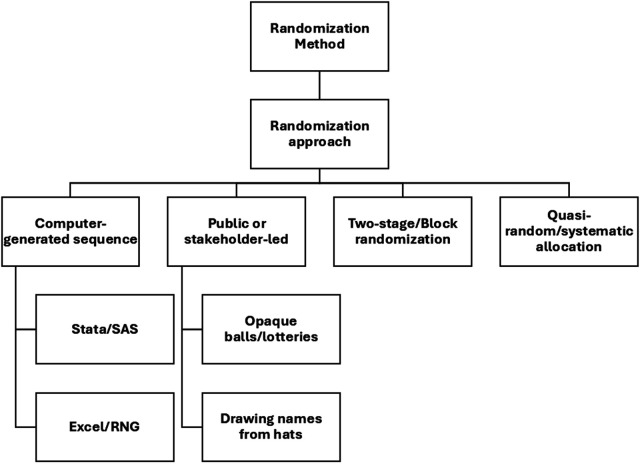
Randomisation methods used in stepped-wedge studies. This figure summarises the randomisation approaches used across studies, including computer-generated sequences (e.g., Stata/SAS, Excel random number generators), public or stakeholder-led methods (e.g., lotteries, drawing names), two-stage or block randomisation, and quasi-random or systematic allocation. The diversity reflects varying methodological and contextual constraints.

Some studies utilized systematic or quasi-randomized approaches when true randomization was not feasible, often due to logistical constraints or the need to align with existing organizational structure (35, 83, 106). Calum Davey (2015) employed a quasi-randomized method where schools were systematically allocated into three groups using an Excel spreadsheet, with each group receiving the intervention at different times (35). This approach was necessary due to the school system's structure and logistical considerations. Tobias Homan (2016) used the traveling salesman algorithm to assign households to clusters, ensuring an efficient rollout of the intervention while maintaining a randomized element (106). This innovative approach was particularly suited for interventions requiring a geographically coherent implementation plan (see Figure 7).

**Figure 8:**
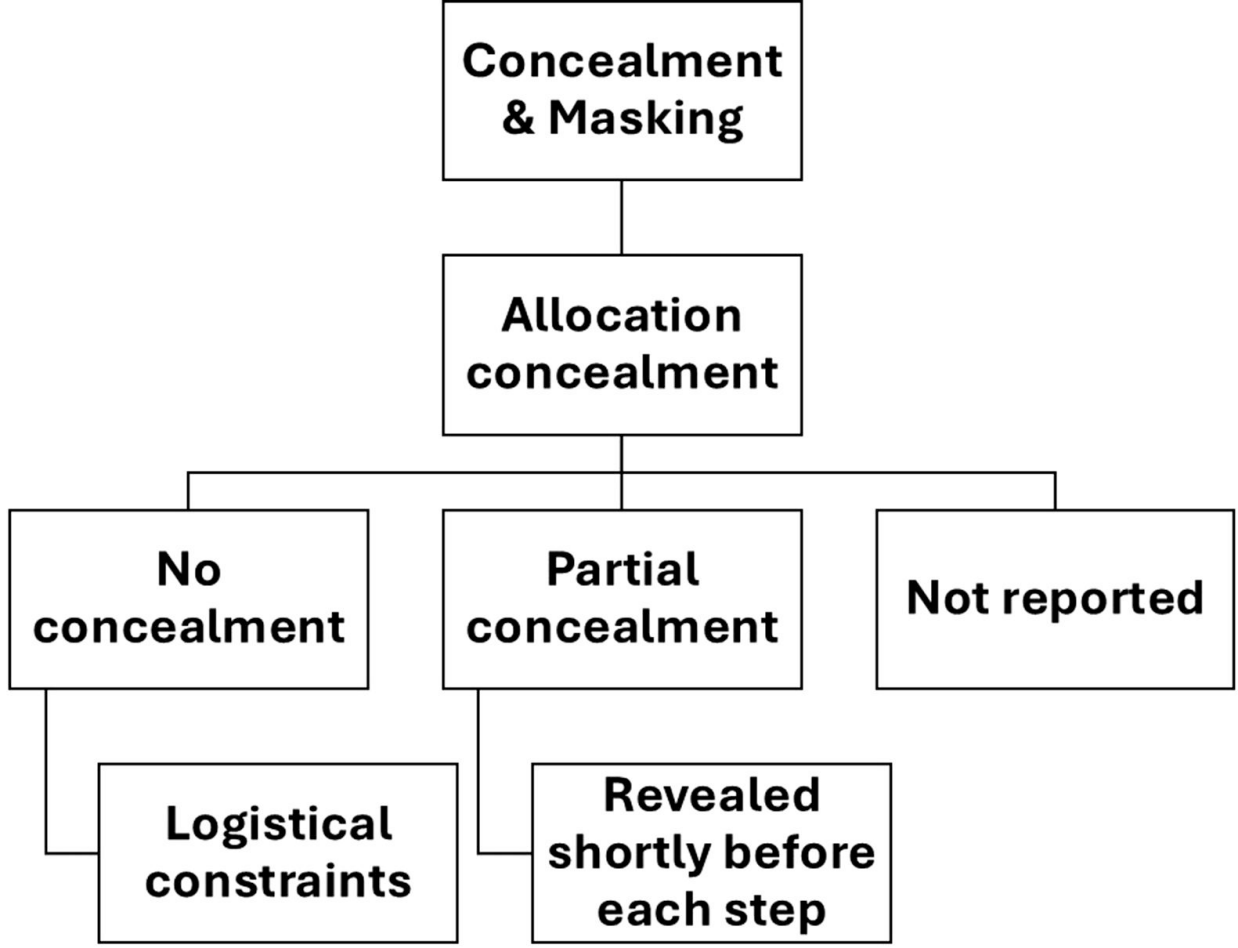
Allocation concealment and masking practices. This schematic presents how allocation concealment was handled in included studies, categorised as no concealment, partial concealment (e.g., allocation revealed shortly before each step), or not reported. Logistical constraints commonly limited full concealment, reflecting real-world implementation challenges in stepped-wedge trials.

In several studies, randomization was combined with masking to reduce bias during the intervention phase (43, 56). Masking ensures that participants and sometimes even the investigators are unaware of the sequence of intervention, which can help prevent conscious or unconscious influences on the study outcomes. H. Manisha Yapa (2020) randomized clusters to receive the intervention on pre-specified dates, with investigators and clusters being blinded to the randomization sequence until two weeks before each step (56). This method was also applied in Janina Isabel Steinert (2020), where the randomization sequence was concealed to minimize potential biases (66) (see Figure 8).

### Reasons for SWD

One of the primary reasons for choosing the SWD was the ethical imperative to ensure that all study participants eventually received the intervention, especially when the intervention was expected to be beneficial (44, 46, 47, 52, 55, 57, 69, 96, 97, 106, 110). Emily B Wroe (2021) and Hamish R. Graham (2021) selected the SWD to ensure that all clusters eventually received the potentially beneficial interventions while allowing for rigorous evaluation (44, 58). Wit Wichaidit (2019) also used SWD to avoid the ethical dilemma of withholding a beneficial intervention (handwashing stations) from some schools, emphasizing that the intervention posed no harm and provided clear benefits (110). Samson Winani (2007) and Wendee M. Wechsberg (2021) employed the SWD to address ethical issues associated with traditional cluster-randomized trials where control clusters might not receive an efficacious intervention (97, 107). The SWD allowed for the gradual introduction of the intervention across all clusters, ensuring ethical treatment of all participants.

The SWD was often chosen to accommodate logistical and operational challenges, particularly in studies where simultaneous implementation across all clusters was impractical (26, 30, 33, 34, 36, 37, 40, 43, 45, 49, 51, 52, 58, 61, 62, 65, 66, 68, 81, 86, 91, 94, 101, 110). Dorothy Thomas (2022) and Slawa Rokicki (2021) selected the SWD to manage logistical constraints, allowing for a phased rollout of the intervention (40, 100). This design was practical in settings where resources were limited, enabling a more manageable and effective implementation process (41, 45, 50, 51, 62, 77, 83, 91, 100–102). Sophie Sarrassat (2021) and Tobias Homan (2016) used the SWD to facilitate the gradual rollout of interventions, which was more practical for implementing agencies and allowed for the effective management of resources (101). Jackie Sturt (2023) also cited capacity constraints and the need for phased implementation during the COVID-19 pandemic as reasons for choosing the SWD (62).

Quite a number of these studies selected SWD to account for time-related confounders, ensuring that temporal trends did not bias the results. Calum Davey (2015) and Badara Cisse (2016) employed the SWD to adjust for time effects, given evolving programs and external conditions that could influence the study outcomes (34, 35). By implementing the intervention in a phased manner, these studies were able to control for potential confounders related to the timing of the intervention. CE Boeke (2020) and Clara A. Agutu (2022) chose the SWD to allow for a systematic rollout of the intervention across all facilities over time, controlling for time-related confounders while ensuring that each facility eventually served as its own control (36, 37).

The SWD was frequently used to ensure that all clusters or study sites eventually received the intervention, particularly in public health studies where broad coverage was essential (28, 33–35, 37, 43–50, 52, 54–58, 60–62, 68, 70, 71, 74, 77–79, 81, 82, 85–90, 92–94, 96, 98, 99, 103, 105, 106, 108, 109). Elizabeth M. Irungu (2021) and David B. Meya (2019) opted for the SWD to facilitate a staggered approach to implementation, ensuring that all clinics or health facilities eventually received the intervention (39, 43). This was particularly important in studies integrated into broader national or regional health programs. Emma Sacks (2020) and Kenneth K. Mugwanya (2023) selected the SWD to maximize the impact of the intervention by ensuring its systematic rollout across all participating sites, enabling a thorough evaluation while maintaining operational efficiency (45, 71).

Practical considerations, including the need for a flexible and adaptive study design, were key drivers for selecting the SWD in many studies. Janina Isabel Steinert (2021) and James T. Pfeiffer (2017) used the SWD to accommodate logistical and practical constraints over extended study periods (64, 65). The phased implementation allowed for adjustments based on real-world conditions, making the design more adaptable and feasible. Geofrey Musinguzi (2020) and F. Mosha (2005) highlighted the SWD's efficiency in resource-constrained settings, where a staggered rollout allowed for better management of limited resources while still providing a rigorous evaluation of the intervention's effectiveness (49, 51).

The SWD was chosen in some studies because it allowed the intervention to be integrated into existing health programs, facilitating a smoother transition and broader acceptance. Andrew F. Auld (2020) and Tefera Agizew (2019) selected the SWD because the intervention was part of a national rollout (31, 104). The design allowed for the phased implementation of the intervention across multiple sites, ensuring alignment with existing health initiatives while enabling a robust evaluation. Alexandra E. Ridout (2023) and Holly A. Anger used the SWD to embed the intervention into routine clinical pathways, ensuring that all study sites could eventually incorporate the intervention into their standard practices while maintaining a focus on evaluating its effectiveness over time (27, 60).

### Number of steps

**Figure 9:**
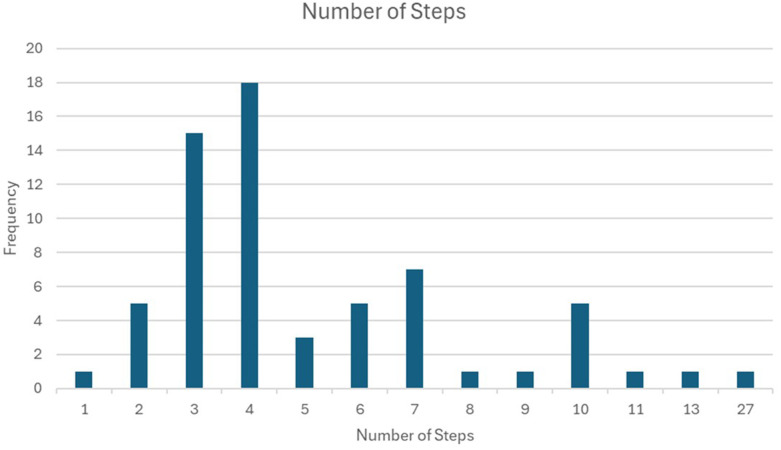
Distribution of number of steps across included studies. This histogram shows the frequency distribution of the number of steps used in stepped-wedge designs. Most studies used between 3 and 7 steps, with fewer studies employing very short (1–2 steps) or highly granular designs (≥10 steps), reflecting trade-offs between feasibility and temporal resolution.

The number of steps in a SWD is crucial as it influences the study's duration, complexity, and ability to detect time-related changes. The number of steps in the reviewed studies varied significantly, reflecting the flexibility of the SWD to adapt to different research needs and contexts. The fewest steps used in an SWD study was 1, as seen in Stella-Maria Paddick (2017) (102). This study likely opted for a single step due to resource limitations or the simplicity of the intervention being tested. The highest number of steps observed was 27, used in Tobias Homan (2016) (106). This extensive number of steps was likely necessary to facilitate a highly granular and phased rollout of the intervention, perhaps due to logistical challenges or the need to closely monitor the impact at multiple stages (see Figure 9).

Several studies opted for a moderate number of steps, with 3 to 4 steps being the most frequently chosen configuration. This range provides a balance between logistical feasibility and the ability to rigorously evaluate the intervention over time.

A significant number of studies employed 3 steps, such as in Andrew F. Auld (2020), Dorothy Thomas (2022), and Renee Heffron (2022) (31, 40, 93). This choice often reflects a phased approach where the intervention can be rolled out in manageable increments while maintaining sufficient control over time-related variables.

Another common choice was 4 steps, seen in studies like Emma Sacks (2020), Anne Cockcroft (2022), and Shaukat Khan (2020) (33, 45, 99). This configuration allows for a more detailed evaluation compared to fewer steps while still being relatively straightforward to manage.

The rationale behind choosing a specific number of steps is often related to the intervention's logistical demands, the study's complexity, and the need to control for temporal effects. In studies with extensive logistical demands or resource constraints, fewer steps were often chosen to simplify the implementation process. For example, Alexandra E. Ridout (2023) and Calum Davey (2015) both utilized two steps to streamline the rollout of their interventions (27, 35).

Studies requiring detailed monitoring or involving complex interventions tended to use more steps. For instance, Hamish R.Graham (2019) used 12 steps to introduce an oxygen system in hospitals, which allowed for careful monitoring and adjustment at each stage of the rollout (57).

Some studies balanced complexity and feasibility by choosing a moderate number of steps. Anne Antonia Cornelia van Tetering (2021) used 7 steps to implement a training program for healthcare workers, which allowed for phased implementation and comprehensive evaluation without overwhelming the study team (32).

Several studies did not explicitly define the number of steps or used a variable number of steps based on the study's progression.

Studies like David B. Meya (2019) and Eduard J. Sanders (2021) involved multiple steps without a fixed number, with facilities transitioning from control to intervention phases at different intervals (39, 41). This approach provided flexibility to adapt the study design based on real-time developments and operational constraints.

**Figure 10:**
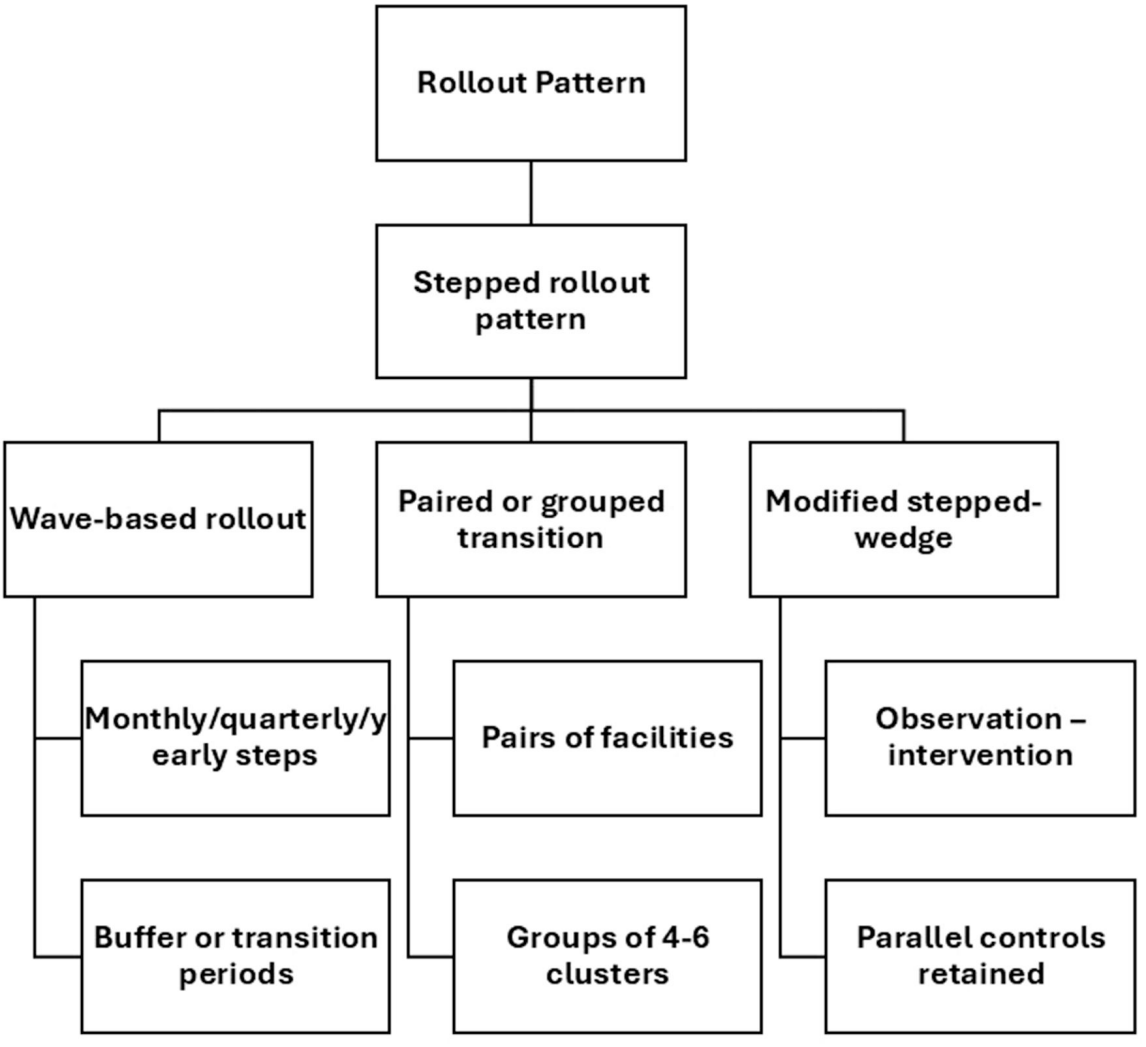
Distribution of the number of clusters per study. This plot displays the frequency distribution of the number of clusters used across included studies. Most studies involved fewer than 30 clusters, while a small number of large-scale studies used substantially higher numbers of clusters, indicating variability in scale and design complexity across stepped-wedge trials in SSA.

In some cases, the exact number of steps was not clearly specified in the study documentation, as seen in CE Boeke (2020) and Noah Kiwanuka (2023) (36, 85). This could indicate a more fluid approach to study design, where the number of steps was determined by external factors such as funding, participant availability, or logistical support (see Figure 10).

### Number of clusters

The number of clusters in the studies varied widely, reflecting the SWD's flexibility in accommodating different research contexts and logistical constraints.

**Figure 11:**
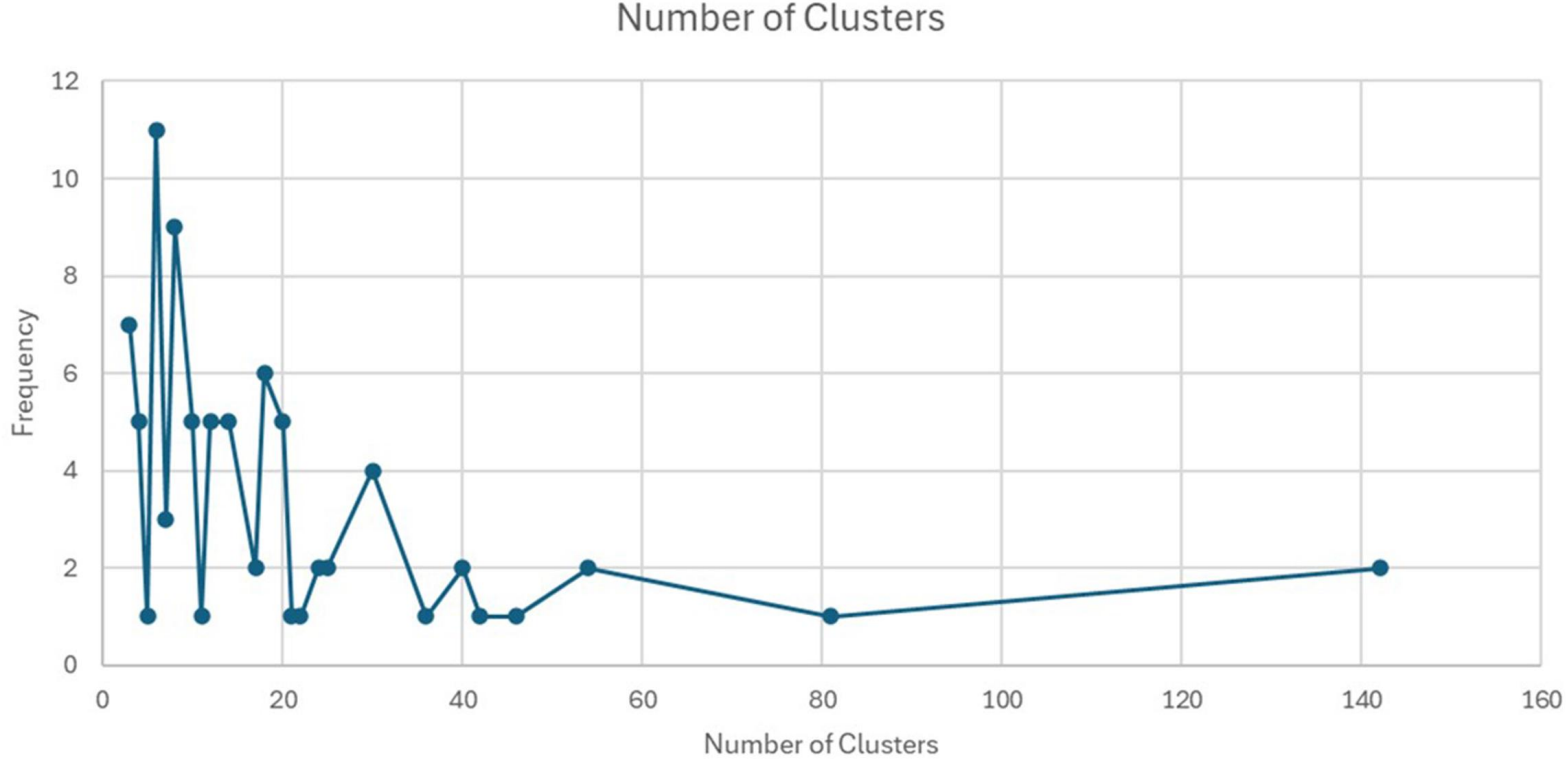
Rollout patterns used in stepped-wedge designs. This schematic illustrates common stepped-wedge rollout patterns, including wave-based rollouts with regular time intervals, paired or grouped transitions of clusters, and modified stepped-wedge designs retaining parallel controls or incorporating observation–intervention phases. These variations reflect adaptations of the stepped-wedge design to operational and logistical constraints.

The study with the fewest clusters used only 3 clusters, as seen in Johanna Åhsberg (2023) and Jeffrey Rewley (2020) (67, 68). These studies likely involved small-scale interventions or were conducted in highly controlled environments where limited clusters were sufficient for achieving the study objectives. The study with the most clusters included 142 clusters, as seen in Pren Naidoo (2017) and Nynke van den Broek (2019) (86, 90). These studies were typically large-scale public health interventions that required a significant number of clusters to cover diverse populations and settings, allowing for a robust evaluation of the intervention across various contexts (see Figure 11).

Several studies employed a moderate number of clusters, with 6 to 10 clusters being the most frequently chosen configuration. This range provided a balance between statistical power and logistical feasibility.

A significant number of studies used 6 clusters, such as Leigh Senderowiez (2022), Emily B Wroe (2021), and Frauke Rudolf (2021) (44, 50, 77). This number of clusters allowed for a manageable study size while still providing enough variation to detect differences across clusters.

Ten clusters were also commonly used, as seen in studies like F. Mosha (2005) and Ana Pilar Betrán (2018) (30, 49). This configuration offered a good balance between complexity and the ability to generalize the findings across different settings.

The rationale behind choosing the number of clusters is often related to the study's logistical constraints, the need for statistical power, and the geographical spread of the intervention.

Studies often selected a moderate number of clusters based on logistical feasibility. For example, Badara Cisse (2016) used 54 clusters (health posts) due to the need to implement a phased intervention in different regions over time (34). Similarly, Katherine R. Sabourin (2022) used 8 clusters to ensure a balanced implementation across both rural and urban healthcare settings (70).

The number of clusters was frequently determined by the need to achieve sufficient statistical power. Andrew F. Auld (2020) used 21 clusters to ensure adequate power to detect a reduction in ART mortality rates (31), while Anne Antonia Cornelia van Tetering (2021) used 7 clusters to provide enough data points for a statistically robust analysis (32).

In some studies, the number of clusters was influenced by the geographical distribution of the intervention sites. Leigh Senderowicz (2022) used 6 clusters across different regions in Tanzania to ensure that the intervention could be evaluated across diverse healthcare settings (77).

A few studies required an exceptionally high number of clusters to accommodate large-scale interventions or to account for a wide range of settings.

Nynke van den Broek (2019) involved 127 clusters across 11 districts in South Africa, reflecting the study's national scope and the need to cover a broad range of healthcare facilities (86). Pren Naidoo (2017) similarly used 142 clusters to evaluate a tuberculosis diagnostic intervention across multiple primary healthcare clinics, demonstrating the scale required to assess such a widespread public health intervention (90).

### Time between steps

The time between steps in the SWD studies varied widely, reflecting the design's adaptability to different research needs and logistical constraints.

The shortest time between steps was two weeks, observed by Josee Uwamariya (2021) and Tefera Agizew (2019) (69, 104). This brief interval was likely chosen to allow for rapid rollout and assessment of the intervention in settings where timely results were critical.

The longest time between steps was 121 months (approximately 10 years), seen in Pascal Geldsetzer (2020) (89)This extended interval was likely due to the intervention's long-term nature and the need to observe outcomes over a prolonged period.

Several studies employed a moderate time interval between steps, with 1 to 6 months being the most frequently chosen configuration (26–28, 30–32, 36, 38–46, 48, 49, 52, 55–58, 62–64, 67, 70, 73, 74, 76, 80–82, 86, 87, 91, 92, 94, 96, 98–101, 105). This range provides a balance between allowing sufficient time for the intervention to take effect and maintaining the momentum of the study.

A significant number of studies used 1 month between steps, such as William P. Killam (2010), Lea Multerer (2021), and Ryan R. Thompson (2022) (94, 109). This relatively short interval allowed for a quick assessment of the intervention's impact while keeping the study timeline manageable.

Six months was another common choice, as seen in Adithya Cattamanchi (2021), Dorothy Thomas (2022), and Wendee M. Wechsberg (2021) (26, 40, 107). This duration provided ample time for the intervention to be fully implemented and for its effects to be observed before moving to the next cluster.

The rationale behind selecting specific time intervals between steps is often related to the nature of the intervention, the study's logistical constraints, and the need to control for time-related confounders.

The complexity and expected impact of the intervention often dictated the time between steps. For instance, Eduard J. Sanders (2021) used a 6-month interval to allow sufficient time for the intervention (HIV prevention services) to take hold and produce measurable outcomes (41). In contrast, Holly A. Anger (2020) used just 1 week between steps, likely due to the rapid nature of the intervention and the need for quick implementation (60).

Logistical considerations, such as the availability of resources and the need to coordinate with multiple sites, influenced the time between steps. Hamish R.Graham (2019) chose 4 months between steps to allow for the installation of oxygen systems in hospitals, which required careful planning and execution (57).

In studies where time-related confounders were a concern, longer intervals were chosen to ensure that any effects observed were attributable to the intervention rather than external factors. Anne Cockcroft (2022) and Badara Cisse (2016) both used 1 year between steps to account for seasonal variations and other time-related factors that could influence the outcomes (33, 34).

Some studies did not have a fixed time between steps or employed variable intervals based on the study's specific needs.

Wit Wichaidit (2019) used variable intervals ranging from 3 to 24 weeks depending on the group, reflecting the need to adapt the study timeline to different settings or phases of the intervention (110). Katherine R. Sabourin (2022) used 7–8 weeks between steps, providing some flexibility in the study design (70).

In certain studies, the time between steps was not explicitly defined, allowing for flexibility in the study design. Lily D. Yan (2017) and Margaret W. Gichane (2020) had undefined intervals, likely due to the need to adapt to changing circumstances or logistical challenges during the study (34, 79).

### Sample size calculation

The sample size calculation in SWD studies varied significantly, reflecting the diversity in study designs, outcomes of interest, and the complexity of the interventions.

Some studies provided detailed sample size calculations, incorporating specific assumptions about expected outcomes, intracluster correlation coefficients (ICC), and power. For example, Adithya Cattamanchi (2021) calculated a sample size with 89% power to detect a 10% increase in treatment success based on 18 clusters and an ICC of 0.001 (26). Similarly, Alexandra E. Ridout (2023) aimed for nearly 100% power, factoring in within-period intra-cluster correlations and cluster autocorrelation coefficients (27).

Some studies had to account for multiple outcomes in their sample size calculations, making the process more complex. Anne Antonia Cornelia van Tetering (2021) calculated the sample size based on the combined maternal and neonatal mortality ratios, with adjustments for cluster design effects and varying cluster sizes (32).

In contrast, several studies did not provide detailed sample size calculations, either due to the complexity of the design or because the primary focus was on feasibility rather than precise power estimates. For instance, Dorothy Thomas (2022) and Eduard J. Sanders (2021) did not detail their sample size calculations, possibly due to challenges in estimating the required sample size in real-world settings (40, 41).

The number of clusters expected effect size, ICC, and study design were key factors influencing sample size calculations in SWD studies.

The number of clusters and the ICC were critical factors in sample size determination. For example, Ana Pilar Betrán (2018) assumed a baseline frequency of 30% for health practices and calculated the sample size with an ICC of 0.05 (30). Similarly, Andrew F. Auld (2020) used a coefficient of variation of 0.2 and a 24-month enrollment period to estimate the required sample size for detecting a reduction in ART mortality rates (31).

The desired effect size and the power to detect this effect significantly impacted the sample size. For instance, Emma Sacks (2020) aimed to detect a 50% increase in caregivers receiving results, using a design effect of 2 across 18 clusters (45). Geofrey Musinguzi (2020) calculated a sample size to detect changes in cardiovascular disease (CVD) risk factors with adequate power (51).

Calculating sample size for SWD studies is particularly challenging due to several inherent complexities:

The SWD's phased implementation and the need to account for within-cluster and between-cluster variations make sample size calculations more complex than traditional designs. This complexity often requires sophisticated statistical methods and software, as noted in Frauke Rudolf (2021), who used the "steppedwedge" function in Stata for calculations (50).

Estimating the ICC is crucial but challenging, as it directly affects the power and sample size required. An inaccurate ICC estimate can lead to underpowered studies or unnecessarily large sample sizes, as highlighted in Sophie Sarrassat (2021), who assumed a design effect 2 and an ICC of 0.3 for sample size estimation.

In real-world settings, the availability of data, logistical constraints, and variability in cluster sizes can complicate sample size calculations (101). Wilbroad Mutale (2023) had to adjust the sample size during the study due to lower-than-expected mortality rates, illustrating the need for flexibility in sample size planning.

Many studies did not provide detailed sample size calculations, often due to the abovementioned challenges. Sometimes, sample sizes were based on pragmatic considerations rather than formal calculations.

Studies like Dorothy Thomas (2022) and Eduard J. Sanders (2021) likely prioritized feasibility and real-world applicability over precise sample size calculations, focusing on enrolling as many participants as possible within the available resources (40, 41).

Some studies may have based their sample size on previous similar studies or pilot data rather than conducting detailed new calculations. This approach was seen in studies like Lily C. Kumbani, where simulations were used to estimate the sample size needed to ensure 80% power (78).

### Primary outcome

Many SWD studies focused on health outcomes and managing specific diseases, reflecting the importance of improving clinical practice and patient health in various settings.

A significant number of studies targeted outcomes related to HIV and TB. For example, Adithya Cattamanchi (2021) focused on the proportion of patients with treatment success for TB, while Dorothy Thomas (2022) measured HIV viral suppression among partners living with HIV (26, 40, 41). Similarly, Eduard J. Sanders (2021) aimed to assess the number of new HIV diagnoses, combining chronic and acute cases.

Malaria-related outcomes were also common, as seen in Alfred B Tiono (2014), where the incidence of clinical episodes of malaria among children was a primary outcome (28). Lea Multerer (2021) measured clinical malaria defined as fever plus a positive RDT result for Plasmodium falciparum (76).

Alexandra E. Ridout (2023) evaluated maternal mortality, major maternal morbidity, and fetal losses as a composite primary outcome (27). Anne Cockcroft (2022) assessed male spouses' knowledge and attitudes related to maternal and child health, including the knowledge of danger signs during pregnancy and childbirth (33).

Several studies aimed to assess changes in behavior, psychosocial factors, and knowledge as primary outcomes, often in the context of public health interventions.

Ali M. Giusto (2017) explored the associations between fathers' and sons' sex-related beliefs and behaviors, moderated by parenting characteristics (29). Wit Wichaidit (2019) focused on handwashing behavior among schoolchildren, assessing the availability of handwashing materials and observed handwashing behavior after toilet use (110).

Eve S. Puffer (2016) measured family communication, HIV risk knowledge, and self-efficacy as primary outcomes (48). Stephanie R. Psaki (2022) targeted the increased use of HIV prevention and treatment services, reduction in sexual risk behaviors, and reduction in sexual and gender-based violence as primary outcomes (103).

A number of studies focused on improving clinical and diagnostic processes, with primary outcomes centered around their effectiveness.

Frauke Rudolf (2021) measured the diagnostic yield for smear-positive TB as the primary outcome, with secondary outcomes including treatment success and overall mortality (50). Priya B. Shete (2023) focused on the number of people initiating treatment for microbiologically confirmed TB within 2 weeks of presenting to the health center (92).

Hamish R. Graham (2019, 2021) evaluated child mortality and mortality from acute lower respiratory infections (ALRI) as primary outcomes, reflecting the impact of interventions like oxygen systems in hospitals.

Some studies aimed to assess the impact of interventions on patients' quality of life and satisfaction with healthcare services (57, 58).

Stella-Maria Paddick (2017) used the Brief WHO Quality of Life (WHOQOL-Bref) scale to assess changes in quality of life as the primary outcome. This focus highlights the importance of evaluating not just clinical outcomes but also the broader impacts of healthcare interventions on patient well-being (102).

Osondu Ogbuoji (2019) measured patient satisfaction as the primary outcome, including overall satisfaction and satisfaction with specific domains such as wait time, consultation time, involvement in treatment decisions, and respectful treatment (87).

Some studies focused on the performance of health systems and the effectiveness of service delivery as primary outcomes.

Geoffrey A. Jobson (2021) assessed the proportion of HIV-infected adults screened for TB at ART initiation and the proportion of adults retained in care as primary outcomes (52). Pamela K. Kohler measured youth retention in HIV care, particularly during early engagement, as a key outcome (88).

Janina Isabel Steinert (2021) evaluated total private healthcare expenditures as the primary outcome, assessing the financial impact of healthcare interventions on households (65). Wilbroad Mutale (2023) focused on age-standardized mortality among individuals to measure health system performance.

Several studies employed composite or multifaceted primary outcomes, reflecting the complexity of the interventions and the need to assess multiple dimensions of impact.

Alexandra E. Ridout (2023) used a composite rate of maternal mortality, significant maternal morbidity, and fetal losses as the primary outcome. This approach allowed for a comprehensive assessment of the intervention's impact on maternal health (27).

Margaret W. Gichane (2020) assessed the adoption, acceptability, appropriateness, cost, and fidelity of the intervention as primary outcomes, demonstrating the importance of evaluating multiple aspects of program implementation and effectiveness (80).

### Quality of reporting

The quality of reporting in cluster-randomized controlled trials (RCTs) utilizing the stepped wedge design was evaluated across Eighty-five study reports, using headings and subheadings adapted from the Consolidated Standards of Reporting Trials (CONSORT) (26–110) (see Supplementary File S3). Eighty-four studies specified the method used to allocate clusters to different intervention implementation periods, as indicated in the title and/or abstract (26–93, 95–110). Eighty-one studies provided a diagrammatic representation of the design (26–31, 33–39, 41–48, 50–70, 72–110), which was particularly helpful for understanding the general characteristics of the design and correctly classifying the studies as employing a stepped wedge design. The interventions were adequately described in eighty-four studies (26–78, 80–110).

Seventy-one studies reported sample size calculations (37, 44, 49, 52, 63, 66, 70, 71, 79, 91, 94, 97, 107, 109), though only Fifty-nine considered the design effect, including the intracluster correlation coefficient (26, 27, 29–36, 38, 39, 41–45, 48, 51, 53–62, 64, 65, 67, 68, 70, 72–74, 77–82, 84, 85, 88, 89, 92, 93, 96, 99–101, 103–106, 108, 109). Eighty studies described sequence generation (26–48, 50, 52–70, 72–82, 84–90, 92–110), while Forty-seven reported allocation concealment (27–31, 33, 36–39, 42, 43, 46, 47, 52–60, 62, 65, 69, 73, 74, 77–81, 85, 86, 88, 89, 97–99, 101, 102, 104, 106, 107, 109, 110). Additionally, sixty-one studies documented restricted randomization (27, 29–31, 33–35, 38, 39, 41–43, 45, 48, 50, 51, 54, 56–62, 64, 65, 67, 68, 70, 72–78, 80–85, 87–90, 92, 93, 95, 96, 98–106, 108, 109), and twenty-nine reported employing blinding (56–60, 62, 65, 69, 73, 74, 77–80, 97–99, 101, 102, 104, 106, 107, 109).

The use of intention-to-treat principles was reported in sixty-nine studies (26, 27, 29–31, 33–39, 41–43, 45, 47, 48, 50, 53–65, 67–70, 72–75, 77, 78, 80–90, 92–96, 98–106, 108, 109). A participant flow diagram was included in eighty-one studies (26–31, 33–48, 50–75, 77–93, 95–110), with all studies indicating the number of clusters and individuals included in the analysis (26–110). Baseline data and summaries of primary outcomes were provided in all the studies (26–110). Eighty-two studies included an effect size estimate (26–34, 36–48, 50–70, 72–110), while Eighty-three reported precision estimates (26–48, 50–70, 72–110). Forty-one studies provided information on adverse events (27, 28, 35, 36, 38, 39, 46, 48, 53, 55, 60–63, 66, 67, 69, 70, 74, 75, 78, 81, 84–86, 88, 90, 92, 93, 95–97, 99, 102–105, 107–110). Furthermore, all studies discussed the generalizability of the results and acknowledged their respective limitations (26–110).

## Discussion

This systematic review provides a detailed examination of the application and effectiveness of SWD in research across SSA. Our findings highlight a significant and growing use of SWD, particularly in evaluating public health interventions in facility- and community-based settings. This growth aligns with prior methodological reviews highlighting SWD's suitability for phased implementation and for contexts where withholding potentially beneficial interventions raises ethical concerns (1, 2, 20).

The geographic distribution of included studies, concentrated in Uganda, Kenya, and South Africa, suggests that SWD is more commonly deployed in settings with comparatively stronger research infrastructure and sustained programmatic activity. This pattern is consistent with Hughes et al. (2015) (6). However, unlike Mwai et al. (2013) (111), who reported a more even distribution of research efforts across SSA, our review indicates comparatively fewer SWD studies from West and Central Africa. This gap may reflect differences in capacity, funding, or publication patterns and underscores the need for more targeted SWD research and reporting from these regions.

In terms of health domains, HIV/AIDS accounted for 40% of included studies, consistent with prior reviews (112). This dominance reflects the continuing burden of HIV in SSA and the strong implementation imperative for interventions that cannot ethically be withheld. SWD was frequently used in HIV research to navigate ethical and practical challenges in evaluating interventions where treatment access is considered necessary (7, 13, 113). The sizeable body of work in maternal and child health and tuberculosis similarly reflects urgent regional priorities and supports SWD's applicability for evaluating high-burden conditions in resource-limited settings (16, 114).

Across studies, the design parameters of SWD varied substantially, including study duration (two to sixty months), cluster definitions, numbers of steps and clusters, and step intervals. This variability illustrates SWD's flexibility for diverse implementation contexts and timelines (115). At the same time, inconsistent reporting, particularly for study duration, step intervals, and key operational details, limits reproducibility and makes cross-study comparisons more challenging, echoing concerns that stronger and more consistent reporting standards are needed (116).

Cluster definitions were diverse, spanning health facilities (26, 27, 30–34, 52–71, 73–75, 98–102, 104, 105) and community-based settings (26, 27, 30–34, 49, 50, 73–75, 77–81). This heterogeneity reinforces SWD's versatility, consistent with Eldridge et al. (2019) (117). The frequent use of facilities as clusters suggests a strong focus on service delivery interventions within structured systems, aligning with Hemming et al. (2016) (2). Meanwhile, geographic community-based (26, 49, 50, 73–75, 77–81, 102, 109) and social group clusters (28, 35, 48, 51, 72, 97, 103, 106, 110) demonstrates SWD's utility for capturing broader population-level effects and highlight the importance of context and contamination considerations during design and implementation (6).

The interventions evaluated using SWD were wide-ranging, including digital health technologies (26, 27, 32, 54, 62, 79, 94, 98, 105) and pharmaceutical and biomedical interventions (28, 34, 42, 61, 90, 91, 93, 96) and behavioral/psychosocial interventions (33, 48, 49, 78, 80, 81, 88, 93, 102, 105, 107, 110). Digital interventions—such as the 99DOTS-based TB treatment supervision strategy (26)—illustrate how SWD can assess technology integration into routine care, consistent with broader trends toward digital health (118). While many digital health studies reported positive outcomes (e.g., improved adherence and diagnostic timeliness), results were not uniformly consistent, suggesting that effectiveness may depend on contextual and implementation factors (119). Pharmaceutical and biomedical interventions, including PrEP (93) and SMC (61), were also commonly evaluated using SWD, supporting its utility for phased implementation of large-scale public health strategies in low- and middle-income settings (34, 40, 113). Behavioural and psychosocial interventions—such as Women's Health CoOp (63) and Cognitive Stimulation Therapy (102)—also benefited from SWD's staged rollout, which is often advantageous for interventions that require time to demonstrate effects (120, 121).

Ethical considerations were a major rationale for selecting SWD in many studies (44, 46, 47, 52, 55, 57, 69, 96, 97, 106, 110) onsistent with prior work emphasising the design's capacity to support equity in intervention rollout (6). In particular, SWD offers a pragmatic balance between rigorous evaluation and the ethical imperative to ensure eventual access to potentially beneficial interventions (115). Logistical and operational constraints were also frequently reported as drivers of SWD selection (26, 30, 33, 49, 51, 52, 68, 81, 86, 91, 94, 101) as phased implementation can be more feasible than simultaneous rollout when resources, staffing, training capacity, or supply chains are limited. Studies such as those by Dorothy Thomas and Sophie Sarrassat exemplify how SWD can support multi-site implementation under real-world constraints (40, 101), consistent with broader methodological observations (2, 3, 15, 113).

Another advantage of SWD is its ability to address time-related confounding by explicitly incorporating staggered implementation and repeated measurement. This feature is particularly relevant where secular trends, seasonal variation, or concurrent programs may influence outcomes (36, 37). Studies such as those by Calum Davey (35) and Badara Cisse (34) illustrate the value of accounting for temporal trends to reduce bias, aligning with guidance on SWD's analytical strengths (117).

Sample size considerations in SWD are complex and influenced by the number of clusters and steps, expected effect sizes, and intracluster correlation. Several studies reported detailed sample size calculations [e.g., Adithya Cattamanchi (26) and Alexandra E. Ridout (27)], reflecting rigorous planning consistent with recommendations in the SWD literature (2). However, some studies provided limited detail, likely reflecting real-world challenges in estimating parameters and implementing trials in resource-constrained settings. Pragmatic enrolment approaches were also observed [e.g., Dorothy Thomas (40) and Eduard J. Sanders (41)], underscoring a tension between feasibility and precision in SWD planning (115).

Outcome measures were diverse, spanning clinical outcomes (e.g., HIV, TB, and malaria treatment and incidence measures), behavioural/psychosocial outcomes, process indicators, and diagnostic outcomes. This breadth reinforces SWD's versatility for evaluating both health impacts and system performance within routine care and public health programs (2, 3, 15).

Overall, reporting quality among SWD cluster trials was generally strong, with many studies providing diagrams (26–31, 33–39, 41–48, 50–70, 72–110), detailed intervention descriptions (26–78, 80–110), and robust statistical reporting (26, 27, 29–36, 38, 39, 48, 51, 53–62, 64, 65, 67, 68, 74, 77–82, 103–106, 108, 109). Nonetheless, improvement is still needed in reporting allocation concealment, restricted randomisation procedures, blinding where feasible, and adverse events to strengthen transparency and replicability (115).

Drawing on the diversity of SWD studies included in this review, several pragmatic design considerations emerge for best practice. Clusters should be defined as natural units of intervention delivery (e.g., facilities, districts, schools, communities) and, where contamination or spillover is likely, aligned with geographic/administrative boundaries and supported by stratification or matching on key determinants such as service volume/capacity, baseline performance, or disease burden. The number and duration of steps should balance feasibility and statistical precision: too many steps can prolong trials and increase susceptibility to secular trends and implementation disruption, whereas too few steps may reduce power and limit the ability to model time effects; incorporating buffer/transition periods can improve fidelity where roll-out is complex. Randomisation procedures should be fit-for-purpose and acceptable within the implementation context: statistician-led computer-generated sequences may maximise internal validity, while transparent stakeholder-engaged approaches (e.g., public lotteries) may enhance acceptability for system-level interventions, while maintaining allocation integrity and clear reporting of sequence generation, constraints on concealment, and analytic handling of time-varying effects and intracluster correlation.

The strengths of this review lie in its comprehensive analysis of SWD application across diverse public health interventions in SSA and its attention to ethical and operational considerations relevant to resource-limited settings. However, limitations include the geographic concentration of studies in a few countries, variability in methodological reporting, limited representation of non-communicable diseases, and inconsistent reporting of study durations and intervals, which complicate direct comparisons. The absence of a meta-analysis further limits quantitative synthesis of effects and the ability to estimate pooled effectiveness across interventions.

## Conclusion

This systematic review shows that SWD are increasingly used across SSA to evaluate public health interventions, particularly in HIV/AIDS, maternal and child health, and tuberculosis. Studies were concentrated in a few countries (notably Uganda, Kenya, and South Africa), suggesting uneven adoption across the region and highlighting an evidence gap in West and Central Africa. Overall, SWD was applied to a broad range of intervention types, including digital health, pharmaceutical/biomedical, and behavioural approaches, supporting its adaptability in resource-limited settings where phased implementation is often necessary. However, inconsistent reporting of key design and implementation features such as duration, step structure, concealment, and deviations from planned rollout, limits transparency and cross-study comparability and makes it difficult to judge when operational challenges may compromise outcomes. Future SWD studies in SSA should strengthen reporting and explicitly document implementation disruptions and protocol amendments, and should use context-appropriate design choices for cluster definition, number of steps, and randomisation procedures to minimise contamination, address secular trends, and preserve internal validity. Strengthening adoption and reporting across a wider range of countries and health conditions will improve the usefulness of SWD evidence for policy and implementation.

## Data Availability

The datasets presented in this study can be found in online repositories. The names of the repository/repositories and accession number(s) can be found in the article/Supplementary Material.
