# Supplementary material for: Applications and implementation considerations for stepped-wedge designs in sub-Saharan Africa: a systematic review
Source: Front Epidemiol. 2026 Mar 25;6:1529289. doi: 10.3389/fepid.2026.1529289 (PMC13057542; doi:10.3389/fepid.2026.1529289)
Supplement: Supplementary file 2 [file Supplementaryfile1.pdf]

Search strategy

Cinalh- 59

Keyword	Search strategy	Search option	Result
Stepped wedge	TI "stepped wedge design" OR TI "stepped-wedge" OR TI "step wedge" OR TI "experimentally staged introduction" OR TI "delayed intervention" OR TI "one directional cross over design" OR TI " Sequential Crossover Design" OR TI "Phased Implementation Design"	**Qualifiers** - Full text<br>**Extended Condition** - Applies the equivalent account<br>**Zoom out by SubjectGeographic:** - mozambique<br>**Zoom out by SubjectGeographic:** - burkina faso<br>**Zoom out by SubjectGeographic:** - benin<br>**Zoom out by SubjectGeographic:** - tanzania<br>**Zoom out by SubjectGeographic:** - botswana<br>**Zoom out by SubjectGeographic:** - sub-saharan africa<br>**Zoom out by SubjectGeographic:** - malawi<br>**Zoom out by SubjectGeographic:** - south africa<br>**Zoom out by SubjectGeographic:** - kenya<br>**Zoom out by SubjectGeographic:** - nigeria<br>**Scale-down SubjectGeographic:** - uganda	59

Web of science- 246

	Search strategy	Result
Stepped wedge	((((((TS=("stepped wedge")) OR TS=("step wedge")) OR TS=("experimentally staged introduction")) OR TS=("delayed intervention")) OR TS=("one directional cross over design")) OR TS=("Sequential Crossover Design")) OR TS=("Phased Implementation Design") and Preprint Citation Index (Exclude – Database)	3816
	((((((TS=("stepped wedge")) OR TS=("step wedge")) OR TS=("experimentally staged introduction")) OR TS=("delayed intervention")) OR TS=("one directional cross over design")) OR TS=("Sequential Crossover Design")) OR TS=("Phased Implementation Design") and Preprint Citation Index (Exclude – Database) and ETHIOPIA or MALAYSIA or ZAMBIA or MOZ AMBIQUE or BURKINA	246

	FASO or GHANA or ALGERIA or BENIN or BULGARIA or CO TE IVOIRE or DEM REP CONGO or DOMINICA or EGYPT or CAMBODIA or ARGENT INA or ZIMBABWE or TANZANIA or NIGERIA or MALAWI o r KENYA or UGANDA or SOUTH AFRICA or BOTSWANA or CAMEROON or RWANDA or SIE RRA LEONE or GAMBIA or TOGO or LIBYA or LIBERIA or CAPE TOWN (Countries/Regions)	

Pubmed - 105

	Keyword	Search strategy	Results
#1	Stepped wedge	"stepped wedge"[Title/Abstract] OR "step wedge"[Title/Abstract] OR "delayed intervention"[Title/Abstract] OR "Sequential Crossover Design"[Title/Abstract]	3,142
#2	Africa countries	Algeria or Angola or Benin or Botswana or Burkina Faso or Burundi #3or Cabo Verde or Cameroon or Central African Republic or Chad or Comoros or Democratic Republic of the Congo or Djibouti or Egypt or Equatorial Guinea or Eritrea or Eswatini or Ethiopia or Gabon or Gambia or Ghana or Guinea or Guinea-Bissau or Ivory Coast (Côte d'Ivoire) or Kenya or Lesotho or Liberia or Libya or Madagascar or Malawi or Mali or Mauritania or Mauritius or Morocco or Mozambique or Namibia or Niger or Nigeria or Republic of the Congo or Rwanda or São Tomé and Príncipe or Senegal or Seychelles or Sierra Leone or Somalia or South Africa or South Sudan or Sudan or Tanzania or Togo or Tunisia or Uganda or Zambia or Zimbabwe	124,405
#3	#1 AND #2	((((((("stepped wedge"[Title/Abstract]) OR ("step wedge"[Title/Abstract])) OR ("experimentally staged introduction"[Title/Abstract])) OR ("delayed intervention"[Title/Abstract])) OR ("one directional cross over design"[Title/Abstract])) OR ("Sequential Crossover Design"[Title/Abstract])) OR ("Phased Implementation Design"[Title/Abstract])) AND (Algeria[Title/Abstract] OR Angola[Title/Abstract] OR Benin[Title/Abstract] OR Botswana[Title/Abstract] OR Burkina Faso[Title/Abstract] OR Burundi[Title/Abstract] OR Cabo Verde[Title/Abstract] OR Cameroon[Title/Abstract] OR Central African Republic[Title/Abstract] OR Chad[Title/Abstract] OR Comoros[Title/Abstract] OR Democratic Republic of the Congo[Title/Abstract] OR Djibouti[Title/Abstract] OR Egypt[Title/Abstract] OR Equatorial Guinea[Title/Abstract] OR Eritrea[Title/Abstract] OR Eswatini[Title/Abstract] OR Ethiopia[Title/Abstract] OR Gabon[Title/Abstract] OR Gambia[Title/Abstract] OR Ghana[Title/Abstract] OR Guinea[Title/Abstract] OR Guinea-Bissau[Title/Abstract] OR Ivory Coast (Côte d'Ivoire[Title/Abstract]) OR	105

		Kenya[Title/Abstract] OR Lesotho[Title/Abstract] OR Liberia[Title/Abstract] OR Libya[Title/Abstract] OR Madagascar[Title/Abstract] OR Malawi[Title/Abstract] OR Mali[Title/Abstract] OR Mauritania[Title/Abstract] OR Mauritius[Title/Abstract] OR Morocco[Title/Abstract] OR Mozambique[Title/Abstract] OR Namibia[Title/Abstract] OR Niger[Title/Abstract] OR Nigeria[Title/Abstract] OR Republic of the Congo[Title/Abstract] OR Rwanda[Title/Abstract] OR São Tomé[Title/Abstract] AND Príncipe[Title/Abstract] OR Senegal[Title/Abstract] OR Seychelles[Title/Abstract] OR Sierra Leone[Title/Abstract] OR Somalia[Title/Abstract] OR South Africa[Title/Abstract] OR South Sudan[Title/Abstract] OR Sudan[Title/Abstract] OR Tanzania[Title/Abstract] OR Togo[Title/Abstract] OR Tunisia[Title/Abstract] OR Uganda[Title/Abstract] OR Zambia[Title/Abstract] OR Zimbabwe[Title/Abstract])	

COCHRANE- 379

COHRANE -379

Search Name: STEPPED WEDGE DESIGN

Date Run: 02/04/2024 02:19:22

Comment:

ID Search Hits

#1 "stepped wedge" 2743

#2 "step wedge" 111

#3 "delayed intervention" 710

#4 "one directional cross over design" 0

#5 "Sequential Crossover Design" 9

#6 "Phased Implementation Design" 0

#7 #1 OR #2 OR #3 OR #4 OR #4 OR #5 OR #6 3539

#8 Algeria or Angola or Benin or Botswana or Burkina Faso or Burundi or Cabo Verde or Cameroon or Central African Republic or Chad or Comoros or Democratic Republic of the Congo or Djibouti or Egypt or Equatorial Guinea or Eritrea or Eswatini or Ethiopia or Gabon or Gambia or Ghana or Guinea or Guinea-Bissau or Ivory Coast (Côte d'Ivoire) or Kenya or Lesotho or Liberia or Libya or Madagascar or Malawi or Mali or Mauritania or Mauritius or Morocco or Mozambique or Namibia or Niger or Nigeria or Republic of the Congo or Rwanda or São Tomé and Príncipe or Senegal or Seychelles or Sierra Leone or Somalia or South Africa or South Sudan or Sudan or Tanzania or Togo or Tunisia or Uganda or Zambia or Zimbabwe 39988

#9	#7 AND #8	379

PsychINFO-17

https://psycnet.apa.org/permalink/f800b421-78e6-cd6a-e5c6-874784eeaef3
